# The Differential Involvement of α1-Adrenoceptor Subtypes in the Molecular Effects of Antidepressant Drugs

**DOI:** 10.3390/ijms262110488

**Published:** 2025-10-28

**Authors:** Irena Nalepa, Katarzyna Chorązka, Grzegorz Kreiner, Agnieszka Zelek-Molik, Anna Haduch, Władysława Anna Daniel, Piotr Chmielarz, Katarzyna Maziarz, Justyna Kuśmierczyk, Michał Wilczkowski, Adam Bielawski, Marta Kowalska

**Affiliations:** 1 Department of Brain Biochemistry, Maj Institute of Pharmacology, Polish Academy of Sciences, Smętna 12, 31-343 Kraków, Polandkreiner@if-pan.krakow.pl (G.K.); zelek@if-pan.krakow.pl (A.Z.-M.); chmiel@if-pan.krakow.pl (P.C.); maziarz@if-pan.krakow.pl (K.M.); justyna.kusmierczyk@awf.krakow.pl (J.K.); wilczkow@if-pan.krakow.pl (M.W.); bielaw@if-pan.krakow.pl (A.B.); marcik48@op.pl (M.K.); 2 Department of Pharmacokinetics and Drug Metabolism, Maj Institute of Pharmacology, Polish Academy of Sciences, Smętna 12, 31-343 Kraków, Poland; haduch@if-pan.krakow.pl (A.H.); nfdaniel@cyf-kr.edu.pl (W.A.D.)

**Keywords:** desipramine, milnacipran, knockout mice, quantitative autoradiography, UHPLC, mRNA, DNA microarray, ERK1/2, Akt, GSK3β

## Abstract

We investigated whether the functional involvement of α1-adrenergic receptors (α1-AR) in the effects induced by antidepressant drugs, desipramine, and milnacipran varies depending on the α1-AR subtype. First, using a mouse line with triple knockout (KO) of genes encoding all three α1-AR subtypes (ABD-KO) and autoradiographic analysis, we demonstrated that the inactivation of α1-AR did not affect the density of other types of adrenergic receptors, α2- and β-AR in the mouse brain. Subsequently, we utilized three mouse knockout lines with selective knockout of the gene encoding a single α1-adrenergic receptor subtype (A-KO, B-KO, and D-KO). We analyzed the impact of these mutations on tissue levels of monoaminergic neurotransmitters in the hypothalamus (HY). Next, we assessed how a specific mutation affects the chronic effects of desipramine and milnacipran in the selected brain regions of male and female mice at various molecular levels: mRNA expression of genes encoding for α1-AR subtypes, gene expression profiling, and phosphorylation of selected signaling proteins (ERK1/2, Akt, GSK3β). The main finding is that the deletion of the α1D subtype predominantly reduced the chronic effects of milnacipran at the examined transcriptomic and proteomic levels. The pattern of changes differed by gender. Our study revealed the functional diversity between α1-AR subtypes in the molecular mechanisms of antidepressants’ drug action.

## 1. Introduction

Noradrenaline is the neurotransmitter primarily released from the locus coeruleus neurons. It functions to increase alertness, attention, and arousal, and plays a crucial role in memory formation and retrieval. Noradrenaline is also an important regulator of behavioral and physiological stress responses [1,2,3]. The actions of noradrenaline result from the activation of different adrenoceptors, which are widely distributed in the central and peripheral nervous systems. These receptors are the seven transmembrane-spanning receptors that belong to the large G-protein-coupled receptor (GPCR) superfamily. The adrenergic receptors are classified into three subfamilies: α1-adrenoceptors (α1-ARs), α2-AR-adrenoceptors (α2-ARs), and β-adrenoceptors (β-ARs), based on their pharmacological profiles and coupling to G proteins and signaling pathways [4,5,6]. Each of these subfamilies consists of three receptor subtypes.

The α1-AR subfamily was the subject of the present work. When considering the subtypes of α1-AR, namely α1A-AR, α1B-AR, and α1D-AR, some similarities can be observed among them; however, there are also noticeable differences. All three receptor subtypes are coupled to Gq/11 and phospholipase Cβ (PLC), which stimulate phosphoinositide hydrolysis to produce two second messengers, inositol trisphosphate (IP3) and diacylglycerol (DAG). IP3 promotes the release of Ca^2+^ from intracellular stores. DAG activates protein kinase C (PKC), which can phosphorylate numerous other proteins and trigger downstream signaling in the cascade. The α1-ARs function as stimulatory receptors. However, each subtype is encoded by a separate gene on different chromosomes, has a distinct pharmacological profile and amino acid sequence, and is differentially distributed [7,8,9].

Although all three subtypes of α1-AR activate the same main signaling pathway, the profiles of gene expression changes induced within the cell through their signaling may not always be identical. Differences between individual subtypes of α1-ARs were also observed in their regulation of activity following stimulation with their physiological agonist, noradrenaline, or synthetic agonistic compounds [10,11,12,13,14]. This partly determines the profile of intracellular changes following stimulation of specific α1-AR subtypes.

Furthermore, it appears that there are ligand-dependent phosphorylation patterns of α1-ARs [11,15], which should be considered when exploring the mechanisms of drug action. Ultimately, the α1-ARs can signal through either G-protein-dependent or G-protein-independent mechanisms involving β-arrestins. The latter act as scaffolds to recruit and activate other second messengers, such as extracellular signal-regulated kinases 1/2 (ERK 1/2), p38, and Src [16,17]. Moreover, in addition to initiating independent signaling pathways, β-arrestins can also terminate GPCR signaling [7].

The diverse distribution and functions of α1-AR subtypes in peripheral tissues are relatively well characterized, particularly in the cardiovascular and urogenital systems [7,18,19,20,21,22]. However, these α1-AR subtypes are less explored when considering the central nervous system. While it is known that these receptors are expressed in the brain [23], attempts to determine the localization and physiological function of individual α1-AR subtypes have been challenging. In addition to the lack of highly selective ligands and specific antibodies, a significant limitation has been the blood–brain barrier’s selective permeability, which is often not exceeded for many chemical compounds including α1-AR antagonists or agonists.

With methodological progress, diverse expression patterns of α1-AR subtypes in the brain have been identified through genetic engineering and the construction of transgenic mouse models with fluorescently tagged receptors, as well as knockout (KO) mice. Studies using these approaches have shown that the α1A and α1B subtypes are widely distributed in brain tissue, with their highest expression observed primarily in neurons of structures such as the cerebral cortex, amygdala, hypothalamus (HY), and cerebellum [24,25]. In contrast, the α1D subtype exhibits low expression levels in the brain and is mainly localized in the cerebral cortex, hippocampus, and specific thalamic nuclei [26].

For many years, cerebral α1-ARs have attracted research interest in studying the neurochemical and molecular mechanisms of how antidepressant drugs work. Tricyclic antidepressants (TCA) often directly interact with these receptors, exhibiting affinity and binding to α1-ARs. Such a TCA action is not limited to brain receptors but also affects peripherally located α1-ARs, leading to unwanted cardiovascular effects associated with these drugs (see [27]). A common serious cardiovascular problem linked with many TCA is orthostatic hypotension, mainly caused by decreased myocardial contractility and reduced systemic vascular resistance due to α-adrenergic blockade [28]. On the other hand, the controlled adrenolytic effect of α1-AR blockers provides benefits by alleviating symptoms of prostatic hyperplasia, and these drugs are also used in the treatment of hypertension [29,30]. Analyzing the specific peripheral adverse effects of TCA is beyond this article’s scope, which focuses on the psychotropic properties of these drugs and their brain effects at the molecular level. However, it should be noted that these drugs also have antagonistic properties toward histaminergic H1-, H2-, and muscarinic M-receptors, which expands the list of undesirable effects. Other classes of antidepressants, including selective serotonin reuptake inhibitors (SSRIs) and the dual-action serotonin-noradrenaline reuptake inhibitors (SNRIs), among them milnacipran (MIL), generally possess minimal affinity for monoaminergic and muscarinic receptors, and are devoid of the majority of side effects relevant to TCA [31].

However, the primary pharmacological target of most antidepressants is the reuptake process of catecholamines and serotonin (into neurons), and this may lead to the enhanced postsynaptic stimulation of α1-ARs (see [32]). In our previous studies, the repeated administration of the antidepressant drug imipramine or electroconvulsive treatment for at least 3 weeks increased the density of α1A-ARs in a rat model. Also, it differentially altered the mRNA expression of genes encoding α1-AR subtypes. Whereas both treatments elevated mRNA expression of the gene encoding α1A-AR in the prefrontal cortex, the mRNA for α1B-AR remained unchanged [33]. This pattern of changes suggested that receptor subtypes are differentially involved in the molecular mechanism of action of antidepressant drugs.

While initial hypotheses about the cause of depression linked it to impairment of monoaminergic pathways in the brain, one of the widely accepted current theories suggests that disrupted neuroplasticity underlies depressive disorders [8,32,34]. Imaging and postmortem brain studies in depressed human subjects and animal behavioral models of depression have identified several different cellular events and intracellular signaling pathways that are modulated by stress and are potential mediators of antidepressant action [35]. Given the numerous changes in intracellular signaling that occur upon stimulation of monoaminergic receptors, including α1-ARs and their subtypes, prolonged and frequent stimulation of these receptors can lead to adaptive changes that influence neuroplasticity.

Among intracellular signaling pathways implicated in mood disorders are ERK 1/2, the central MAPK proteins [36] that were shown to be downregulated in the hippocampus of depressed suicide subjects [37]. As our previous studies in the rat model have shown, chronic treatment with imipramine increases the level of phosphorylated form of ERK1, indicating enhanced activation of ERK1 by imipramine [38]. Other intracellular signaling pathways implicated in neuropsychiatric disorders include phosphatidylinositol 3-kinase (PI3K)-Akt signaling and the glycogen synthase kinase-3 beta (GSK3β) signaling cascade (see 36]), and the subtypes of the α1-AR have been shown to be involved in the regulatory mechanisms of these proteins [39,40,41,42].

In the present study, we investigated whether the functional involvement of α1-AR in the effects induced by chronic treatment with antidepressant drugs, desipramine (DMI) and MIL, varies depending on the α1-AR subtype. DMI and MIL were chosen as representative drugs of different mechanistic approaches. DMI exhibits much more potent inhibition of noradrenaline reuptake than serotonin, while MIL belongs to the SNRI (serotonin-noradrenaline reuptake inhibitor) group [31,43]. The study utilized three mouse knockout lines with selective inactivation of the gene encoding a single α1-AR subtype (A-KO, B-KO, and D-KO). We assessed whether and how a specific mutation affects the chronic effects of DMI and MIL in selected brain regions of male and female mice at various molecular levels, including mRNA expression of α1-AR subtypes, gene expression profiling, and expression and activation of selected signaling proteins (ERK1/2, Akt, GSK3β).

## 2. Results

### 2.1. Autoradiographic Analyses of the Density of α1-AR, α2-AR, and β-AR Receptors in the Brains of Triple ABD-KO Mice

**Figure 1:**
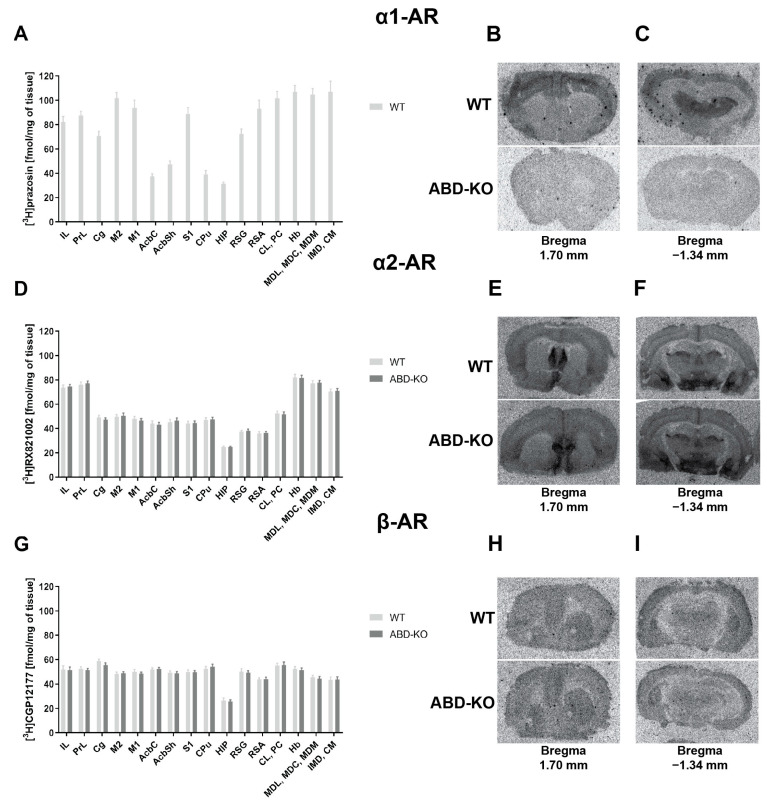
The density of α1-adrenergic receptors (α1-AR; [^3^H]prazosin binding) (**A**–**C**), α2-adrenergic receptors (α2-AR; [^3^H]-RX821002 binding) (**D**–**F**), and β-adrenergic receptors (β-AR; [^3^H]CGP12177 binding) (**G**–**I**) in selected brain structures of wild-type (WT) mice and mice with knockout of all three α1-AR subtypes (ABD-KO). Left panels (**A**,**D**,**G**)—the data represent mean specific signal values (fmol/mg of tissue ± SEM) from *n* = 9 sections, analyzed using one-way analysis of variance (ANOVA) and post hoc Fisher’s Least Significant Difference (LSD) test. Light gray bars represent WT mice (littermates group), and dark gray bars represent ABD-KO mutants. Right panels: (**B**,**C**,**E**,**F**,**H**,**I**)—examples of autoradiographic images showing the binding of radioligands to α1-AR (**B**,**C**), and α2-AR (**E**,**F**), and β-AR (**H**,**I**) in the brains of WT mice and mice with knockout of all three α1-AR subtypes (ABD-KO). Abbreviations: IL—infralimbic cortex, PrL—prelimbic cortex, Cg—cingulate cortex, M2—secondary motor cortex, M1—primary motor cortex, AcbC—accumbens nucleus, core, AcbSh—accumbens nucleus, shell, S1—primary somatosensory cortex, CPu—caudate putamen (striatum), HIP—hippocampus, RSG—retrosplenial granular cortex, RSA—retrosplenial agranular cortex, CL—centrolateral thalamic nucleus, PC—paracentral thalamic nucleus, MDL—mediodorsal thalamic nucleus, lateral part, MDC—mediodorsal thalamic nucleus, central part, MDM—mediodorsal thalamic nucleus, medial part, IMD—inter mediodorsal thalamic nucleus, CM—central medial thalamic nucleus.

The ABD-KO transgenic mouse line was examined for the effectiveness of mutations affecting α1-AR, based on the number of [^3^H]-prazosin binding sites and how mutations influenced other AR subclasses. Quantitative in vitro receptor autoradiography was performed on brain sections from male ABD-KO and wild-type (WT) mice, analyzing the distribution and density of binding sites for α1-AR, α2-AR, and β-AR receptors. The analysis was performed on brain sections corresponding to the bregma levels of +1.70 mm (Supplementary Figure S1A) and −1.34 mm (Supplementary Figure S1B), chosen according to literature data and our previous studies on the localization and abundance of adrenergic receptors in laboratory rodent brains, especially α1-AR, which shows high expression in regions such as the cerebral cortex, hippocampus, and thalamus [44,45,46,47,48]. Quantitative autoradiography of all types of adrenergic receptors is presented in Figure 1.

In WT mice, the [^3^H]prazosin α1-AR binding sites were found in the highest amounts in the cerebral cortical regions and thalamic nuclei, with moderate levels in the accumbens nuclei and hippocampus (Figure 1A). Conversely, the highest densities of α2-AR were observed in the infralimbic cortex, prelimbic cortex, and thalamic nuclei, with lower densities across other cortical regions, the accumbens, and hippocampus, as determined by [^3^H]RX821002 autoradiography (Figure 1D). Unlike the α1-ARs, the β-ARs—measured as [^3^H]CGP12177 binding sites—showed similar levels across nearly all examined brain regions. An exception was noted in the hippocampus, where the density of β-ARs was half that of the other brain regions studied (Figure 1G).

In the case of α1-AR density measurements in ABD-KO mice, a signal originating exclusively from non-specific background binding was observed (Figure 1B,C, lower panels). The absence of specific signals in the analyzed brain structures of ABD-KO mice indicates either an absence or trace amounts of [^3^H]-prazosin binding sites, confirming the effectiveness of the mutation-induced inactivation of all three subtypes of α1-AR. The bars in Figure 1A depict the α1-AR density level in the brains of only WT controls. Significantly, the ABD-KO mutation did not affect the density of α2-AR (Figure 1D–F) and β-AR (Figure 1G–I), which did not differ from the density of these receptors in the examined brain structures of the WT control mice.

### 2.2. Neurotransmitters and Their Metabolite Levels in the Hypothalamic Tissues of Genetically Modified Mice

**Figure 2:**
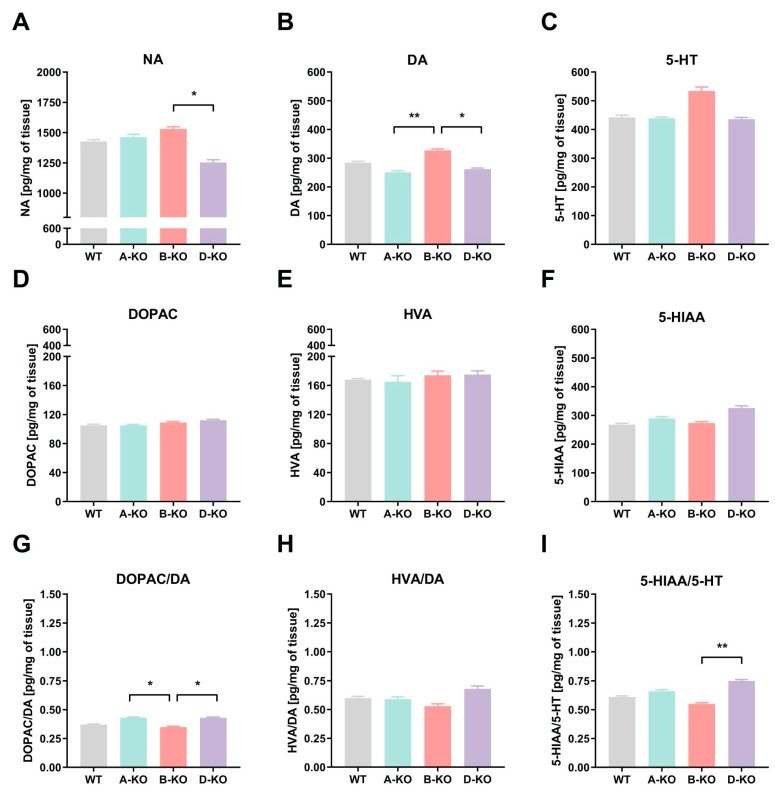
Levels of noradrenaline (NA) (**A**), dopamine (DA) (**B**), serotonin (5-HT) (**C**), 3,4-dihydroxyphenylacetic acid (DOPAC) (**D**), homovanillic acid (HVA) (**E**), and 5-hydroxy indole acetic acid (5-HIAA) (**F**), as well as turnover of DA (**G**,**H**) and 5-HT (**I**) in the hypothalamus (HY) of wild-type (WT) mice and knockout mice with selective inactivation of α1A adrenergic receptor (A-KO), α1B adrenergic receptor (B-KO), or α1D adrenergic receptor (D-KO). Data represent mean values (pg/mg tissue ± SEM) from *n* = 7–8, analyzed using one-way analysis of variance (ANOVA) and post hoc Tukey’s test for unequal sample sizes (Unequal N HSD). * *p* < 0.05, ** *p* < 0.01.

The characterization of mouse transgenic lines A-KO, B-KO, and D-KO began with examining the effects of mutations on levels of noradrenaline, dopamine, its metabolites such as 3,4-dihydroxyphenylacetic acid (DOPAC) and homovanillic acid (HVA), as well as serotonin and its metabolite 5-hydroxyindoleacetic acid (5-HIAA) in the HY of male mice. The tissue levels of neurotransmitters were measured using ultra-high-performance liquid chromatography (UHPLC) with coulochemical detection (Figure 2A–F). Additionally, the results were presented as ratios of metabolite concentrations to their corresponding analytes, allowing for the assessment of how the mutations affected neurotransmitter levels and their metabolism (turnover) in the examined tissues (Figure 2G–I).

The analysis of the data using one-way ANOVA showed the effect of deleting individual α1A-AR, α1B-AR, or α1D-AR subtypes on tissue levels of noradrenaline (genotype effect F(3, 26) = 4.3404, *p* = 0.01316), dopamine (genotype effect F(3, 24) = 5.3497, *p* = 0.00577), and serotonin (genotype effect F(3, 24) = 3.1527, *p* = 0.04335) in the HY of mice (Figure 2A–C). The post hoc analysis showed no differences in neurotransmitter levels compared to the control group, which consisted of WT littermates from the respective transgenic breeding lines. However, in the HY of D-KO mice, noradrenaline and dopamine levels were significantly lower compared to B-KO mice (Figure 2A,B). Deleting any of the α1-AR subtypes did not affect the levels of the investigated metabolites in this brain structure (Figure 2D–F). However, the mutation affected the ratios of DOPAC to dopamine concentrations (effect of genotype F(3, 24) = 4.5336, *p* = 0.01179) and 5-HIAA to 5-HT concentrations (effect of genotype F(3, 23) = 5.0017, *p* = 0.00815) (Figure 2G,I).

### 2.3. The Impact of α1-AR Subtype-Specific Deletions and Antidepressant Drugs on the mRNA Levels of Remaining α1-AR Subtypes

To obtain a picture of potential transcript level compensatory effects that the deletion of a particular α1-AR subtype on other subtypes might cause, we have determined relative mRNA expression levels of genes encoding individual α1-AR subtypes in the prefrontal cortex (PFC) of male α1A-AR (A-KO), α1B-AR (B-KO) and α1D-AR (D-KO) knockout animals. The quantification was performed in animals repeatedly injected with saline, DMI, or MIL. The mRNA levels of the *ADRA1A*, *ADRA1B*, and *ADRA1D* genes were quantified using the quantitative real-time polymerase chain reaction (qRT-PCR) method.

**Figure 3:**
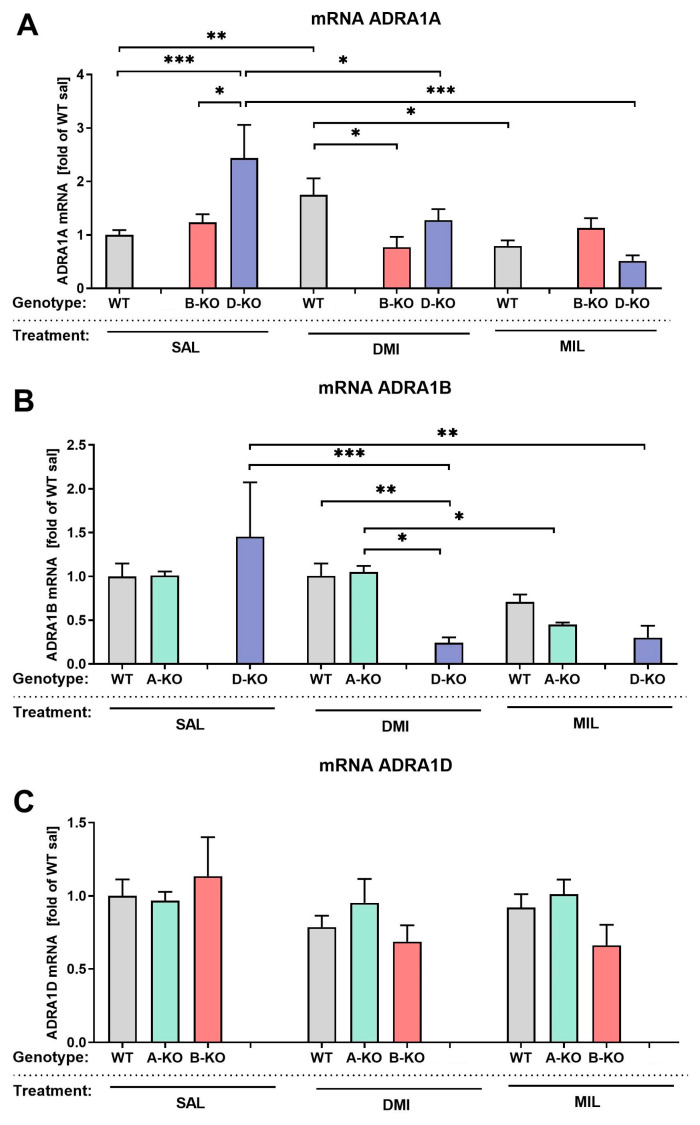
Levels of mRNA expression of the *ADRA1A* (**A**), *ADRA1B* (**B**), and *ADRA1D* (**C**) genes in the prefrontal cortex of wild-type (WT, gray bars) mice and knockout mice with selective inactivation of α1-drenergic receptor (A-KO, green bars), α1B-adrenergic receptor (B-KO, red bars), or α1D-adrenergic receptor (D-KO, violet-blue bars), treated with saline (sal), desipramine (DMI), or milnacipran (MIL). Data represent mean values ± SEM from *n* = 5–23, analyzed using two-way analysis of variance (ANOVA) and post hoc Fisher’s Least Significant Difference (LSD) test. * *p* < 0.05, ** *p* < 0.01, *** *p* < 0.001.

The results show that, for mRNA levels of the genes encoding α1A-AR (*ADRA1A*) and α1B-AR (*ADRA1B*), deletion of the α1D-AR subtype and repeated antidepressant drug treatment produce similar effects (Figure 3A,B). Both *ADRA1A*and *ADRA1B*mRNA levels increased (α1A-AR, Figure 3A) or tended to increase (α1B-AR, Figure 3B) in saline-injected D-KO animals, and both DMI and MIL treatments reversed this. A two-way ANOVA showed that the antidepressant drug alone affected the mRNA levels of these genes (*ADRA1A*: drug effect F(2, 86) = 4.258, *p* = 0.0172; *ADRA1B*: drug effect F(2, 88) = 6.212, *p* = 0.0030), as did the combination of mutation and repeated antidepressant treatment (*ADRA1A*: interaction effect F(4, 86) = 4.713, *p* = 0.0017; *ADRA1B*: interaction effect F(4, 88) = 2.870, *p* = 0.0276). For the gene encoding α1D-AR (*ADRA1D*), neither deletion of *ADRA1A* nor *ADRA1B* nor repeated antidepressant treatment influenced its mRNA expression in the mice’s PFC compared to the control group of WT individuals from the respective transgenic lines (Figure 3C).

### 2.4. Assessment of the Impact of α1-AR Subtype Deletions and Antidepressant Drugs on the Gene Expression Profile

**Figure 4:**
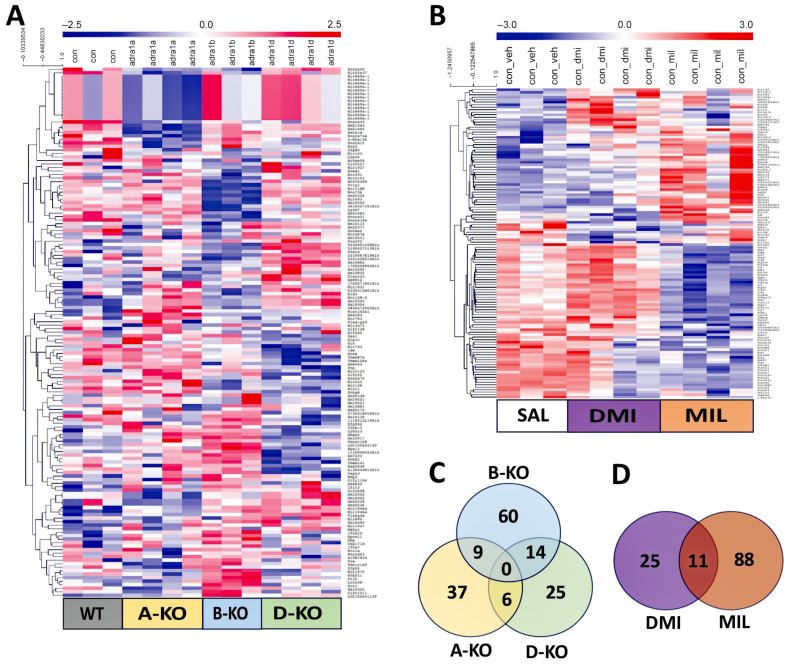
Heat maps illustrating gene expression patterns in the hippocampus of wild-type (WT) mice and knockout mice with selective inactivation of the α1A (A-KO), α1B (B-KO), or α1D (D-KO) adrenergic receptors (**A**), along with WT mice treated with physiological saline (sal), desipramine (DMI), or milnacipran (MIL) (**B**). The Venn diagrams show the number of transcripts with significant expression changes based on mouse genotype (**C**) or the administered antidepressant drug (**D**). Log twofold changes in transcripts’ expression are displayed as heat maps. The color scale indicates the level of log twofold change, with blue representing low values, red representing high values, and white indicating no change. The intensity of the colored rectangles reflects the level of change. The displayed level is proportional to the row z-score value, between −2.5 and 2 for (**A**) and −3 and 3 for (**B**), as indicated by the bar at the top of the heat map images. The significance threshold was set at *p* < 0.05 and fold change > 1.5 (log2).

The impact of mutations and repeated administration of DMI or MIL on gene expression profiles in the hippocampus of A-KO, B-KO, and D-KO mice was investigated using DNA microarray technology. The data are presented as heat maps illustrating gene expression patterns (Figure 4).

Based on the analysis of the gene expression profiling results, a total of 151 transcripts were found to be significantly altered due to the deletion of one of the α1-ARs subtypes. Comparable numbers of transcripts were affected by the deletion of α1A-AR or α1D-AR (52 in A-KO, 45 in D-KO), while the deletion of α1B-AR resulted in nearly twice as many affected transcripts (83 in B-KO). Some significantly regulated transcripts were shared between two of the three transgenic lines (Supplementary Table S1). However, no transcripts were found to be significantly regulated in both A-KO and B-KO or D-KO mice simultaneously (Figure 4C). Furthermore, analysis of gene expression patterns presented in heat maps using clustering methods also indicates a distinct gene expression profile in the hippocampus of B-KO mice compared to A-KO and D-KO mice (Figure 4A).

Among the nine transcripts significantly regulated in both A-KO and B-KO, there were Ifna15 (interferon alpha 15)—orthologous to several human genes (including IFNA1-interferon alpha 1, IFNA13-interferon alpha 13, and IFNA14-interferon alpha 14), and Hpcal1—hippocalcin-like 1 (a member of the neuron-specific calcium-binding proteins family found in the retina and brain). Others included a few transcripts of genes of unknown type (predicted genes) (Supplementary Table S1). In mice A-KO and D-KO, six transcripts were similarly regulated, including Gnb2l1 (RACK1)-guanine nucleotide binding protein and Pnp—purine–nucleoside phosphorylase. Fourteen transcripts were co-regulated in B-KO and D-KO mice. Here, the list included Prlh (prolactin-releasing hormone), Ifit3 (interferon-induced protein with tetratricopeptide repeats 3), Pcbd1 (pterin 4 alpha carbinolamine dehydratase), Hmgn5 (high-mobility group nucleosome binding domain 5), Rps11 (ribosomal protein S11), and Pla2g4e (phospholipase A2). Other transcripts were microRNAs or predicted genes. The impact of chronic administrations of antidepressant drugs on gene expression profiles in the hippocampus of WT mice was demonstrated for both investigated antidepressants, with a more pronounced effect observed with MIL (99 significantly regulated transcripts) compared to DMI (36 significantly regulated transcripts). Analysis of gene expression patterns also indicates a distinct impact of multiple administrations of DMI and MIL on gene expression profiles in the hippocampus of WT mice (Figure 4B). Similar to the mutation effect, a small number of transcripts were found to be regulated by both drugs (Figure 4D). Among these were mainly small nucleolar RNAs (Snora68, Snord58b, Snord16a, Snora16a, Snora15, Snora34, Scarna6) and Cep83os (centrosomal protein 83) (Supplementary Table S2).

Additionally, ontological gene analysis was conducted on transcripts significantly affected by multiple MIL treatments, revealing their roles in processes such as ceramide biosynthesis, oligodendrocyte differentiation, and glycolipid metabolism (Supplementary Figure S2).

**Figure 5:**
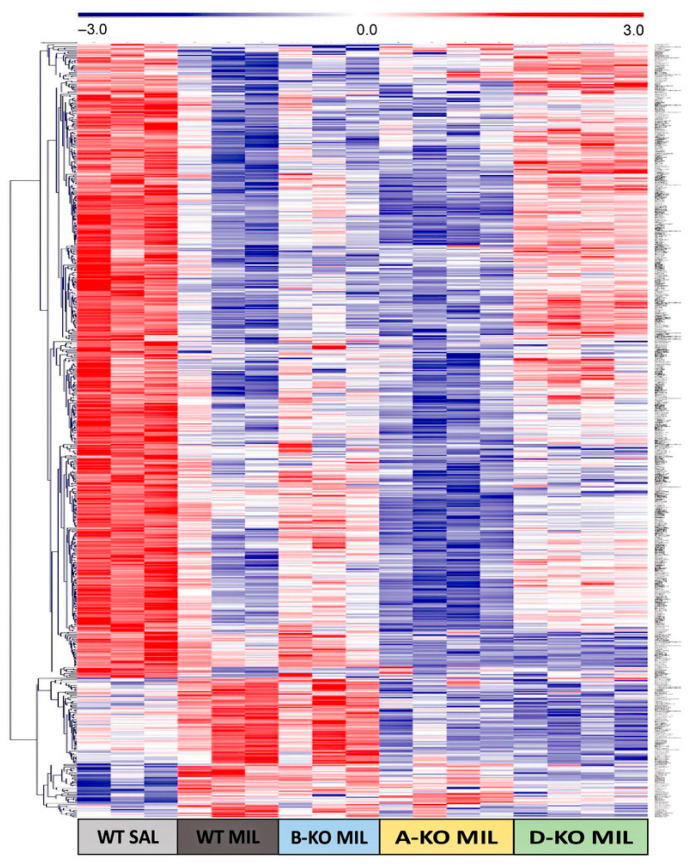
The heat map of gene expression patterns in the hippocampus of wild-type (WT) mice and knockout mice with selective inactivation of α1A-adrenoceptor (A-KO), α1B-adrenoceptor (B-KO), or α1D-adrenoceptor (D-KO), treated with either physiological saline (sal) or milnacipran (MIL). Log twofold changes in transcripts’ expression are displayed as the heat map. The color scale indicates the level of log twofold change, with blue representing low values, red representing high values, and white indicating no change. The intensity of the colored rectangles reflects the level of change. The displayed level is proportional to the row z-score value, between −3 and 3, as indicated by the bar at the top of the heat map. The significance threshold was set at *p* < 0.05 and fold change > 1.5 (log2).

Due to the markedly more significant effect at the transcriptomic level of multiple administrations of MIL compared to DMI, the impact of deleting individual α1-AR subtypes and antidepressant drugs action on gene expression profiles in the hippocampus of mice was decided to be presented in more detail using the example of MIL results (Figure 5). Analysis of gene expression patterns corresponding to transcripts significantly regulated by MIL in WT mice (WT MIL group) and WT (WT sal) and A-KO, B-KO, and D-KO mice receiving MIL administrations indicates the abolition of drug effects due to deletion of α1D-AR, with these changes not observed in the case of α1A-AR or α1B-AR deletions. Furthermore, the gene expression profile in B-KO mice after MIL administration is notably similar to the gene expression profile in WT mice after MIL administration, suggesting that at the transcriptomic level, the α1B subtype (unlike the α1D subtype) does not influence the effects of MIL observed in WT mice compared to WT mice.

On the other hand, the deletion of α1A-AR appears to intensify the impact of MIL observed in WT MIL animals compared to WT SAL animals, although these changes are not unequivocal (Figure 5).

To explore the expression profiling data regarding the effects of MIL and its putative attenuation by KO of α1D-AR (as shown in the profiling data), we used the DAVID Functional Annotation Bioinformatics Microarray Analysis to further analyze the significantly under- and overrepresented genes upon treatment with MIL. The analysis revealed 17 KEGG (Kyoto Encyclopedia of Genes and Genomes) pathways affected by the drug. These pathways are barely represented in the DAVID analysis when compared 271 genes meeting the same criteria of significance and fold change ratio (*p* < 0.05, fold > 0.5/<−0.5) between control samples and the α1D-AR KO group treated with MIL (D-KO MIL). When comparing these mice, the analysis revealed only 6 KEGG pathways affected by the drug, with only 2 KEGG pathways overlapping (Supplementary Figure S3). Interestingly, most KEGG pathways identified after MIL treatment are linked to intracellular processes associated with neurodegenerative diseases. Of course, MIL, as the SNRI, does not directly influence neurodegeneration. However, it can alter neurotransmitter levels, which are essential to understand within the broader context of neurodegeneration and related conditions. In particular, it is well known that MIL may impact neurotrophic factors crucial for neuronal survival and function. The biological implications of this phenomenon remain to be analyzed in future studies.

### 2.5. Evaluation of the Impact of α1-AR Subtypes’ Deletions and Repeatedly Given Antidepressant Drugs on the Phosphorylation of Selected Protein Kinases

To assess the impact of mutations and repeated administration of DMI or MIL on intracellular signaling pathways, the levels of phosphorylation and expression of several proteins involved in α1-AR signaling or considered as intracellular targets of antidepressant drug action were measured, including ERK1/2, Akt, and GSK3β. These assessments were conducted using Western blot analysis in the hippocampi of male and female A-KO, B-KO, and D-KO mice and their WT littermates from the respective transgenic lines.

**Figure 6:**
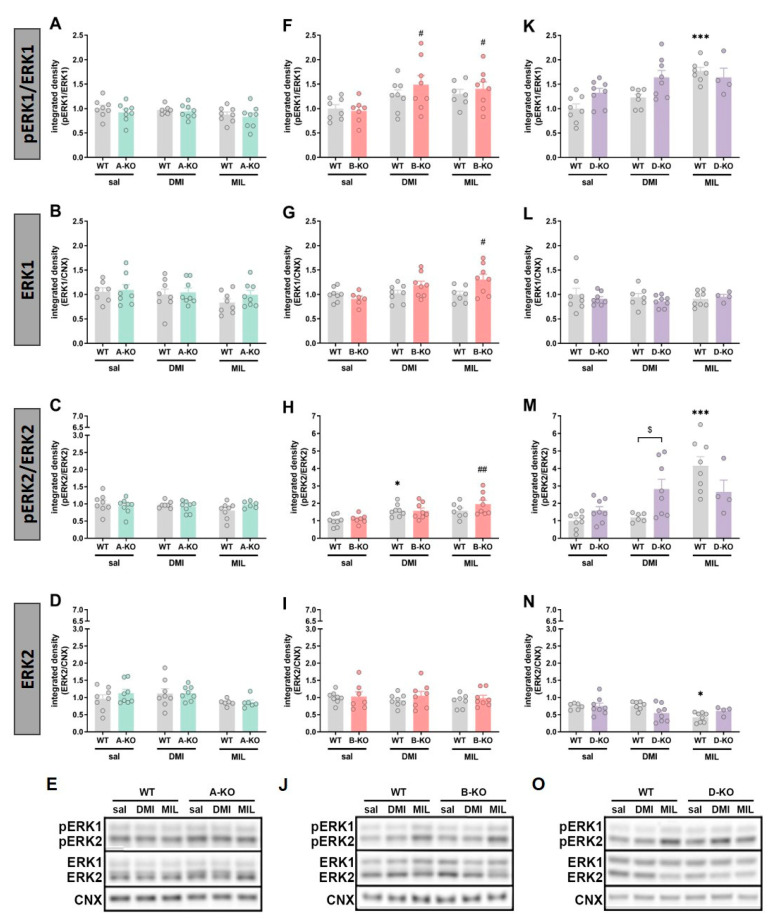
Impact of repeatedly given antidepressant drugs, desipramine (DMI) or milnacipran (MIL), and the mutation effect on phosphorylation and protein levels of ERK1 and ERK2 in the hippocampus of male wild-type (WT, gray bars) mice and knockout mice with selective inactivation of α1A-adrenoceptor (A-KO, green bars) (graphs (**A**–**E**)), α1B-adrenoceptor (B-KO, red bars) (graphs (**F**–**J**)), and α1D-adrenoceptor (D-KO, violet bars) (graphs (**K**–**O**)), are presented. Representative immunoblot images are shown for each protein and subtype of the receptor tested (**E**,**J**,**O**). The mutual representative blot for CNX is visualized in panels (**E**,**J**,**O**), in both Figure 6 and Figure 7. CNX—cyclophilin, a housekeeping gene. Data are shown as mean values ± SEM with *n* = 4–8, analyzed using two-way analysis of variance (ANOVA) and Tukey’s post hoc test for unequal group sizes (Unequal N HSD). * *p* < 0.05 vs. WT sal, *** *p* < 0.001 vs. WT sal, ^#^ *p* < 0.05 vs. KO sal, ^##^ *p* < 0.01 vs. KO sal, ^$^ *p* < 0.05 vs. WT drug.

In male mice, selective deletion of any of the three subtypes (A, B, and D) of the α1-AR receptor alone did not cause changes in the phosphorylation of ERK1 (Figure 6A,F) and ERK2 (Figure 6C,H,M) compared to WT animals, except for a slight increase in the phosphorylated form of ERK1 in D-KO animals (Figure 6K). However, changes were observed following chronic administration of antidepressant drugs and were limited to the α1B-AR and the α1D-AR.

In B-KO mice, both DMI and MIL treatment increased the ERK1 phosphorylation (pERK1/ERK1 ratio, Figure 6F), [pERK1/ERK1: DMI effect F(1, 27) = 10.203, *p* = 0.00355, and MIL effect F(1, 26) = 12.597, *p* = 0.0015]. However, this result should be interpreted with caution because both treatments influenced total ERK protein levels, and the observed increase in ERK1 phosphorylation may be due to increased total ERK1 protein levels. Thus, chronically given MIL significantly increased total ERK1 protein expression in the hippocampi of B-KO mice compared to WT individuals [MIL effect F(1, 25) = 6.5788, *p* = 0.01671, and B-KO-genotype × MIL interaction effect F(1, 25) = 6.4607, *p* = 0.01760]. Moreover, statistical analysis of the results also indicated the main effect of DMI [F(1, 26) = 4.9864, *p* = 0.0343].

In the case of ERK2 phosphorylation, the pERK2/ERK2 ratio was enhanced by MIL in hippocampi of B-KO mice after the chronic drug administration, compared to B-KO-saline group (Figure 6H), [MIL effect: F(1, 26) = 20.279, *p* = 0.00012]. Meanwhile, DMI augmented the pERK2/ERK2 in the WT mice compared to the WT-saline injected group [DMI effect: F(1, 27) = 17.576, *p* = 0.00027].

In D-KO mice, the impact of the two drugs varied. Chronic DMI did not induce changes in the hippocampus of WT mice; however, it enhanced the phosphorylation of ERK1 and ERK2 in D-KO individuals [pERK1/ERK1: genotype effect F(1, 27) = 12.000, *p* = 0.00179 and pERK2/ERK2: genotype effect F(1, 26) = 10.780, *p* = 0.00293]. In the case of MIL, the opposite effect was observed. The drug enhanced the phosphorylation of ERKs in WT mice, but this effect was reduced in D-KO mice lacking the α1D-AR receptor (pERK1/ERK1: D-KO-genotype × MIL interaction effect F(1, 24) = 4.6700, *p* = 0.04089; pERK2/ERK2: D-KO-genotype × MIL interaction effect F(1, 24) = 6.6086, *p* = 0.01678) (Figure 6K,M).

**Figure 7:**
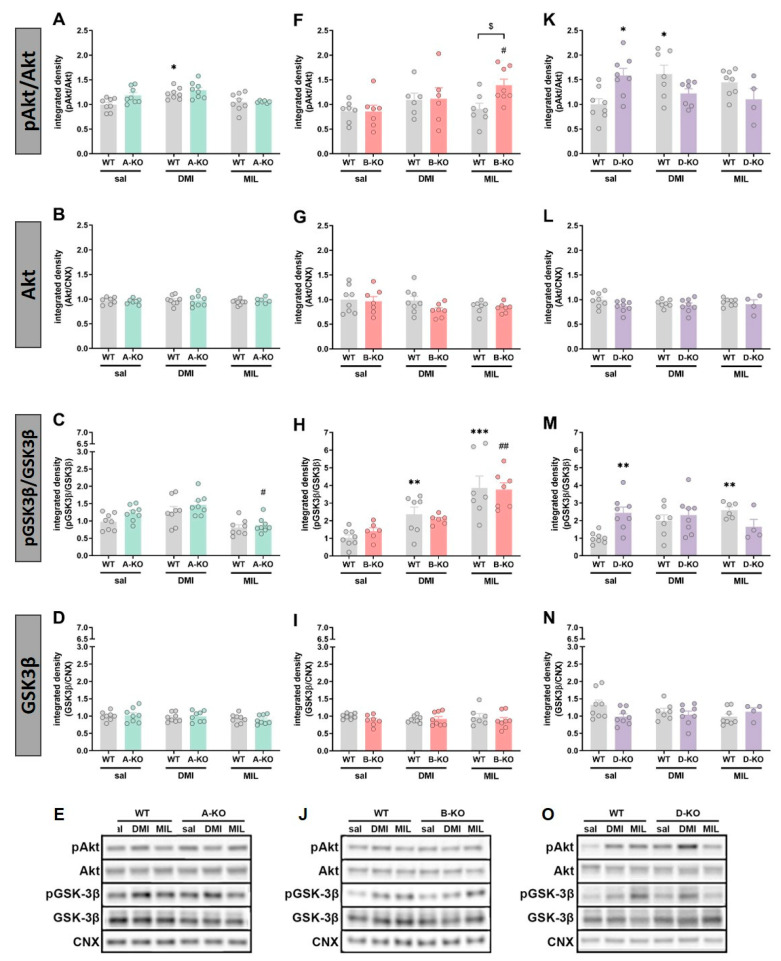
Impact of repeatedly given antidepressant drugs, desipramine (DMI) or milnacipran (MIL), and the mutation effect on phosphorylation and protein levels of Akt and GSK3β in the hippocampus of male wild-type (WT) mice and knockout mice with selective inactivation of α1A-adrenoceptor (A-KO) (graphs (**A**–**E**)), α1B-adrenoceptor (B-KO) (graphs (**F**–**J**)), and α1D-adrenoceptor (D-KO) (graphs (**K**–**O**)), are presented. The mutual representative blot for CNX is visualized in panels (**E**,**J**,**O**), in both Figure 6 and Figure 7. Data are shown as mean values ± SEM with *n* = 4–8, analyzed using two-way analysis of variance (ANOVA) and Tukey’s post hoc test for unequal group sizes (Unequal N HSD).* *p* < 0.05 vs. WT sal, ** *p* < 0.01 vs. WT sal, *** *p* < 0.001 vs. WT sal, ^#^ *p* < 0.05 vs. KO sal, ^##^ *p* < 0.01 vs. KO sal, ^$^ *p* < 0.05 vs. WT drug. Please refer to Figure 6 for the remaining descriptions.

In male mice, selective inactivation of α1A-AR alone did not affect the expression and phosphorylation levels of Akt and GSK3β compared to WT animals, and multiple administrations of antidepressant drugs did not result in changes in the examined proteins (Figure 7A–E). Similarly, selective deletion of α1B-AR alone did not influence the expression levels of Akt and GSK3β proteins (Figure 7F–J); however, chronic administration of MIL increased the phosphorylation of Akt protein (manifested as an increased ratio of pAkt/Akt) in B-KO compared to WT mice [pAkt/Akt: B-KO-genotype × MIL interaction effect F(1, 25) = 4.5793, *p* = 0.04231] (Figure 7F). The GSK3β activity (pGSK3β/GSK3β ratio) was similarly enhanced by DMI and MIL administration, and the drugs’ effects were unchanged by B-KO mutation (Figure 7H). Selective inactivation of α1D-AR alone did not affect Akt or GSK3β expression levels compared to WT animals, which also remained unchanged following chronic administrations of DMI or MIL (Figure 7L,N). However, α1D-AR inactivation influenced the phosphorylation of Akt and GSK3β proteins by increasing the ratio of phosphorylated form to total form compared to the ratio observed in WT animals. Importantly, for both proteins, chronic administrations of MIL resulted in decreased phosphorylation levels in mutants compared to WT animals (pAkt/Akt: D-KO-genotype × MIL interaction effect F(1, 24) = 11.309, *p* = 0.00258; p/tGSK3β: D-KO-genotype × MIL interaction effect F(1, 22) = 18.292, *p* = 0.00031) (Figure 7K,M). On the other hand, repetitive administrations of DMI led to decreased phosphorylation levels only for Akt protein in D-KO mutants compared to WT controls (D-KO-genotype × DMI interaction effect F(1, 26) = 12.740, *p* = 0.00142) (Figure 7K).

**Figure 8:**
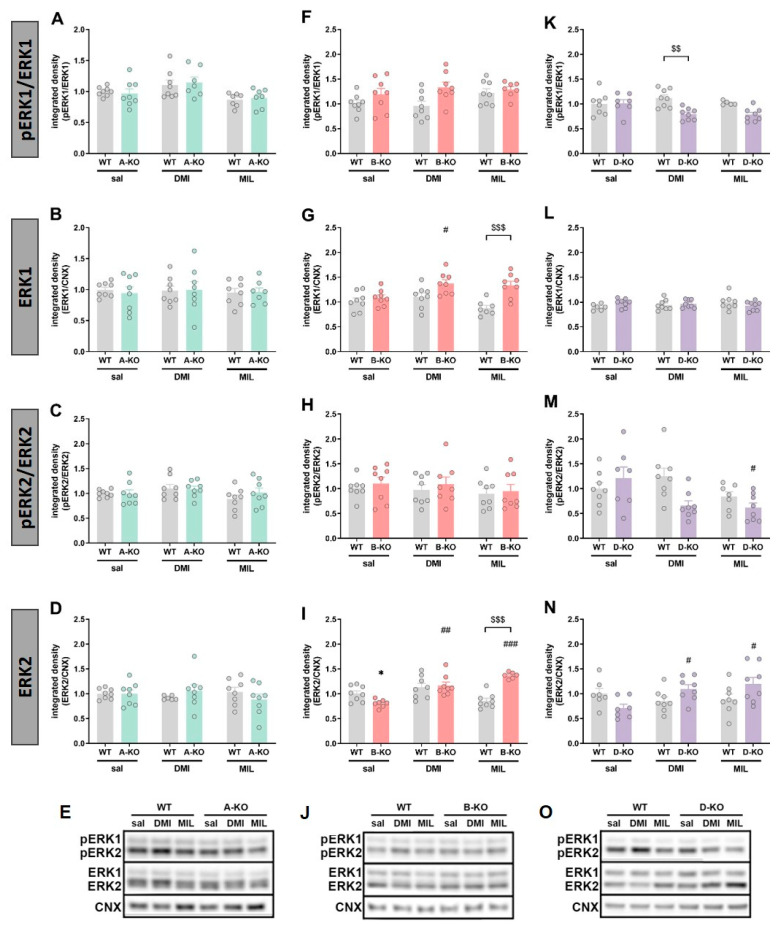
Phosphorylation and protein levels of ERK1 and ERK2 in the hippocampus of female wild-type (WT) mice and knockout mice with selective inactivation of α1A-adrenoceptor (A-KO) (graphs (**A**–**E**)), α1B-adrenoceptor (B-KO) (graphs (**F**–**J**)), and α1D-adrenoceptor (D-KO) (graphs (**K**–**O**)), and after chronic treatment with desipramine (DMI) or milnacipran (MIL). The mutual representative blot for CNX is visualized in panels (**E**,**J**,**O**) in both Figure 8 and Figure 9. Data are shown as mean values ± SEM with *n* = 4–8, analyzed using two-way analysis of variance (ANOVA) and Tukey’s post hoc test for unequal group sizes (Unequal N HSD). * *p* < 0.05 vs. WT sal, ^#^ *p* < 0.05 vs. KO sal, ^##^ *p* < 0.01 vs. KO sal, ^###^ *p* < 0.001 vs. KO sal, ^$$^ *p* < 0.01 vs. WT drug, ^$$$^ *p* < 0.001 vs. WT drug. Please refer to Figure 6 for the remaining descriptions.

Analysis of the results in female mice showed that selective inactivation of α1A-AR did not affect the expression or phosphorylation levels of ERK1 and ERK2 proteins in the hippocampus compared to WT animals, and this lack of effect persisted even after multiple doses of DMI or MIL (Figure 8). Selective inactivation of α1B-AR alone did not alter the phosphorylation of ERK1 and ERK2 proteins, but it did impact the levels of their total forms. Specifically, the mutation caused a decrease in ERK2 expression compared to the WT group, and this effect was mitigated by repeated DMI administration and reversed by multiple doses of MIL [B-KO-genotype × MIL interaction effect F(1, 25) = 58.117, *p* = 0.00000] (Figure 8I). A similar effect was observed in the expression level of the total form of ERK1 protein, where an increase in its level was also noted in α1B-AR knockout mice after chronic administrations of MIL [B-KO-genotype × MIL interaction effect F(1, 27) = 7.9829, *p* = 0.00877] (Figure 8G). In D-KO mutants, a decrease in ERK2 expression compared to WT animals was observed, and chronic administrations of DMI or MIL reversed this effect [DMI: D-KO genotype × DMI interaction effect F(1, 26) = 9.6884, *p* = 0.00447; MIL: D-KO genotype × MIL interaction effect F(1, 26) = 7.6880, *p* = 0.01014] (Figure 8N). On the other hand, selective inactivation of α1D-AR altered the phosphorylation of ERK1 and ERK2. A decrease in the ratio of phosphorylated form to total form was observed in D-KO mice following chronic administrations of DMI (pERK1/ERK1: genotype effect F(1, 27) = 6.4779, *p* = 0.01695; pERK2/ERK2: genotype effect F(1, 27) = 7.1601, *p* = 0.01273) (Figure 8K,M). However, the observed drug-induced changes in the pERK2/ERK2 ratio in D-KO mutants should be interpreted cautiously, as both treatments altered total ERK2 protein levels, and the decrease in ERK2 phosphorylation might be due to the increase in total ERK2 protein levels.

**Figure 9:**
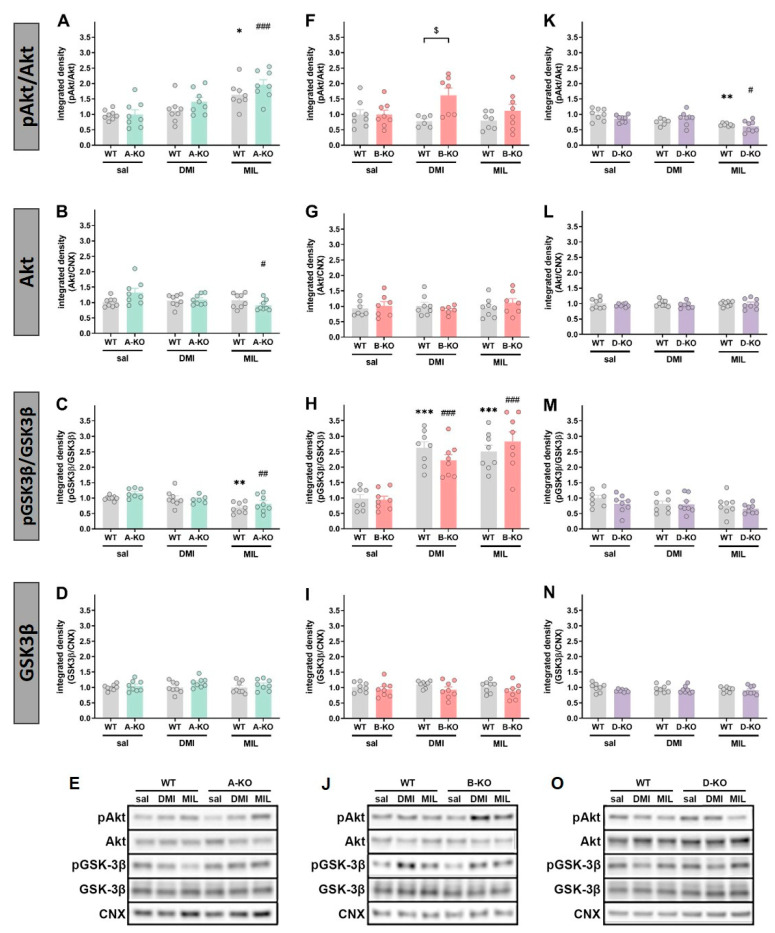
Phosphorylation and protein levels of Akt and GSK3β in the hippocampus of female wild-type (WT) mice and knockout mice with selective inactivation of α1A-adrenoceptor (A-KO) (graphs (**A**–**E**)), α1B-adrenoceptor (B-KO) (graphs (**F**–**J**)), and α1D-adrenoceptor (D-KO) (graphs (**K**–**O**)), and after chronic treatment with DMI or MIL. The mutual representative blot for CNX is visualized in panels (**E**,**J**,**O**), in both Figure 8 and Figure 9. Data are presented as mean values ± SEM, with *n* = 4–8, and analyzed using two-way analysis of variance (ANOVA) followed by Tukey’s post hoc test for unequal group sizes (Unequal N HSD). * *p* < 0.05 vs. WT sal, ** *p* < 0.01 vs. WT sal, *** *p* < 0.001 vs. WT sal, ^#^ *p* < 0.05 vs. KO sal, ^##^ *p* < 0.01 vs. KO sal, ^###^ *p* < 0.001 vs. KO sal, ^$^ *p* < 0.05 vs. WT drug. Please refer to Figure 6 for the remaining descriptions.

In A-KO female mice, no changes were observed in the expression level of GSK3β protein (Figure 9D); however, a decrease in Akt protein level was noted in mutants after chronic administration of MIL [A-KO-genotype × MIL interaction effect F(1, 28) = 6.9259, *p* = 0.01366] (Figure 9B). The phosphorylation of Akt and GSK3β (assessed as their pAkt/Akt and pGSK3β/GSK3β ratios, respectively) in A-KO mice was affected by MIL administration but in opposite ways (Figure 9A,C). Whereas MIL increased the pAkt/Akt ratio similarly in WT and A-KO mice, the drug decreased the pGSK3/GSK3 ratio in these animals [for pAkt/Akt: MIL effect F(1, 28) = 34.955, *p* = 0.00001; for pGSK3β/GSK3β: MIL effect F = (1, 27) = 25.044, *p* = 0.00003 and A-KO-genotype effect: F(1, 27) = 4.9159, *p* = 0.03522]. Moreover, for the pAkt/Akt ratio, two-way ANOVA also revealed an effect of DMI [F(1, 28) = 4.5705, *p* = 0.0414].

The selective inactivation of α1B-AR did not alter the expression levels of Akt and GSK3β proteins or the phosphorylation of GSK3β compared to WT animals, and these proteins remained unchanged following chronic administrations of DMI or MIL (Figure 9G,I). However, it should be noted that both drugs increased the phosphorylation of GSK3β (pGSK3β/GSK3β ratio) in the WT group and in animals with the B-KO mutation compared with the saline-injected groups (Figure 9H). Furthermore, for the Akt protein, it was observed that chronic administrations of DMI increased the ratio of phosphorylated form to total form (pAkt/Akt) in B-KO mice compared to the WT group [B-KO-genotype × DMI interaction effect: F(1, 25) = 6.2888, *p* = 0.01901] (Figure 9F).

The inactivation of α1D-AR in female mice did not affect the expression levels of Akt and GSK3β proteins or the phosphorylation of GSK3β compared to WT animals, and this remained unchanged following chronic administrations of DMI or MIL (Figure 9L–N). In this case, the only change observed was a decrease in the pAkt/Akt ratio caused by MIL treatment, which was similarly affected in both the D-KO and WT groups (Figure 9K) [MIL effect: F(1, 27) = 30.149, *p* = 0.00001].

## 3. Discussion

Although research on α1-AR subtypes has been ongoing for over three decades and has yielded many interesting insights, the detailed functions regulated by individual subtypes in the brain are still not fully understood. However, it has been suggested that in cases of depression and post-traumatic stress disorder, the α1-AR receptor system in the brain may be altered [7,32,49,50]. Many essential processes are controlled by activating the α1-ARs, including behavioral responses [51]. These receptors are engaged in neuroplasticity processes, long-term potentiation (LTP), and memory-related phenomena. In vivo studies on genetic models have revealed diverse involvement of α1-AR subtypes in cognitive processes (reviewed in [7,8]). In transgenic mice of the CAM (constitutively active mutant) type exhibiting systemic overexpression of the constitutively active α1A subtype (α1A-CAM), significantly better performance was observed in behavioral tests assessing learning and spatial memory compared to WT mice, and such mutants exhibited antidepressant and anxiolytic phenotypes [52,53]. Additionally, an enhancement of long-term potentiation (LTP) in the hippocampus was observed in these mice—a brain structure involved in the regulation of neurogenesis and characterized by high α1A-AR expression [53]. Thus, the abovementioned works of Doze and colleagues [53] indicated that the constitutively active α1A subtype of adrenergic receptors plays a positive role in the brain, improving synaptic plasticity, cognitive function, mood and longevity. Changes in cognitive function were also observed in α1A-adrenergic receptor knockout mice. These mice showed reduced cognitive function, which correlates with the results of the studies mentioned above [reviewed in [54].

In mice with systemic overexpression of the constitutively active α1B subtype (α1B-CAM), distinct effects were observed. The dominant phenotype in these mice was a neurodegeneration that resembles a parkinsonian-like syndrome called multiple system atrophy (MSA) particularly in terms of autonomic dysfunction and decreased serum levels of catecholamines and cortisol [55]. Subsequent studies in this model provided evidence that this over-activity of the α1B-AR is sufficient to cause a parkinsonian-like synucleinopathy with excessive tyrosine-nitration of the α-synuclein [56]. Such a post-translational modification of α-synuclein can promote the formation of intracytoplasmic inclusions, a hallmark of Parkinson’s disease and other synucleinopathies [57]. On the other hand, studies with α1B-AR knockout mice showed that this receptor subtype is involved in memory consolidation and fear-motivated exploratory activity [58,59,60].

Our previous studies indicated that the α1A and α1B receptor subtypes are differentially involved in antidepressant action. Thus, we have shown that chronic imipramine or electroconvulsive shock therapy increased the expression of mRNA encoding α1A-ARs, but not α1B-ARs, in the cerebral cortex of rats, suggesting the importance of α1A-ARs in mechanism of action of these two therapies [33]. Moreover, we demonstrated that the noradrenergic component of the action of these antidepressant agents plays an essential role in the modulation of α1A-ARs in the rat cerebral cortex. Previous noradrenergic depletion with DSP-4 (a neurotoxin selective for the noradrenergic nerve terminals) significantly attenuated the effect of imipramine and abolished the effect of electroconvulsive shock [61]. The subsequent finding by Doze et al. [52], based on the α1A-CAM model and showing that α1A signaling promotes antidepressant-like behavior in the tail suspension test and the forced swimming test, suggested that increased α1A-AR expression after chronic administration of norepinephrine-linked antidepressants or electroconvulsive shocks, as observed in our studies, may play an essential role in mediating the antidepressant effects of these treatments.

Of the three α1-AR receptor subtypes, the α1D-AR is the least studied with respect to the mechanism of action of antidepressants. The role of the α1D subtype in the central nervous system (CNS) was primarily studied in α1D receptor knockout mice, with available data focusing on their phenotypic and behavioral traits. This was reported, among other things, that knockout mice lacking α1D-AR expression (α1D-KO) show limitations in exploratory behaviors in the novel environment test [26]. The α1D receptor appears to mediate behavioral activation, and existing literature suggests its involvement in working memory, attention, and motor coordination processes [62]. The role of α1-AR subtypes, including the α1D-AR subtype, in the antidepressant-like effects of imipramine was investigated by Ribeiro and Pupo [63]. The study assessing the involvement of three receptor subtypes in the anti-immobility effect of imipramine in the mouse tail suspension test identified the α1B-subtype as the main player in imipramine’s mechanism of action. This conclusion was based on the observation that only α1B-AR antagonist significantly antagonized the anti-immobility effect of imipramine while other two antagonists were ineffective. In this study, however, selective antagonism of the α1A- and α1D-AR subtypes alone resulted in antidepressant-like effects, suggesting functional diversity among the three α1-AR subtypes. Moreover, it is worth mentioning that the results of in vitro studies suggested that the α1B receptor may be most strongly activated by the increased availability of noradrenaline as a result of the action of imipramine and other TCAs [64,65].

Our current study, which investigates the functional involvement of α1-ARs in the effects induced by antidepressant drugs, DMI and MIL, takes an innovative approach. We utilized three transgenic mouse lines with selective knockout of individual α1-AR subtypes: α1A (A-KO), α1B (B-KO), and α1D (D-KO). Before this, we confirmed the efficacy of the mutations using mice with a triple knockout of all three α1-AR subtypes (ABD-KO) via autoradiography. This thorough approach ensured the accuracy and reliability of our results. In the analyzed brain structures, no specific radioligand binding signal to α1-ARs was observed, confirming the effectiveness of the ABD-KO mutation. Notably, the inactivation of α1-ARs did not affect the density of the remaining adrenergic receptor types, α2-AR and β-AR, which could have impacted the results of the planned assays for subsequent stages of the experiment had such an effect occurred.

Next, we investigated the impact of A-KO, B-KO, and D-KO mutations on the hypothalamic tissue levels of selected neurotransmitters and their metabolites. Our study showed that the D-KO mutation significantly lowered noradrenaline levels compared with the B-KO subtype. However, this decrease did not reach statistical significance relative to the WT control. Despite this, the impact of D-KO mutation suggests a potentially important role of the α1D-AR subtype in noradrenergic transmission in the HY. Furthermore, it may indicate a failure of adaptive mechanisms in the absence of functional α1D-AR, possibly related to its unique constitutive activity [8,66]. Interestingly, Konstandi et al. [67] demonstrated an increase in hypothalamic noradrenaline levels of mice under physical stress. One can speculate that the D-KO mutation could affect the function of the hypothalamic–pituitary–adrenal (HPA) axis and reduce animals’ susceptibility to stress.

In the second part of our research, we explored the involvement of α1-ARs in the molecular effects of two antidepressant drugs, DMI and MIL. This study is significant because it provides insight into the complex molecular mechanisms behind how these drugs work. DMI, a secondary amine and a systemic metabolite of imipramine, shows much more potent inhibition of noradrenaline reuptake than serotonin, to the point that it can be classified as an NRI (noradrenaline reuptake inhibitor) [40]. The second drug, MIL, belongs to the SNRI (serotonin-noradrenaline reuptake inhibitor) group. MIL inhibits the reuptake of both norepinephrine and serotonin; however, unlike TCAs, such as DMI, MIL has weak receptor-binding activity [41]. However, it is essential to note that the antidepressants used here served as pharmacological tools for inducing biochemical changes in α1-AR subtypes and related signaling pathways. Therefore, they are not necessarily the same as the antidepressants typically used in clinical settings. Moreover, it should be emphasized that molecular effects observed in in vitro and animal research may not fully reflect clinical pharmacology, especially with differences in dosages and treatment durations.

Additionally, we chose antidepressants with a noradrenergic component because α1-AR subtypes might be differentially regulated by such components, as suggested by our previous research [61,64]. Moreover, the molecular changes observed after chronic antidepressant administration can be considered adaptive responses to the drug’s effects, which are not directly related to the drug’s presence in the brain. Pharmacokinetic studies show that, 24 h after the last dose, when mice were sacrificed and tissue samples were collected, the levels of DMI and MIL in the brain are minimal [68,69,70]. In such a situation, the antagonistic effect of DMI on the cerebral α1-ARs may also be minimal. Additionally, GPCRs (including α1-ARs) undergo desensitization after prolonged exposure to an agonist (or a drug), which affects their response to subsequent agonists and modulates the intracellular signaling pathways downstream of the receptor. Our recent in vitro studies demonstrated that the reactiveness of α1B- and α1D-ARs to noradrenaline (measured as second-messenger accumulation) increased after 120 h of incubation with DMI [57]. Therefore, the α1-AR receptor exposed to prolonged DMI seems to become unresponsive to the drug’s antagonistic effects.

We assessed the mRNA expression levels of genes encoding for individual α1-ARs’subtypes in the prefrontal cortex of A-KO, B-KO, D-KO, and WT mice. We found that the knockout of α1A or α1B subtypes did not alter *ADRA1D* mRNA expression. However, the knockout of the α1D subtype led to a statistically significant increase in *ADRA1A* gene expression and a tendency toward upregulation of the *ADRA1B* subtype (in saline-injected D-KO mice); both DMI and MIL treatments reversed these changes. These findings strongly support our hypothesis that the α1D-AR subtype may play a particular role in the effects of the investigated antidepressant drugs. Though the *ADRA1D* gene mRNA expression, by itself, was not affected by repeated antidepressant drug administrations, an intact α1D-AR was necessary for DMI and MIL-induced changes in the expression of genes encoding the remaining subtypes of the α1-ARs, at least in the PFC. In other words, while the *ADRA1D* gene mRNA expression is not a direct transcriptional target for DMI or MIL, it plays a modulatory or permissive role. Its presence is essential for certain molecular effects of the drugs’ actions to occur fully. Furthermore, we observed that α1B knockout prevents the DMI-induced increase in *ADRA1A* mRNA observed in WT animals. This shows the importance of α1B for DMI action, consistent with our earlier in vitro studies and highlighting the significant role of the α1B subtype in this drug’s action [64]. However, at this stage of research, conclusions should be drawn with caution, as changes in mRNA expression do not always translate into changes in protein levels. Therefore, the functional significance of the observed changes requires confirmation by other methods.

In the next step, gene expression profiling was performed in the hippocampus of A-KO, B-KO, and D-KO mice. The mutation and repeated administration of antidepressant drugs moderately altered the gene expression profile, as statistically significant changes were observed in a relatively small number of transcripts. Here, a potential methodological limitation should be noted. A relatively small number of changed transcripts in microarray data could also be a result of the method’s low sensitivity and high biological variability, combined with relatively small sample sizes.

Furthermore, none of these transcripts were regulated in all three transgenic lines, and only a few were regulated in two. In addition to B-KO mice showing a distinct gene expression profile compared to A-KO and D-KO mice, this finding further supports the functional diversity of α1-AR subtypes.

Even if the co-regulated transcripts are few, it is important to highlight the role some of them play in cellular function. For instance, the *Hpcal1* gene, which encode Hippocalcin-like protein 1 (HPCAL1), was up-regulated in both A-KO and B-KO. HPCAL1 belongs to the neuron-specific calcium-binding proteins. It functions as a neuronal calcium sensor with roles in both normal brain and eye function, and in the suppression of certain cancers [71]. Furthermore, *Gnb2l1* (RACK1), under-regulated in A-KO and D-KO, encodes for Receptor for Activated C Kinase 1 (RACK1), which functions as a scaffolding protein that participates in the recruitment, assembly, and regulation of various signaling molecules. It interacts with many proteins and is essential for several cellular processes, stabilizing activated PKC and enhancing PKC-mediated phosphorylation [72,73]. The Pla2g4e, which was co-regulated in B-KO and D-KO, encodes a member of the cytosolic phospholipase A2 group IV family. Members of this group are involved in regulating membrane tubule-mediated transport. The enzyme encoded by this gene plays a role in trafficking via the clathrin-independent endocytic pathway [74,75]. Note that the genes mentioned above encode proteins that can functionally interact with the α1-AR signaling pathway. Since activation of the α1-AR leads to an increase in intracellular Ca^2+^, these receptors will, at least in part, influence the function or expression of intracellular calcium-binding proteins or calcium-dependent enzymes. Thus, RACK1 interacts, among others, with PKC, a signaling molecule downstream of the α1-AR. The PLA2 (Group IVA PLA2) is an enzyme that is responsible for the hydrolysis of membrane phospholipids such as phosphatidylcholine. Furthermore, this enzyme has a high affinity and specificity for phosphatidylinositol 4,5-bisphosphate (PIP2), the molecular substrate targeted by PLC following the α1-ARs activation to produce second messengers, IP3 and DAG [74]. Moreover, it has been reported that stimulation of the α1B and α1D-AR can activate phospholipase A2 in an in vitro cellular model (reviewed in [13,14]). Thus, the above findings further support the functional diversity of α1-AR subtypes in regulating cellular processes. On the other hand, the data suggest that key intracellular proteins, such as those mentioned above, can be controlled by at least two α1-AR subtypes.

It is also worth noting that the chronic administration of DMI and MIL (to WT mice) resulted in co-regulation, mainly of transcripts encoding small nucleolar RNA and centrosomal protein 83. Small nucleolar RNAs (snoRNAs) constitute a family of short non-protein-coding RNAs (ncRNAs) enriched in the nucleolus and best known for guiding posttranscriptional modifications on ribosomal (rRNAs) and small nuclear RNAs (snRNAs) [76]. In turn, the protein encoded by Cep83 is a centriolar protein involved in primary cilium assembly. Defects in this gene have been associated with infantile nephronophthisis and intellectual disability [77,78]. Thus, we have demonstrated that DMI and MIL, antidepressants from different classes that differ in their primary pharmacological mechanisms of action, can regulate the same intracellular factors involved in post-translational modifications.

An exciting result was achieved by analyzing the effects of repeated MIL administration. Ontological analysis of transcripts significantly regulated in the hippocampus due to MIL treatment revealed their involvement in processes such as ceramide biosynthesis, oligodendrocyte differentiation, and glycolipid metabolism. These processes are closely interconnected, as ceramides mediate the organization of lipid rafts in the cell membrane, which include glycolipids involved in oligodendrocyte differentiation. In turn, mature oligodendrocytes in the central nervous system produce the myelin sheath around neuronal axons, and lipid rafts mediate the interaction between myelin-associated glycoprotein (MAG) and its receptor on neurons. In neurons, lipid rafts play a crucial role in modulating ion channels and neurotransmitter receptors, as well as in neurotransmitter release via exocytosis [79]. The involvement of the sphingomyelin signaling pathway, in which DAG mediates stimulation of the hydrolysis of sphingomyelin into ceramide, has been repeatedly demonstrated in the action of antidepressant drugs [80,81,82,83,84].

Nevertheless, the results obtained in the current study add to this knowledge by highlighting another drug, MIL. Furthermore, based on analysis of gene expression patterns in transcripts significantly regulated in the hippocampi of A-KO, B-KO, and D-KO mice after repeated MIL administration, we found that deleting α1D-AR abolished the drug-induced changes in transcript expression, further emphasizing the crucial role of the α1D-AR subtype in MIL’s action. This finding suggests that the intact α1D-AR is essential for MIL’s molecular effects. Of course, if this impact is meaningful, further study would be required on selected genes involved, i.e., in definite intracellular signaling pathways.

The following experiment also showed the involvement of the α1D subtype in the action of MIL. In it, we assessed the level of expression and phosphorylation of selected protein kinases in the same brain structure of the same subjects, as described above. These were GSK3β, Akt, and Erk1/2—the proteins that are components of intracellular signaling pathways implicated in mood disorders. Particularly noteworthy were our results concerning the GSK3β.

The protein kinase GSK3β has been extensively studied in the context of depression, with recent reports suggesting it plays an essential role in the etiology and pharmacotherapy of this disorder. GSK3 is involved in many cellular signaling pathways, and other kinases, such as PI3K, Akt, ERK1/2, PKC, and PKA, can regulate its activity. As a result, many crucial intracellular signaling pathways converge on GSK3, including those triggered by various growth factors that activate sequentially the phosphoinositide 3-kinase (PI3K) and Akt, as well as pathways involving PKA and PKC. The primary physiological mechanism controlling GSK3 activity is the phosphorylation of an N-terminal serine residue (Ser9-GSK3β, the dominant brain isoform, or Ser21-GSK3α). GSK3β is a constantly active enzyme, and this serine phosphorylation inhibits its activity. Abnormally active GSK3 has been linked to a wide variety of CNS diseases, including mood disorders, schizophrenia, and some conditions involving cognitive impairment (reviewed in [85,86]).

Furthermore, over the past few years, it has been repeatedly shown that both chronic stress and antidepressant drugs affect GSK3β activity in the hippocampus. Chronic administration of antidepressant medications, e.g., citalopram, fluoxetine, venlafaxine, and lithium, as well, was demonstrated to cause an increase in GSK-3β phosphorylation that results in inhibiting the enzyme activity. Inhibition of GSK3 is also necessary for the rapid antidepressant effect of ketamine in mice [87,88,89,90]. Moreover, silencing GSK3β expression in the hippocampus was shown to be sufficient to induce an antidepressant effect in behavioral tests on mice subjected to chronic stress [91]. Thus, inhibition of GSK3β, achieved either directly by enzyme silencing or indirectly by pharmacological tools that enhance phosphorylation (leading to subsequent inhibition of GSK3β activity), can produce antidepressant-like effects in rodents.

There are clinical findings indicating changes also in the ERK signaling pathway in major depression. Post-mortem studies showed decreased Raf-ERK1/2 signaling in the prefrontal cortex and hippocampus in depressed suicide subjects [37]. Other findings suggested a correlation between decreased Akt activity and decreased ERK activity in depression (reviewed in [92]). Taken together, these signaling studies implicated the disruption of positive plasticity in depression and indicated that all three kinases analyzed in our current study are involved in this process.

Our study in male hippocampi demonstrated that knockout of α1D-AR increased GSK3β phosphorylation (pGSK3β/GSK3β ratio) and, consequently, its expected functional inhibition, as well as enhanced Akt phosphorylation (pAkt/Akt ratio), consistent with Akt activation. A rise in the phosphorylation of ERK1 and ERK2, and thus their activation, was also noted, although this increase was much weaker than in the case of the other two kinases. Moreover, we found that chronic DMI and MIL increased phosphorylation of GSK3β, Akt, and ERK1 in WT mice. Our results are therefore consistent with the above-mentioned papers by other authors, showing an increase in phosphorylation of three kinases under the action of antidepressants, which in the case of GSK3β results in the inhibition of kinase activity. The inhibition of GSK3 activity and activation of Akt and ERK after treatment with various antidepressants have been reported frequently (reviewed in [92]). However, to the best of our knowledge, it is shown for the first time here that chronic administration of MIL elevates the phosphorylation of GSK3β and Akt kinases.

Furthermore, our results show that α1D-AR inactivation attenuated the MIL-induced increase in hippocampal GSK3β and Akt phosphorylation, consistent with gene expression profiling results (this study) showing the importance of an intact α1D-AR for MIL effects. Since the GSK3β inhibitory phosphorylation on Ser is necessary for the antidepressant effect of some drugs [89], and evidence exists that α1A-AR action may regulate GSK3β, increasing its phosphorylation thus inhibiting the kinase activity [93], one can hypothesize that, in this case, the α1D- and α1A-AR subtypes can compensate for each other’s function depending on the antidepressant drug used. This hypothesis is based partly on our findings from this study. We found that knocking out the α1D subtype led to a statistically significant increase in *ADRA1A* mRNA expression and a tendency toward upregulation of the *ADRA1B* subtype (though it has not been verified whether these mRNA changes are translated into functional receptor protein). Thus, in the case of D-KO, both the α1A- and α1B-AR subtypes are left untouched and functionally active. It is tempting to speculate that at least α1A-AR might compensate for the lack of α1D-AR and increase phosphorylation of GSK3β and Akt in D-KO, as observed in this study in males. Especially since, as mentioned above, the α1A receptor is involved in regulating GSK3β. Such compensation was no longer possible in the case of MIL, whose effect on GSK3 and Akt phosphorylation depended on functional α1D-AR. In this case, one can assume that the positive effect of MIL on cell signaling will be diminished by α1D-AR deletion, which could be relevant and lead to a reduction in the antidepressant effects of MIL. However, the “compensation hypothesis” is partly based on premises derived from cell line studies and recombinant systems, with no confirmation yet that they occur under physiological conditions in vivo. Thus, though it is an attractive hypothesis, great caution should be exercised when considering it in the context of in vivo processes.

The results obtained in the current study also highlight the differences between α1-AR subtypes, as the deletion of α1A-AR or α1B-AR, unlike α1D-AR, did not cause changes in the activity (phosphorylation) of the proteins as mentioned earlier. The effect of mutations on MIL-induced changes in the phosphorylation of the tested kinases was also observed in B-KO animals. Still, they differed from those in D-KO animals, manifesting as increased Akt activation and ERK1 expression.

Moreover, these findings emphasize an essential methodological aspect, increasingly discussed in the literature concerning the etiology and pharmacotherapy of mental disorders—namely, sex differences [94,95,96,97,98]. Our study reveals that, in male D-KO mice, most of the observed results attributed the impact of the mutation to the effects of MIL action. In contrast, the impact of the mutation in female D-KO mice was evident mainly in the case of DMI. Furthermore, the profile of changes in protein phosphorylation in females was also different, as inactivation of α1D-AR did not affect GSK3β and Akt phosphorylation ratio but decreased ERK1 and ERK2 activation. Thus, in the hippocampus of female D-KO mice, repeated administration of DMI produced the opposite effect compared to repeated MIL administration in the same structure in male D-KO mice.

Nevertheless, it should be noted that in the case of ERK2, in addition to changes in phosphorylation (pERK2/ERK2 ratio), DMI and MIL also induced alterations in total ERK2 levels (a decrease in WT-MIL, and an increase in DMI D-KO and MIL D-KO). Thus, the DMI- and MIL-induced changes in the pERK2/ERK2 ratio should be interpreted with caution, as both the observed increase in phosphorylation in the WT-MIL (male group) and the decreases in phosphorylation in the female DMI D-KO and MIL D-KO groups are likely due to changes in total ERK2 levels. Furthermore, in the α1B-AR study in female mice, changes in total ERK1/2 were also observed. The decrease in total ERK2 level induced by the α1B-AR deletion was mitigated or reversed by DMI or MIL administration, respectively. Similarly, the total ERK1 level was increased in B-KO mice after MIL administration. Furthermore, in the α1B-AR study involving female mice, changes in the total ERK1/2 levels were also observed. The reduction in total ERK2 caused by the α1B-AR deletion was either lessened or reversed by DMI or MIL administration, respectively. Similarly, the total ERK1 level increased in B-KO mice after MIL administration. Therefore, the interpretation of these ERK1/ERK2 data remains unclear, and discussing their implications for intracellular signaling becomes much more complicated. Interestingly, the increases in total ERK1 and ERK2 levels induced by chronic DMI treatment (current study) are consistent with our previous data on the effects of chronic imipramine administration to rats. Thus, assessing ERK1/2 protein levels and phosphorylation in the rats’ prefrontal cortex, we found that imipramine increased total ERK1 and ERK2 levels [38], a finding corroborated by the current results.

On the other hand, the inconsistent effects of MIL and DMI on ERK phosphorylation may be due to differences in their pharmacological profiles. Though the ERK pathway has been extensively studied in depression (in postmortem studies as well as in animal models and antidepressant context), the findings have not always been consistent (reviewed in [92]). Although both MIL and DMI are antidepressants, they differ somewhat in their pharmacological profile, including affinity for monoamine receptors (very weak or strong) and the strength and type of neurotransmitter reuptake inhibition [69]. Thus, while changes in protein phosphorylation or protein expression illustrate that both antidepressants induce molecular neuroplasticity, these drugs may not exert identical intracellular effects.

The changes in protein kinases’ phosphorylation described above suggest a sex-dependent effect. Although we did not perform statistical analysis of the sex factor, the patterns of changes in pERK1/ERK1, pAkt/Akt, and pGSK3β/GSK3β ratios induced by chronic DMI and MIL administration, particularly in the hippocampus of female and male D-KO mice, show sex-specificity. The observation of a sex-dependent pattern of changes after chronic DMI treatment is novel. It contrasts with previously published studies that focused exclusively on one sex or the effect of a single drug administration [99,100,101]. The only notable exception in the literature is a recent study in the hippocampus of rats, which suggests that different molecular mechanisms may underlie the therapeutic effects of DMI in males and females [102]. As for MIL, the number of reports addressing sex differences in its effects is even smaller [103,104]. One of those clinical studies comparing gender differences in response to milnacipran, fluvoxamine, and paroxetine demonstrated that milnacipran treatment showed a tendency toward a higher rate of improvement in men than in women [104]. Sex differences in animal models have been shown for other drugs in the SNRI class, as well. Using various experimental paradigms, a more effective action of fluoxetine, venlafaxine and duloxetine was observed in males than in females [105,106,107].

The latest research using compounds affecting noradrenergic transmission suggests that α1-AR may be responsible for the sex differences in the noradrenergic modulation of attention and impulsivity [108]. In this context, our study’s findings indicate the promising potential of α1-AR receptor subtypes for translational research aimed at developing new compounds for the treatment of neuropsychiatric disorders and highlight the need to improve sex-specific therapies.

### Limitations

We recognize certain limitations in this study. Only two antidepressants were used as pharmacological tools, which limits the ability to generalize the findings to other antidepressants. Future research should investigate the role of α1-AR receptor subtypes in the intracellular mechanisms of various other antidepressants, including SSRI, noradrenaline reuptake inhibitors (NRI), and monoamine oxidase inhibitors (MAOI) that could modulate α1-ARs activity differently. Moreover, the drugs used in this study, DMI and MIL, belong to two distinct classes: TCAs and SNRIs, respectively. In addition to inhibiting the reuptake of neurotransmitters by both drugs, the former also exerts direct antagonistic effects on various receptors, including α1-ARs, while the latter has minimal affinity for these receptors (Ki > 10,000 nM) [69]. At first glance, this might seem like a poor choice, but that is not the case. Considering that DMI inhibits noradrenaline reuptake more potently than serotonin, and MIL inhibits the reuptake of both neurotransmitters with similar potency, we chose DMI and MIL as representative drugs for different mechanistic approaches. The changes we observed, which were induced by the chronic administration of antidepressants at the molecular level, can be considered adaptive to the drug’s action, though not related to the direct presence of the drug in the brain. In fact, after 24 h from the last drug administration (when the mice were sacrificed and tissue for biochemical research was collected), the concentrations of DMI and MIL in the brain are at a minimal level, as revealed by our and others’ pharmacokinetic studies [68,69,70]. Moreover, based on our previous findings indicating that prolonged exposure to DMI of the receptor makes it insensitive to the drug’s antagonistic effects and more responsive to noradrenaline stimulation (at least in an in vitro model [65]), we hypothesize that a similar process may occur in vivo after repeated DMI administration. This study mainly uses these drugs as tools to modulate noradrenergic (DMI) and combined noradrenergic-serotonin (MIL) transmission. Future research should investigate whether the α1-AR receptor, chronically exposed in vivo to DMI, develops “functional resistance” to the drug’s antagonistic action and whether this phenomenon is subtype-dependent for α1-ARs. However, it should always be considered that direct extrapolation of in vitro results to in vivo studies requires caution, as physiological complexity may alter receptor behavior.

Another limitation is the scarcity of subsequent analyses performed after gene profiling. The heat map shown in this manuscript is a standard way to display expression data, offering a visual of differentially expressed genes. However, it provides only a broad overview and lacks detailed quantitative accuracy. Future studies should include the validation of specific genes using quantitative methods, such as RT-qPCR, which was not possible at this stage. We understand that gene expression profiling offers a snapshot of gene activity, essentially reflecting a cell’s functional state. While these data help make general inferences, as we do in this study, there are limitations. A heat map does not show how individual genes relate to each other; it only displays the overall pattern of significant changes across all transcripts influenced by the drug or mutation. To analyze the expression profiling data regarding the effects of MIL and its potential attenuation by KO of α1D-AR (as shown in the profiling data), we used the DAVID Functional Annotation Bioinformatics Microarray Analysis to further examine the significantly under- and overrepresented genes after MIL treatment. DAVID analysis of transcripts significantly regulated after chronic MIL treatment in WT mice revealed enrichment in 17 KEGG pathways, including those related to ceramide biosynthesis, oligodendrocyte differentiation, and glycolipid metabolism. These pathways are interlinked, as ceramides regulate lipid raft composition, which in turn is crucial for the maturation of oligodendrocytes and the myelination processes. Such changes are biologically relevant because oligodendrocyte function and sphingolipid signaling have been repeatedly implicated in the neuroplastic and antidepressant-related mechanisms. By contrast, in the D-KO MIL-treated mice, DAVID analysis identified only 6 KEGG pathways, with limited overlap with WT (see Supplementary Figure S3). This attenuation indicates that the absence of the α1D receptor substantially reduces MIL’s ability to influence intracellular signaling cascades related to neuroplasticity and cellular metabolism. It might be interpreted not only as a quantitative reduction in the number of regulated transcripts but also as a qualitative shift in the biological processes engaged, highlighting the α1D receptor’s crucial role in mediating MIL’s molecular effects. However, this is very superficial without going more in depth into the mechanisms behind this study. Thus, by applying DAVID functional annotation, we have just demonstrated that MIL-induced transcriptomic regulation in WT mice converges on biologically meaningful pathways. In contrast, these processes are largely absent in D-KO mice, supporting our conclusion that α1D-AR is necessary for the full spectrum of MIL’s molecular actions. Exploring the story further was beyond the scope of this study. However, we believe that our findings provide a solid foundation for future, more detailed studies by researchers interested in this area.

## 4. Materials and Methods

### 4.1. Animals

The study was conducted on male and female knockout mice (KO) targeting α1A (A-KO), α1B (B-KO), or α1D (D-KO) adrenergic receptor subtypes. All three lines were kept in the C57Bl6/J background as they originated when creating the KO (for a complete description of the genetic background, see [109,110,111]). Wild-type (WT) mice were always the littermates of KO animals from breeding each line. Only in the case of the autoradiography analyses of adrenergic receptors, naïve mice (i.e., mice not previously used in behavioral tests or treatments) with knockout of all three α1-AR subtypes (ABD-KO) (a gift from Professors J. C. McGrath and C. J. Daly, University of Glasgow) were used.

### 4.2. Mice Breeding and Genotyping

A-KO, B-KO, and D-KO mice were bred according to standard procedure by crossing heterozygous mice of each line. Genotyping was performed via PCR using sequence-specific primers as follows:

A-KO:

AGCTAACCATTTCAGCAAAGAC

CAAGATCACCCCAAGTAGAATG

TAACCGTGCATCTGCCAGTTTG

B-KO:

ATTTGTCACGTCCTGCACGAC

CCTGCAGGTATGAGGTCTGTG

CCAAAATGCTCCCAACTCTG

D-KO:

CTTTGTTAAGAAGGGTGAGAACAGAG

GCTAGGAAGACACCCACTCC

GACATCCTGAGCGTCACTTTC

A tail biopsy for genotyping was performed between 21 and 30 days of age. Mice were also re-genotyped after completed experiments. DNA digestion and genotyping were performed with a commercially available kit, AccuStart™ II Mouse Genotyping Kit, QuantaBio/VWR, (QIAGEN Beverly Inc., Beverly, MA, USA) according to the manufacturer’s protocol and as described previously [112]. Male and female mice were kept separately with their control (*w*/*t*) littermates of the same sex in self-ventilated cages under standard laboratory conditions (12 h light/dark cycle, food and water ad libitum). The study followed the guidelines in the Guide for the Care and Use of Laboratory Animals of the National Institutes of Health. It fulfilled the requirements of EU Directive 2010/63/EU on the protection of animals used for scientific purposes and ARRIVE guidelines. The protocol for all behavioral studies, tail biopsy, and mouse sacrifice was approved by the Local Ethical Commission for Animal Experiments at the Maj Institute of Pharmacology, Polish Academy of Sciences (Permit Number: 1233/2015 dated 25 June 2015). The GMO animal colony was maintained with the permission of the Polish Ministry of Environment (Permit Numbers: 59/2013, dated 17 April 2013, and 161/2018, dated 21 December 2018).

### 4.3. Mice and Drugs Administration

The mice were about 9 weeks old and weighed between 25–27 g for males and 20–21 g for females at the start of drug administration. They were housed in groups of 7–8 with unlimited access to commercial food and tap water in standard mouse cages; the cage dimensions were 265 mm width × 180 mm height × 420 mm depth, and the floor area was 825 square cm. The following standard laboratory conditions were maintained in the animal room: an artificial 12-h light/dark cycle (lights on from 7 a.m. to 7 p.m.), a constant temperature of 22 °C ± 2 °C, and a relative humidity of 45–55%. Before the onset of the experiment, the animals were allowed a 1-week habituation period.

Desipramine hydrochloride (DMI, 20 mg/kg, i.p., Sigma-Aldrich, Darmstadt, Germany) and Milnacipran hydrochloride (MIL, 30 mg/kg, i.p., Biosynth Carbosynth, Compton, Berkshire, UK) were injected once daily for 21 consecutive days. Control mice received vehicle injections, precisely a solution of 0.9% NaCl (saline) (POLPHARMA S.A. Starogard Gdański, Poland). The doses of the drugs used and the treatment schedule were selected based on previous pharmacokinetic studies, our own, and those described by other researchers [68,69,70].

The mice were decapitated at around 12 weeks of age during the light phase of the cycle (between 9:00 a.m. and 11 a.m.), 24 h after the last drug injection. The brain structures were dissected and immediately flash-frozen in liquid nitrogen and stored at −80 °C for further study. For sample collection and storage for mRNA assays, see Section 4.6.

### 4.4. Autoradiography of Adrenergic Receptors in the Brain of WT and ABD-KO Mice

Quantitative autoradiography was carried out as described by [45,47,48]. The male ABD-KO (triple KO) and control WT mice were decapitated, and their brains were removed and stored at −80 °C until subsequent processing. Frozen brains were cut in the coronal plane at −21 °C, using a LEICA Jung CM 3000 cryostat (Leica, Leitz-Park, Wetzlar, Germany), into 12 μm-thick sections. Tissue sections at bregma + 1.70 ± 0.1 mm and −1.34 ± 0.1 mm, according to Paxinos and Franklin [113], were selected for analysis. The sections were thaw-mounted on gelatin-coated glass microscope slides. They were then stored at −80 °C. Immediately prior to use, the slide-mounted sections were dried at room temperature.

#### 4.4.1. [^3^H]Prazosin Binding to α1-AR

For α1-ARs binding, slide-mounted sections were thawed and preincubated for 1 h at room temperature in a Krebs-modified buffer (KRBM) pH 7.8, containing 10 mM Na_2_HPO_4_, 119 mM NaCl, 6 mM KCl, 1.2 mM MgSO_4_, and 1.3 mM CaCl_2_. For the α1-adrenoceptor assay, sections were further incubated for 1 h in the same buffer containing 0.9 nM [^3^H]prazosin (Perkin Elmer, Waltham, MA, USA; specific activity 85 Ci/mmol). Adjacent sections were incubated with radioligand plus 10 µM WB4101 (Sigma-Aldrich, Darmstadt, Germany) to determine nonspecific binding. Following incubation, sections were rinsed twice for 3 sec and four times for 10 min with ice-cold KRBM and briefly immersed into ice-cold water. The preparations were then dried in a stream of cold air.

#### 4.4.2. [^3^H]RX821002 Binding to α2-ARs

For α2-ARs binding, the slides were pre-incubated (15 min/RT) in 50 mM phosphate buffer, pH 7.4. Radioligand binding was performed using the same buffer with [^3^H]RX821002 (specific activity: 63.8 Ci/mmol; NEN Life Science Products, Boston, MA, USA) at a concentration of 0.5 nM and the incubation lasted for 60 min. Non-specific binding was determined in the presence of 5 µM RX 821,002 (Sigma-Aldrich, Darmstadt, Germany). Sections were rinsed twice with ice-cold buffer and once with deionized water at 4 °C, each lasting 1 min. The preparations were then dried in a stream of cold air.

#### 4.4.3. [^3^H]CGP121 Binding to β-ARs

For β-ARs binding tissue sections were pre-incubated (15 min/RT) in 50 mM Tris-HCl buffer (pH 7.4) containing 120 mM NaCl and 5 mM KCl. Incubation with the radioligand [^3^H]CGP12177 (specific activity: 37.7 Ci/mmol; NEN Life Science Products, Boston, MA, USA) at a concentration of 4 nM was performed for 60 min. Propranolol (10 µM; Sigma-Aldrich, Darmstadt, Germany) was used to determine nonspecific labeling. Sections were washed twice for 1 min in 50 mM Tris-HCl buffer (pH 7.4) at 4 °C. The sections were then dried in a stream of cool air.

#### 4.4.4. Quantitative Image Analysis of the Autoradiographs

Dried tissue sections were exposed for 10 days to tritium-sensitive screens Fujibas TR2024 (Fujifilm, Tokyo, Japan) along with [^3^H]microscales (Amersham Biosciences Corp., Piscataway, NJ, USA) as standard. The images, obtained employing a FujiFilm BAS 5000 Phosphorimager, were analyzed using FujiFilm software (Image Gauge, Version 3.0, FujiFilm, Tokyo, Japan) and quantified by computer-generated curves derived from the standards. The pixels of images from sections showing nonspecific binding were subtracted from those of adjacent sections with total binding. The results are expressed as fmol of bound radioligand per mg protein.

### 4.5. Measurement of Neurotransmitters and Their Metabolite Levels in Brain Tissue

The hypothalamic tissue level of noradrenaline, dopamine, its two metabolites 3,4-dihydroxyphenylacetic acid (DOPAC), and homovanillic acid (HVA), and serotonin, with its metabolite 5-hydroxyindoleacetic acid (5-HIAA) in the HY were assessed by ultra-high-performance liquid chromatography (UHPLC) with coulochemical detection using a previously described method with minor modifications [114,115]. Samples of the HY dissected from the brain of male mice were homogenized by sonification in 20 volumes (*v*/*w*) of ice-cold 0.1 M perchloric acid (HClO4). Homogenates were centrifuged at 15,000× *g* for 15 min at 4 °C. The obtained supernatants were transferred to new Eppendorf tubes, centrifuged at 15,000× *g* for 5 min at 4 °C, and filtered through a 0.2-μm membrane filter. The final samples were stored at −80 °C until further analysis. Subsequently, 10 μL aliquots were injected into the UHPLC Ultimate 3000 system Dionex (Thermo Scientific, Germering, Germany). The system used consisted of an ECD-3000RS electrochemical detector, 6011RS ultra coulometric analytical cell, WPS-3000RS autosampler, and a Hypersil Gold analytical column 3 µm, 100 × 3 mm (Thermo Scientific, Waltham, MA, USA). Neurotransmitters and their metabolites were eluted using the mobile phase, which included: KH2PO4 (0.1 M), EDTA (0.5 mM), sodium 1-octane sulfonate (80 mg/L), methanol (4%), adjusted to pH 4.0 with 85% H3PO4, at the flow rate 0.6 mL/min and the column temperature of 30 °C. The potentials of coulometric cells were: E1 = −50 mV, E2 = +350 mV [114]. The identification and quantification of the chromatographic peaks were made by comparison with the reference standard peaks: noradrenaline, dopamine, serotonin, DOPAC, and 5-HIAA (Sigma-Aldrich, Darmstadt, Germany) at concentrations of 50 ng/mL and HVA (Sigma-Aldrich, Darmstadt, Germany) at a concentration of 100 ng/mL [115]. The data were analyzed and processed using Thermo Scientific Dionex Chromoleon 7 Version 7.2.1.5833 (Thermo Scientific, Waltham, MA, USA) [114]. The limit of detection of noradrenaline, dopamine, DOPAC, HVA, and 5-HIAA was 0.5 pg/10 μL, and serotonin was 1 pg/10 μL.

### 4.6. RNA Isolation and Gene Expression Analysis

Prefrontal cortex brain tissue was stored in RNAlater stabilizing solution (Thermo Scientific, AM7021, Waltham, MA, USA) at −20 °C. Tissue was homogenized with TissueLyser II apparatus (Qiagen, Hilden, Germany) in Lysis Buffer provided as part of the RNeasy Mini kit (Qiagen, #74106, Hilden, Germany), which was used to isolate RNA, according to manufacturer’s instructions. Absorbance measurements determined the quantity and quality of RNA at 260/280 nm on NanoPhotometer (Implen, Munchen, Germany). Only high-quality RNA samples with an A260/A280 ratio of 1.8–2.0 were used for further analyses.

Reverse transcription was performed with a High Capacity cDNA Reverse Transcription Kit (Thermo Scientific, #4374966, Waltham, MA, USA) utilizing random primers and 1.5 µg of RNA per reaction in 15 µL volume. Reaction conditions were 25 °C for 10 min, 37 °C for 120 min, and 85 °C for 5 min. Gene expression analysis of adrenergic receptors was performed by quantitative real-time polymerase chain reaction (qPCR) using SYBR Green chemistry. The reaction was carried out in 96-well plates (MicroAmp^®^ Fast Optical 96-Well Reaction Plate, 0.1 mL, Thermo Scientific, #4346907, Waltham, MA, USA). In each well, there was a total of 10 µL reaction volume, containing 20 ng cDNA template, 5 µL of RT HS-PCR Mix SYBR A master mix (AA biotechnology, #2017-100A, Gdansk, Poland), and appropriate primers (see Supplementary Table S3). Each reaction was performed in triplicate on the QuantStudio 12 Flex (Thermo Scientific, Waltham, MA, USA) in the following conditions: 1 cycle at 50 °C for 2 min, one cycle at 95 °C for 5 min, followed by 40 cycles of: 95 °C for 15 s, 60 °C for 30 s, 72 °C for 30 s each. The threshold value (Ct) was automatically determined by QuantStudio 12K Flex Software v1.4 (Applied Biosystems, Waltham, MA, USA), and expression levels were calculated using the standard curve method. Phosphoglycerate kinase 1 (PGK1) and hypoxanthine-guanine phosphoribosyltransferase (HPRT) were reference genes.

### 4.7. Gene Expression Profiling

Gene expression analysis was performed in the hippocampus of male control (WT), A-KO, B-KO, and D-KO mice. Brain structures were dissected and preserved immediately in stabilization reagent RNAlater (ThermoFisher Scientific, MA, USA). Total RNA was isolated using the RNeasy Mini Kit (Qiagen, Hilden, Germany) strictly according to the manufacturer’s protocol. RNA integrity from all samples was spectrophotometrically assessed following quality control performed with Bioanalyzer 2100 (Agilent Technologies, CA, USA). Only the samples fulfilling the best quality criteria based on RIN number values (RIN > 8.0) were qualified for microarrays. RNA was reverse transcribed, and cDNA from individual animals was hybridized to a GeneChip Mouse Genome 430A 2.0 array (Affymetrix) for a total of 14 arrays. Among 14 samples that were dispatched for microarray probing, each group was represented by 3–4 biological replicates (i.e., there were 3–4 mice/group). These arrays include over 45,000 oligonucleotide probes that can detect more than 39,000 transcripts, allowing for whole-genome expression profiling of the mouse genome. Array data analyses were performed as described before [116]. Briefly, raw data were normalized, and expression values were computed using the affy and gcrma packages from R/Bioconductor [117]. Statistical analyses were performed against the untreated control group (control_veh), separately for drug treatment and mutation effects, to dissect the effects of the evaluated pharmaceuticals (MIL, DMI) and the effects of α1-AR subtype deletions. Gene ontology analyses were carried out using Gene Set Enrichment Analysis (GSEA) [118] and the Panther Classification System [119]. The set of significantly under- and over-represented genes upon treatment with MIL was further analyzed by DAVID Functional Annotation Bioinformatics Microarray Analysis, https://davidbioinformatics.nih.gov (accessed on 30 April 2025) [120,121]. MultiExperiment Viewer (MeV ver. 4.81 for Mac OS) was utilized to identify patterns of gene expression and data visualization (heat maps presented in the paper). The raw microarray data are stored in the GEO database, https://www.ncbi.nlm.nih.gov/geo/query/acc.cgi?acc=GSE272818 (accessed on 23 July 2024).

### 4.8. Western Blot Analyses of Protein Levels

Protein extraction, sample denaturation, and Western blot procedure were based on the protocols described elsewhere [122,123,124,125,126]. Briefly, radioimmunoprecipitation assay (RIPA) buffer (MilliporeSigma, Burlington, MA, USA) was used for total protein extraction. Equal amounts of protein extracts were boiled in Laemmli buffer containing 5% 2-mercaptoethanol for 5 min. Denatured samples were run on SDS-PAGE gels and then transferred to nitrocellulose membranes. Membranes were then blocked with 5% nonfat dry milk in Tris-buffered saline with 0.1% Tween-20 (TBST; pH = 7.6) for 1 h at room temperature and incubated with specific primary antibodies. After overnight incubation at 4 °C with primary antibodies and three washes with blocking solution, the membranes were incubated with appropriate secondary antibodies for 1 h at room temperature, followed by three washes with TBST. Antibody binding was detected using a Clarity Western ECL Substrate (Bio-Rad, Hercules, California, USA). Equal protein loading was confirmed by probing with anti-calnexin antiserum (1:1000; ADI-SPA-865-F, Enzo Life Sciences, Farmingdale, NY, USA). Phosphorylated and total protein levels were assessed on the same membrane, together with a loading control. The following antibodies were used in the experiment: pERK1/2 (1:2000, cat. sc-7383, Santa Cruz Biotechnology, Dallas, TX, USA); ERK1/2 (1:2000, cat. sc-93, Santa Cruz Biotechnology, Dallas, TX, USA); p(S473)Akt (1:2500, cat.4060, Cell Signaling Technology, Danvers, MA, USA); Akt (1:3000, cat. 9272, Cell Signaling Technology, Danvers, MA, USA); p(S21/9)GSK3α/β (1:1000, cat. 9331, Cell Signaling Technology, Danvers, MA, USA); GSK3α/β (1:1000, cat. 5676, Cell Signaling Technology, Danvers, MA, USA). All Western blot analyses were performed at least twice to confirm the results. The chemiluminescence of specific signals was visualized with the PXi4 (Syngene, Cambridge, UK), and the immunoreactive bands were quantified by an image analyzer (MultiGauge V3.0, Fujifilm, Tokyo, Japan). Original blot images for results presented in the manuscript are shown in Supplementary Figure S4.

## 5. Conclusions

To our knowledge, these studies demonstrate the functional differentiation of α1-AR subtypes in the brain for the first time in relation to the molecular effects induced by chronic antidepressant treatment. The results of this study, conducted in the mouse models with individual α1-AR subtype knockouts, showed that the inactivation of the α1D-AR subtype influences the effects of chronic treatments with MIL and, to a lesser extent, DMI, across various molecular levels. The pattern of drug-induced changes in phosphorylation of GSK3β, Akt, and Erk1/2—key components of intracellular signaling pathways involved in mood disorders—varied by gender. These findings emphasize the functional diversity among α1-AR subtypes in the mechanisms of antidepressants’ drugs action and suggest the promising potential of α1-AR receptor subtypes for translational research aimed at developing new treatments for neuropsychiatric disorders.

## Data Availability

The data are contained within the article and the Supplementary Materials. The raw microarray data are stored in the GEO database, https://www.ncbi.nlm.nih.gov/geo/query/acc.cgi?acc=GSE272818 (accessed on 23 July 2024).

## Supplementary Materials

The following supporting information can be downloaded at: https://www.mdpi.com/article/10.3390/ijms262110488/s1.

## Abbreviations

The following abbreviations are used in this manuscript:

α1-AR	α1-adrenergic receptors
Akt	Akt kinase, also known as protein kinase B (PKB)
CAM	constitutively active mutant
CNS	Central nervous system
DA	dopamine
DAG	diacylglycerol
DMI	desipramine
DOPAC	3,4-dihydroxyphenylacetic acid
ERK1/2	extracellular signal-regulated kinases 1/2
GPCR	G-protein-coupled receptor
GSK3β	Glycogen synthase kinase-3 beta
5-HIAA	5-hydroxy indole acetic acid
HIP	hippocampus
HPA	hypothalamic–pituitary–adrenal axis
HPRT	hypoxanthine-guanine phosphoribosyltransferase
5-HT	serotonin
HVA	homovanillic acid
HY	hypothalamus
IP3	Inositol trisphosphate
KEGG	Kyoto Encyclopedia of Genes and Genomes
LTP	long-term potentiation
MAOI	Monoamine oxidase inhibitors
MIL	milnacipran
MSA	Multiple system atrophy
NA	noradrenaline
NRI	Noradrenaline reuptake inhibitor
PFC	prefrontal cortex
PGK1	phosphoglycerate kinase 1
PI3K	phosphatidylinositol 3-kinase
PKC	protein kinase C
PLC	phospholipase Cβ
SNRI	Serotonin and noradrenaline reuptake inhibitors
SSRI	Serotonin selective reuptake inhibitors
TCA	tricyclic antidepressants
UHPLC	ultra-high-performance liquid chromatography
WT	wild-type
