# Supplementary material for: The Differential Involvement of α1-Adrenoceptor Subtypes in the Molecular Effects of Antidepressant Drugs
Source: Int J Mol Sci. 2025 Oct 28;26(21):10488. doi: 10.3390/ijms262110488 (PMC12610789; doi:10.3390/ijms262110488)
Supplement: Supplementary file 1 [file ijms-26-10488-s001.zip › Supplementary Figure S2_1027_Nalepa I. et al.pdf]

The differential involvement of α1-adrenoceptor subtypes in the molecular effects of antidepressant drugs

Irena Nalepa [1*], Katarzyna Chorązka [1], Grzegorz Kreiner [1], Agnieszka Zelek-Molik [1], Anna Haduch [2], Władysława Anna Daniel [2], Piotr Chmielarz [1], Katarzyna Maziarz [1], Justyna Kuśmierczyk [1#], Michał Wilczkowski [1], Adam Bielawski [1], Marta Kowalska [1]

[1]Department of Brain Biochemistry, Maj Institute of Pharmacology, Polish Academy of Sciences, Smętna 12, 31-343 Kraków, Poland; kreiner@if-pan.krakow.pl (G.K.); zelek@if-pan.krakow.pl (A.Z-M.); chmiel@if-pan.krakow.pl (P.C.); maziarz@if-pan.krakow.pl (K.M.); justyna.kusmierczyk@awf.krakow.pl (J.K.); wilczkow@if-pan.krakow.pl (M.W.); bielaw@if-pan.krakow.pl; (A.B.); marcik48@op.pl (M.K.)

[2]Department of Pharmacokinetics and Drug Metabolism, Maj Institute of Pharmacology, Polish Academy of Sciences, Smętna 12, 31-343 Kraków, Poland; haduch@if-pan.krakow.pl (A.H.); nfdaniel@cyf-kr.edu.pl (W.A.D.);

*Correspondence: nfnalepa@cyf-kr.edu.pl

Supplementary Figure S2

	Mus musculus (REF)	upload_1 (▽ Hierarchy NEW! ?)					
GO biological process complete	#	#	expected	Fold Enrichment	+/-	raw P value	FDR
ceramide biosynthetic process	45	8	1.08	7.44	+	2.95E-05	3.85E-02
↳metabolic process	7166	227	171.31	1.33	+	6.30E-07	4.94E-03
↳cellular metabolic process	6280	210	150.13	1.40	+	3.49E-08	5.48E-04
↳membrane lipid biosynthetic process	106	12	2.53	4.74	+	2.10E-05	4.11E-02
oligodendrocyte differentiation	63	10	1.51	6.64	+	7.35E-06	2.31E-02
glycolipid metabolic process	80	10	1.91	5.23	+	4.75E-05	4.97E-02
cellular protein metabolic process	2859	103	68.35	1.51	+	2.76E-05	3.94E-02
positive regulation of metabolic process	3419	119	81.73	1.46	+	2.70E-05	4.23E-02
↳positive regulation of biological process	5947	187	142.17	1.32	+	2.51E-05	4.37E-02
↳regulation of metabolic process	5688	181	135.98	1.33	+	1.90E-05	4.26E-02
negative regulation of cellular process	4482	151	107.15	1.41	+	7.27E-06	2.85E-02
↳negative regulation of biological process	5005	167	119.65	1.40	+	2.93E-06	1.53E-02
regulation of primary metabolic process	5098	166	121.87	1.36	+	1.47E-05	3.84E-02
regulation of nitrogen compound metabolic process	4952	160	118.38	1.35	+	3.88E-05	4.34E-02
regulation of cellular metabolic process	5288	169	126.41	1.34	+	3.72E-05	4.49E-02

Supplementary Figure S2. The transcriptomic analysis results indicate that the transcript levels in the hippocampus of wild-type mice were significantly altered due to repeated administration of milnacipran. This analysis was subjected to ontological evaluation using the Gene Set Enrichment Analysis (GSEA) method [96] and the Panther classification system [97].
