# Supplementary material for: The Differential Involvement of α1-Adrenoceptor Subtypes in the Molecular Effects of Antidepressant Drugs
Source: Int J Mol Sci. 2025 Oct 28;26(21):10488. doi: 10.3390/ijms262110488 (PMC12610789; doi:10.3390/ijms262110488)
Supplement: Supplementary file 1 [file ijms-26-10488-s001.zip › Supplementary Figure S3_1027_Nalepa et al.pdf]

The differential involvement of α1-adrenoceptor subtypes in the molecular effects of antidepressant drugs

Irena Nalepa [1*], Katarzyna Chorązka [1], Grzegorz Kreiner [1], Agnieszka Zelek-Molik [1], Anna Haduch [2], Władysława Anna Daniel [2], Piotr Chmielarz [1], Katarzyna Maziarz [1], Justyna Kuśmierczyk [1], Michał Wilczkowski [1], Adam Bielawski [1], Marta Kowalska [1]

[1]Department of Brain Biochemistry, Maj Institute of Pharmacology, Polish Academy of Sciences, Smętna 12, 31-343 Kraków, Poland; kreiner@if-pan.krakow.pl (G.K.); zelek@if-pan.krakow.pl (A.Z-M.); chmiel@if-pan.krakow.pl (P.C.); maziarz@if-pan.krakow.pl (K.M.); justyna.kusmierczyk@awf.krakow.pl (J.K.); wilczkow@if-pan.krakow.pl (M.W.); bielaw@if-pan.krakow.pl (A.B.); marcik48@op.pl (M.K.)

[2]Department of Pharmacokinetics and Drug Metabolism, Maj Institute of Pharmacology, Polish Academy of Sciences, Smętna 12, 31-343 Kraków, Poland; haduch@if-pan.krakow.pl (A.H.); nfdaniel@cyf-kr.edu.pl (W.A.D.);

*Correspondence: nfnalepa@cyf-kr.edu.pl

Supplementary Figure S3

Supplementary Figure S3

Current Gene List: List_2
Current Background: Mus musculus
573 DAVID IDs

Sublist	Category	Term	RT	Genes	Count	%	P-Value	Benjamini
	KEGG_PATHWAY	Huntington disease	RT		17	3,0	3,2E-3	6,3E-1
	KEGG_PATHWAY	Lysosome	RT		10	1,7	5,6E-3	6,3E-1
	KEGG_PATHWAY	Oxidative phosphorylation	RT		10	1,7	7,1E-3	6,3E-1
	KEGG_PATHWAY	N-Glycan biosynthesis	RT		6	1,0	9,5E-3	6,3E-1
	KEGG_PATHWAY	Mitophagy - animal	RT		8	1,4	1,1E-2	6,3E-1
	KEGG_PATHWAY	Parkinson disease	RT		14	2,4	1,6E-2	6,6E-1
	KEGG_PATHWAY	Diabetic cardiomyopathy	RT		12	2,1	1,6E-2	6,6E-1
	KEGG_PATHWAY	Amyotrophic lateral sclerosis	RT		17	3,0	1,9E-2	6,9E-1
	KEGG_PATHWAY	Cellular senescence	RT		10	1,7	3,3E-2	9,6E-1
	KEGG_PATHWAY	Glycosylphosphatidylinositol (GPI)-anchor biosynthesis	RT		4	0,7	3,5E-2	9,6E-1
	KEGG_PATHWAY	Human T-cell leukemia virus 1 infection	RT		12	2,1	3,7E-2	9,6E-1
	KEGG_PATHWAY	Non-alcoholic fatty liver disease	RT		9	1,6	4,3E-2	1,0E0
	KEGG_PATHWAY	Alzheimer disease	RT		16	2,8	5,0E-2	1,0E0
	KEGG_PATHWAY	Chemical carcinogenesis - reactive oxygen species	RT		11	1,9	5,1E-2	1,0E0
	KEGG_PATHWAY	MicroRNAs in cancer	RT		13	2,3	6,4E-2	1,0E0
	KEGG_PATHWAY	Metabolic pathways	RT		50	8,7	6,6E-2	1,0E0
	KEGG_PATHWAY	Prion disease	RT		12	2,1	6,7E-2	1,0E0

Current Gene List: List_1
Current Background: Mus musculus
245 DAVID IDs

Sublist	Category	Term	RT	Genes	Count	%	P-Value	Benjamini
	KEGG_PATHWAY	Huntington disease	RT		9	3,7	1,1E-2	1,0E0
	KEGG_PATHWAY	Kaposi sarcoma-associated herpesvirus infection	RT		7	2,9	2,2E-2	1,0E0
	KEGG_PATHWAY	Salmonella infection	RT		7	2,9	3,7E-2	1,0E0
	KEGG_PATHWAY	Prostate cancer	RT		4	1,6	7,5E-2	1,0E0
	KEGG_PATHWAY	MicroRNAs in cancer	RT		7	2,9	7,7E-2	1,0E0
	KEGG_PATHWAY	Influenza A	RT		5	2,0	9,2E-2	1,0E0

Supplementary Figure S3. KEGG pathway enrichment analysis of genes significantly over- and under-expressed after milnacipran (MIL) treatment in control and α1D-adrenergic receptor KO (D-KO) mice. Gene List_2 – genes over- and under-expressed in mice after chronic MIL treatment (WT MIL vs. WT SAL; full set of genes identified by expression profiling as statistically changed upon MIL treatment); Gene List_1 – genes over- and under-expressed in mice depleted of α1D-adrenergic receptor subtype after chronic MIL treatment (D-KO MIL vs. WT SAL). Term = KEGG Pathway Summary; RT/Genes/Count = genes involved in this annotation cluster visualized as a graphic percent of total genes analyzed and numerical value; % = percent of genes involved in this annotation; P-Value/Benjamin = threshold of EASE (Expression Analysis Systematic Explorer) score, a modified Fisher Exact P-value for gene-enrichment analysis.
