# Supplementary material for: The Differential Involvement of α1-Adrenoceptor Subtypes in the Molecular Effects of Antidepressant Drugs
Source: Int J Mol Sci. 2025 Oct 28;26(21):10488. doi: 10.3390/ijms262110488 (PMC12610789; doi:10.3390/ijms262110488)

The differential involvement of α1-adrenoceptor subtypes in the molecular effects of antidepressant drugs

Irena Nalepa [1*], Katarzyna Chorązka [1], Grzegorz Kreiner [1], Agnieszka Zelek-Molik [1], Anna Haduch [2], Władysława Anna Daniel [2], Piotr Chmielarz [1], Katarzyna Maziarz [1], Justyna Kuśmierczyk [1#], Michał Wilczkowski [1], Adam Bielawski [1], Marta Kowalska [1]

[1]Department of Brain Biochemistry, Maj Institute of Pharmacology, Polish Academy of Sciences, Smętna 12, 31-343 Kraków, Poland; kreiner@if-pan.krakow.pl (G.K.); zelek@if-pan.krakow.pl (A.Z-M.); chmiel@if-pan.krakow.pl (P.C.); maziarz@if-pan.krakow.pl (K.M.); justyna.kusmierczyk@awf.krakow.pl (J.K.); wilczkow@if-pan.krakow.pl (M.W.); bielaw@if-pan.krakow.pl (A.B.); marcik48@op.pl (M.K.)

[2]Department of Pharmacokinetics and Drug Metabolism, Maj Institute of Pharmacology, Polish Academy of Sciences, Smętna 12, 31-343 Kraków, Poland; haduch@if-pan.krakow.pl (A.H.); nfdaniel@cyf-kr.edu.pl (W.A.D.);

*Correspondence: nfnalepa@cyf-kr.edu.pl

Supplementary Figure S4

Supplementary Figure 4: Original blot images for the results presented in the manuscript.

Studied proteins (phosphorylation and total level) were assessed on the membrane from typical gels, together with a loading control. After protein transfer and membrane staining with Ponceau S, the membranes were cut horizontally to obtain optimal pieces for assessing the sound quality of bands predicted to meet the experimental goals, specifically molecular weight. The molecular weight of the studied proteins was verified in the initial experiment (performed for validation/optimization of the amount of Ab/protein taken for the experiment) using molecular markers (BioRad and/or Invitrogen). Only good-quality bands were analyzed. Repetitions for p/t ERK of analyzed bands are highlighted in colour in the tables, along with the sample description. A similar system, as described in the accompanying tables for arranging (alternating) experimental samples on the gel and performing replicates, was used for the remaining tested proteins (p/t AKT and p/t GSK3).

p/t ERK PROTEINS in HP, A-KO, male and female samples

GEL#1

No of band (from the left):	1	2	3	4	5	6	7	8	9	10	11	12	13	14	15	16	17	18	19	20	21	22	23	24
GROUP	WT female	WT male	KO a1A female	KO a1A male	WT female	WT male	KO a1A female	KO a1A male	WT female	WT male	KO a1A female	KO a1A male	WT female	WT male	KO a1A female	KO a1A male	WT female	KO a1A female	KO a1A male	WT female	KO a1A female	KO a1A male	WT female	WT female
ID	5	1	19	13	6	2	20	14	7	3	21	15	8	4	22	16	9	23	17	10	24	18	11	12

p ERK1/2 (upper blot) / t ERK1/2 (middle) / CNX (bottom)

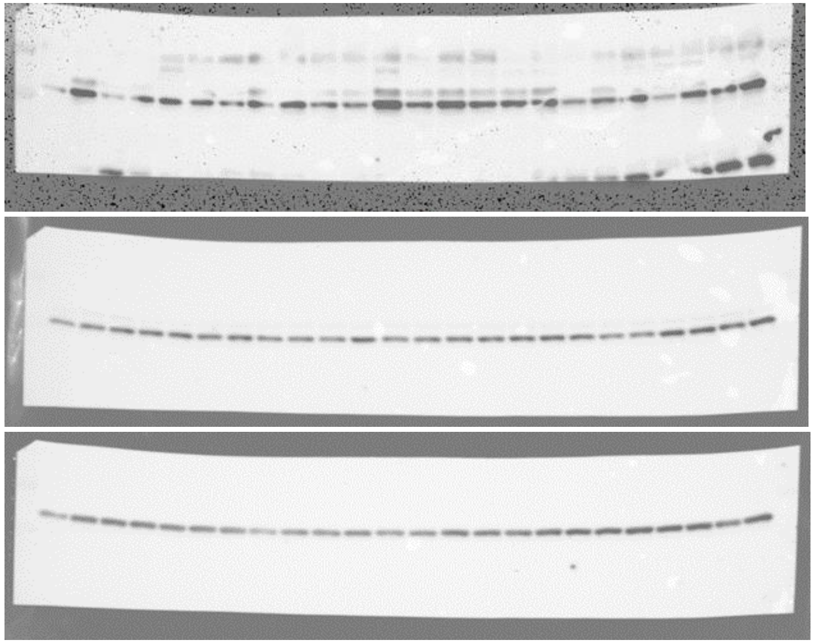

GEL#3

p ERK1/2 (upper blot) / t ERK1/2 (middle) / CNX (bottom)

No of band (from the left):	1	2	3	4	5	6	7	8	9	10	11	12	13	14	15	16	17	18	19	20	21	22	23	24
GROUP	WT female	WT male	KO a1A female	KO a1A male	WT female	WT male	KO a1A female	KO a1A male	WT female	WT male	KO a1A female	KO a1A male	WT female	WT male	KO a1A female	KO a1A male	WT female	KO a1A female	KO a1A male	WT female	KO a1A female	KO a1A male	WT female	WT female
ID	5	1	19	13	6	2	20	14	7	3	21	15	8	4	22	16	9	23	17	10	24	18	11	12

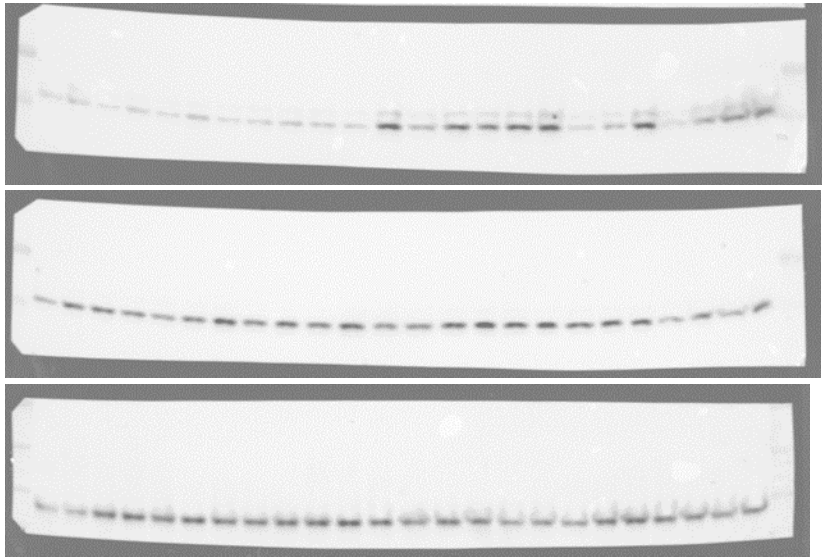

GEL#4

p ERK1/2 (upper blot) / t ERK1/2 (middle) / CNX (bottom)

No of band (from the left):	1	2	3	4	5	6	7	8	9	10	11	12	13	14	15	16	17	18	19	20	21	22	23	24
GROUP	WT female	WT male	KO a1A female	KO a1A male	WT female	WT male	KO a1A female	KO a1A male	WT female	WT male	KO a1A female	KO a1A male	WT female	WT male	KO a1A female	KO a1A male	WT female	KO a1A female	KO a1A male	WT female	KO a1A female	KO a1A male	WT female	WT female
ID	5	1	19	13	6	2	20	14	7	3	21	15	8	4	22	16	9	23	17	10	24	18	11	12

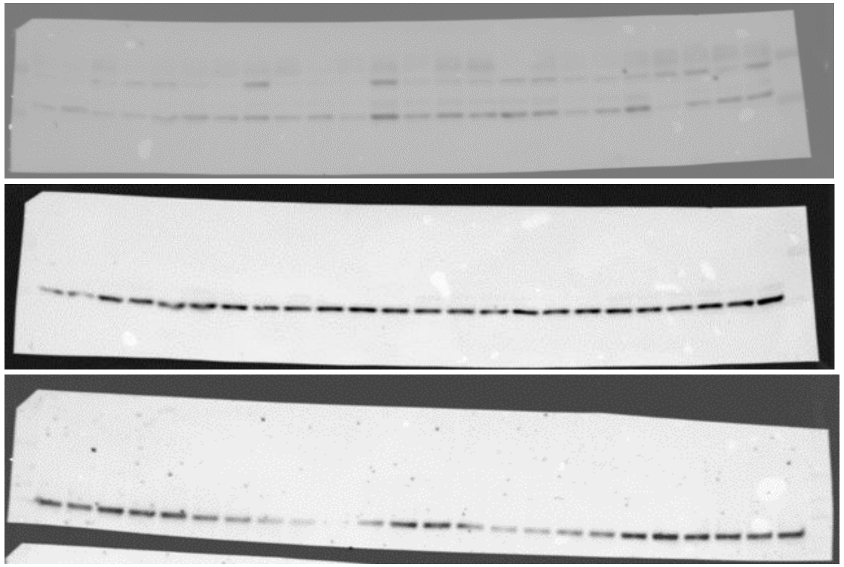

GEL#5

p ERK1/2 (upper blot) / t ERK1/2 (middle) / CNX (bottom)

No of band (from the left):	1	2	3	4	5	6	7	8	9	10	11	12	13	14	15	16	17	18	19	20	21	22	23	24
GROUP	WT female	WT male	KO a1A female	KO a1A male	WT female	WT male	KO a1A female	KO a1A male	WT female	WT male	KO a1A female	KO a1A male	WT female	WT male	KO a1A female	KO a1A male	WT female	KO a1A female	KO a1A male	WT female	KO a1A female	KO a1A male	WT female	WT female
ID	5	1	19	13	6	2	20	14	7	3	21	15	8	4	22	16	9	23	17	10	24	18	11	12

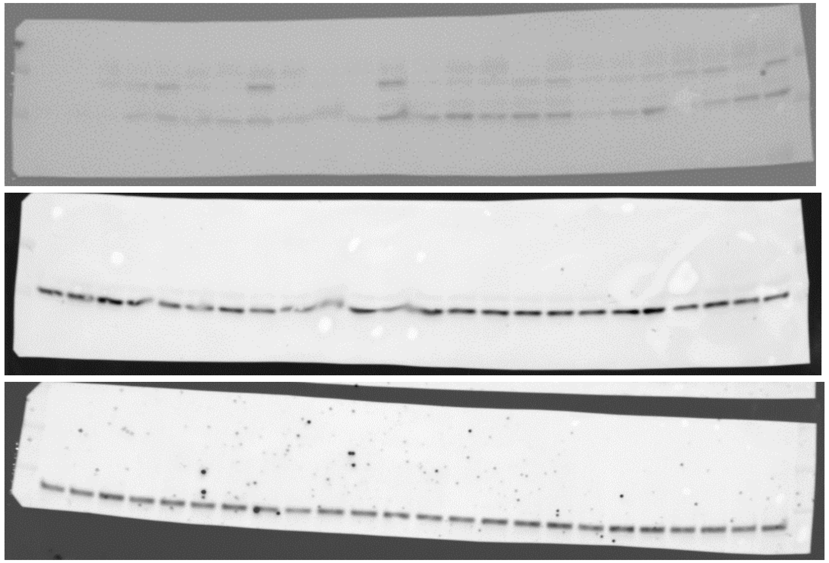

p/t ERK PROTEINS in B-KO male and female samples

GEL#3

p ERK1/2 (upper blot) / t ERK1/2 (middle) / CNX (bottom)

No of band (from the left):	1	2	3	4	5	6	7	8	9	10	11	12	13	14	15	16
GROUP	wt_sal_male	wt_sal_female	KO_a1B_sal_male	KO_a1B_sal_female	wt_sal_male	wt_sal_female	KO_a1B_sal_male	KO_a1B_sal_female	wt_sal_male	wt_sal_female	KO_a1B_sal_male	KO_a1B_sal_female	wt_sal_male	wt_sal_female	KO_a1B_sal_male	KO_a1B_sal_female
ID	failed	7.1	4.1	10.1	1.5	7.5	4.5	10.5	1.2	7.2	4.2	10.2	1.6	7.6	4.6	10.6

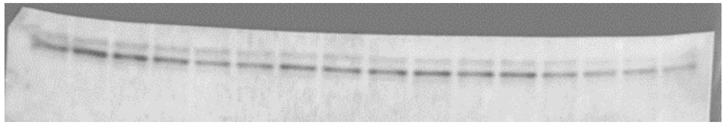

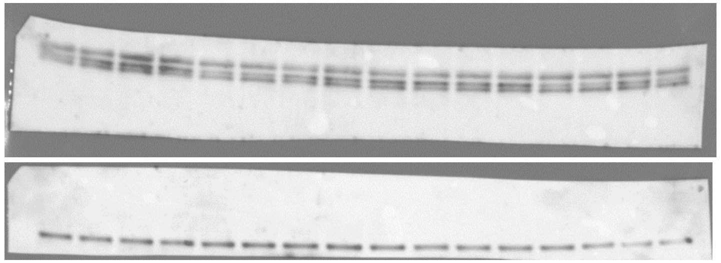

GEL#4

p ERK1/2 (upper blot) / t ERK1/2 (middle) / CNX (bottom)

No of band (from the left):	1	2	3	4	5	6	7	8	9	10	11	12	13	14	15
GROUP	wt_sal_male	wt_sal_female	KO_a1B_sal_male	KO_a1B_sal_female	wt_sal_male	wt_sal_female	KO_a1B_sal_male	KO_a1B_sal_female	wt_sal_male	wt_sal_female	KO_a1B_sal_male	KO_a1B_sal_female	wt_sal_male	wt_sal_female	KO_a1B_sal_female
ID	failed	failed	failed	failed	failed	7.7	4.7	10.7	1.4	7.4	4.4	10.4	1.8	7.8	10.8

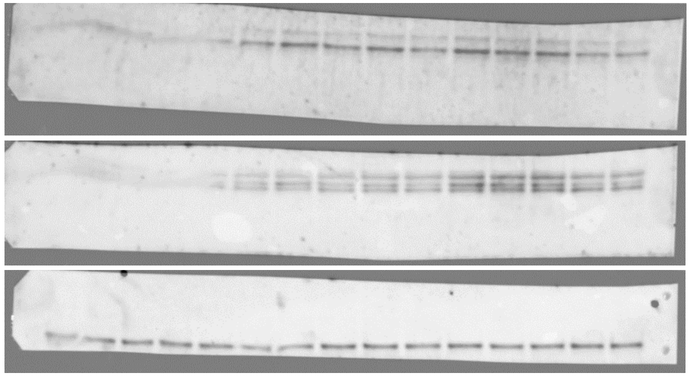

GEL#5

p ERK1/2 (upper blot) / t ERK1/2 (middle) / CNX (bottom)

No of band (from the left):	1	2	3	4	5	6	7	8	9	10	11	12	13	14	15	16
GROUP	wt_sal_male	wt_sal_female	KO_a1B_sal_male	KO_a1B_sal_female	wt_sal_male	wt_sal_female	KO_a1B_sal_male	KO_a1B_sal_female	wt_sal_male	wt_sal_female	KO_a1B_sal_male	KO_a1B_sal_female	wt_sal_male	wt_sal_female	KO_a1B_sal_male	KO_a1B_sal
ID	failed	failed	failed	failed	1.5	7.5	4.5	10.5	1.2	7.2	4.2	10.2	1.6	7.6	4.6	10.6

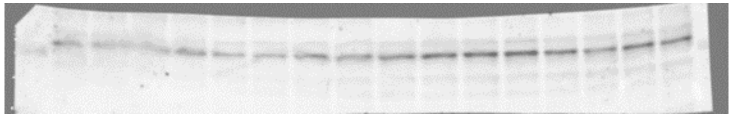

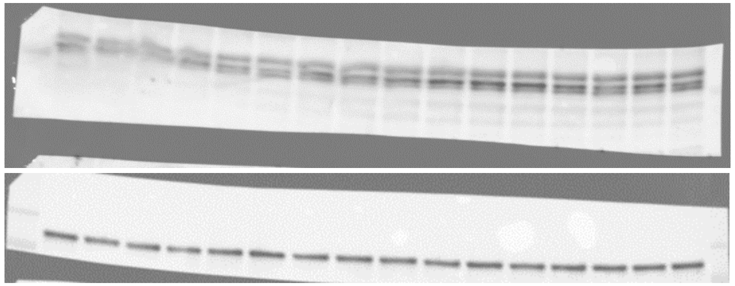

GEL#6

p ERK1/2 (upper blot) / t ERK1/2 (middle) / CNX (bottom)

No of band (from the left):	1	2	3	4	5	6	7	8	9	10	11	12	13	14	15
GROUP	wt_sal_male	wt_sal_female	KO_a1B_sal_male	KO_a1B_sal_female	wt_sal_male	wt_sal_female	KO_a1B_sal_male	KO_a1B_sal_female	wt_sal_male	wt_sal_female	KO_a1B_sal_male	KO_a1B_sal_female	wt_sal_male	wt_sal_female	KO_a1B_sal_fema
ID	failed	failed	failed	failed	1.7	7.7	4.7	10.7	1.4	7.4	4.4	10.4	1.8	7.8	10.8

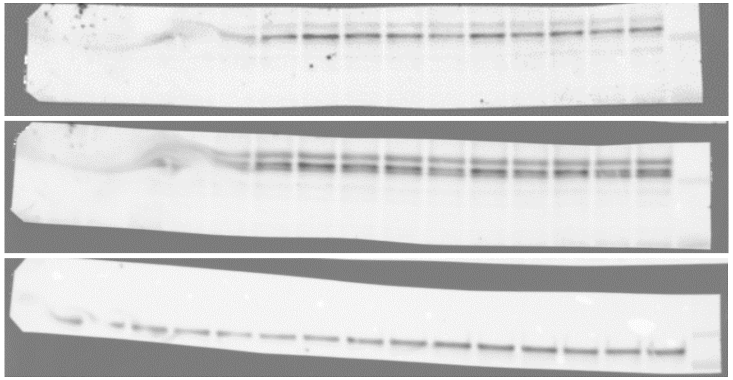

GEL#7

p ERK1/2 (upper blot) / t ERK1/2 (middle) / CNX (bottom)

No of band (from the left):	1	2	3	4	5	6	7	8	9	10	11	12	13	14	15	16
GROUP	wt_sal_male	wt_sal_female	KO_a1B_sal_male	KO_a1B_sal_female	wt_sal_male	wt_sal_female	KO_a1B_sal_male	KO_a1B_sal_female	wt_sal_male	wt_sal_female	KO_a1B_sal_male	KO_a1B_sal_female	wt_sal_male	wt_sal_female	KO_a1B_sal_male	KO_a1B_sal
ID	1.1	7.1	4.1	10.1	1.5	7.5	4.5	10.5	1.2	7.2	4.2	10.2	1.6	7.6	4.6	10.6

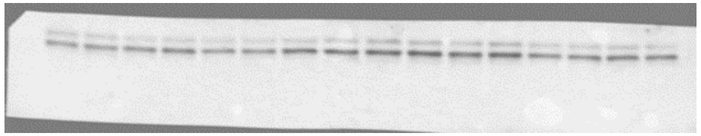

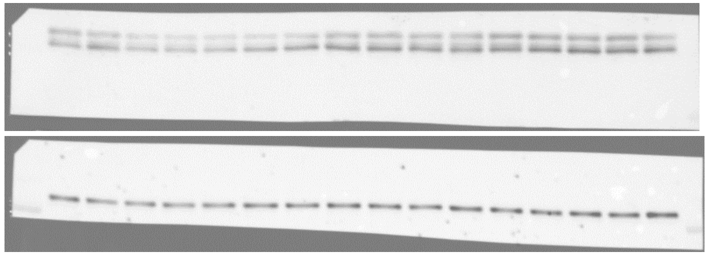

GEL#8

p ERK1/2 (upper blot) / t ERK1/2 (middle) / CNX (bottom)

No of band (from the left):	1	2	3	4	5	6	7	8	9	10	11	12	13	14	15
GROUP	wt_sal_male	wt_sal_female	KO_a1B_sal_male	KO_a1B_sal_female	wt_sal_male	wt_sal_female	KO_a1B_sal_male	KO_a1B_sal_female	wt_sal_male	wt_sal_female	KO_a1B_sal_male	KO_a1B_sal_female	wt_sal_male	wt_sal_female	KO_a1B_sal_femal
ID	1.3	7.3	4.3	10.3	1.7	7.7	4.7	10.7	1.4	7.4	4.4	10.4	1.8	7.8	10.8

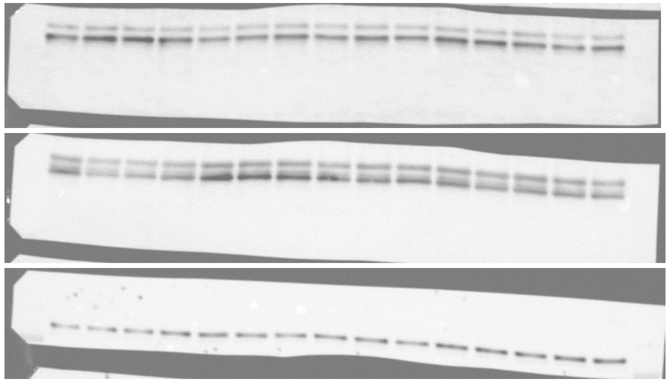

p/t ERK PROTEINS in D-KO, male samples

GEL# 13

p ERK1/2 (upper blot) / t ERK1/2 (middle) / CNX (bottom)

Band from left	1	2	3	4	5	6	7	8	9	10	11	12
Group	wt_sal_male	wt_DMI_male	wt_MIL_male	KO_a1D_sal_male	KO_a1D_DMI_male	KO_a1D_MIL_male	wt_sal_male	wt_DMI_male	wt_MIL_male	KO_a1D_sal_male	KO_a1D_DMI_male	KO_a1D_MIL_male
ID	7050	7078	7741	7060	7095	7749	7051	7086	7742	7062	7096	7754

Band from left	13	14	15	16	17	18	19	20	21	22	23	24
Group	wt_sal_male	wt_DMI_male	wt_MIL_male	KO_a1D_sal_male	KO_a1D_DMI_male	KO_a1D_MIL_male	wt_sal_male	wt_DMI_male	wt_MIL_male	KO_a1D_sal_male	KO_a1D_DMI_male	KO_a1D_MIL_male
ID	7066	7123	7753	7067	7121	7756	7068	7126	7771	7070	7132	7757

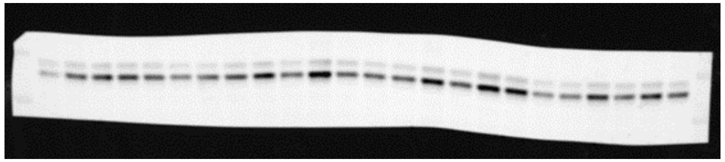

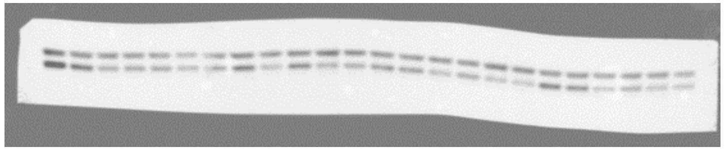

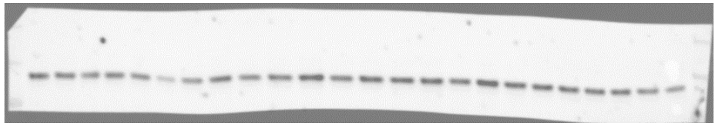

GEL# 14

p ERK1/2 (upper blot) / t ERK1/2 (middle) / CNX (bottom)

Band from left	1	2	3	4	5	6	7	8	9	10	11	12	13	14	15	16	17	18	19	20	21	22	23	24
Group	wt_sal_male	wt_DMI_male	wt_MIL_male	KO_a1D_sal_male	KO_a1D_DMI_male	KO_a1D_MIL_male	wt_sal_male	wt_DMI_male	wt_MIL_male	KO_a1D_sal_male	KO_a1D_DMI_male	KO_a1D_MIL_male	wt_sal_male	wt_DMI_male	wt_MIL_male	KO_a1D_sal_male	KO_a1D_DMI_male	KO_a1D_MIL_male	wt_sal_male	wt_DMI_male	wt_MIL_male	KO_a1D_sal_male	KO_a1D_DMI_male	KO_a1D_MIL_male
ID	7050	7078	7741	7060	7095	7749	7051	7086	7742	7062	7096	7754	7066	7123	7753	7067	7121	7756	7068	7126	7771	7070	7132	7757

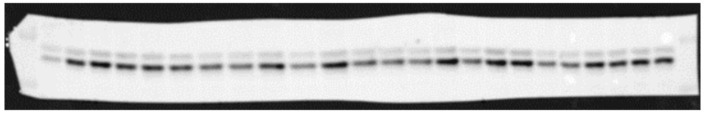

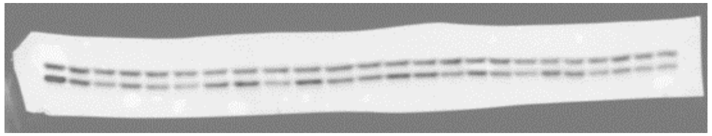

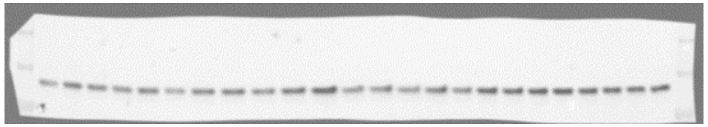

GEL# 15

p ERK1/2 (upper blot) / t ERK1/2 (middle) / CNX (bottom)

Band from left	1	2	3	4	5	6	7	8	9	10	11	12	13	14	15	16	17	18	19	20	21	22	23	24
Group	wt_sal_male	wt_DMI_male	wt_MIL_male	KO_a1D_sal_male	KO_a1D_DMI_male	KO_a1D_MIL_male	wt_sal_male	wt_DMI_male	wt_MIL_male	KO_a1D_sal_male	KO_a1D_DMI_male	KO_a1D_MIL_male	wt_sal_male	wt_DMI_male	wt_MIL_male	KO_a1D_sal_male	KO_a1D_DMI_male	KO_a1D_MIL_male	wt_sal_male	wt_DMI_male	wt_MIL_male	KO_a1D_sal_male	KO_a1D_DMI_male	KO_a1D_MIL_male
ID	7100	7148	7774	7071	7133	7749	7101	7158	7818	7109	7142	7754	7102	7162	7823	7110	7149	7756	7103	7162	7826	7787	7794	7757

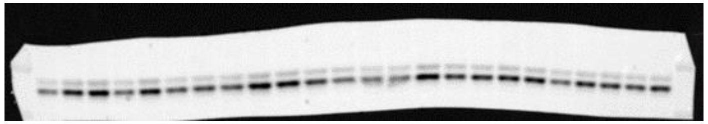

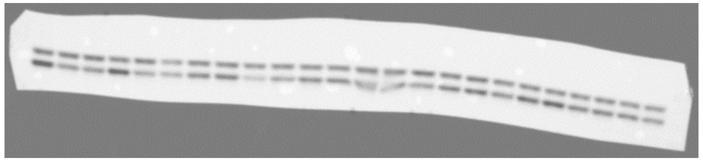

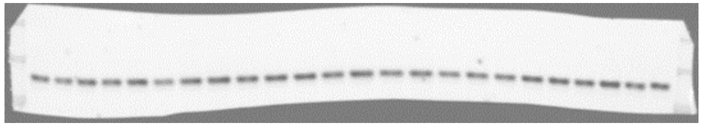

GEL# 16

p ERK1/2 (upper blot) / t ERK1/2 (middle) / CNX (bottom)

Band from left	1	2	3	4	5	6	7	8	9	10	11	12	13	14	15	16	17	18	19	20	21	22	23	24
Group	wt_sal_ male	wt_DMI_ male	wt_MIL_ male	KO_a1D_sal _male	KO_a1D_DMI _male	KO_a1D_MIL _male	wt_sal_ male	wt_DMI_ male	wt_MIL_ male	KO_a1D_sal _male	KO_a1D_DMI _male	KO_a1D_MIL _male	wt_sal_ male	wt_DMI_ male	wt_MIL_ male	KO_a1D_sal_ male	KO_a1D_DMI_ male	KO_a1D_MIL _male	wt_sal_ male	wt_DMI_ male	wt_MIL_ male	KO_a1D_sal _male	KO_a1D_D MI_male	KO_a1D_MIL _male
ID	7100	7148	7774	7071	7133	7749	7101	7158	7818	7109	7142	7754	7102	7162	7823	7110	7149	7756	7103	7162	7826	7787	7794	7757

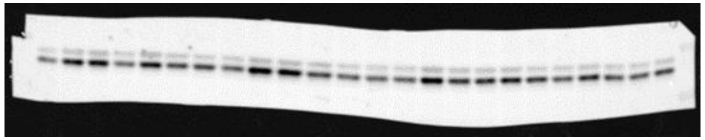

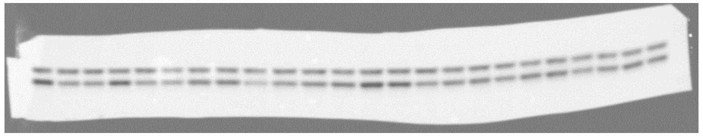

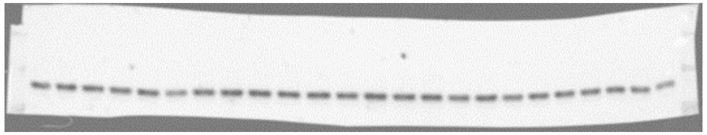

p/t ERK PROTEINS in D-KO, female samples

GEL#5

p ERK1/2 (upper blot) / t ERK1/2 (middle) / CNX (bottom)

Band from left	1	2	3	4	5	6	7	8	9	10	11	12	13	14	15	16	17	18	19	20	21	22	23	24
Group	wt_sal_ female	wt_DMI_ female	wt_MIL_ female	KO_a1D_sal _female	KO_a1D_D MI_female	KO_a1D_MIL _female	wt_sal_ female	wt_DMI_ female	wt_MIL_ female	KO_a1D_sal _female	KO_a1D_DMI _female	KO_a1D_MIL _female	wt_sal_ female	wt_DMI_ female	wt_MIL_ female	KO_a1D_sal _female	KO_a1D_DMI _female	KO_a1D_MIL _female	wt_sal_ female	wt_DMI_ female	wt_MIL_ female	KO_a1D_sal _female	KO_a1D_DMI _female	KO_a1D_MIL _female
ID	7052	7080	7775	7072	7097	7751	7053	7081	7777	7073	7098	7758	7054	7082	7800	7074	7099	7792	7055	7083	7801	7075	7116	7797

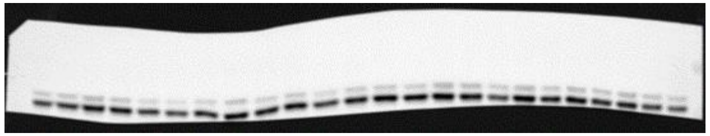

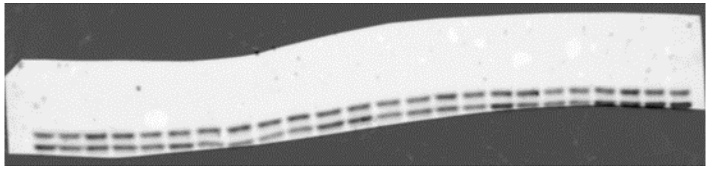

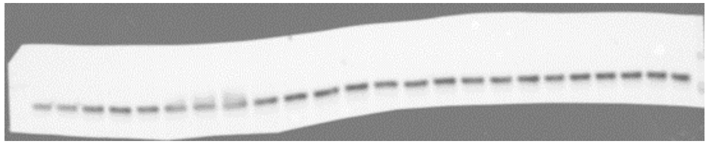

GEL#6

p ERK1/2 (upper blot) / t ERK1/2 (middle) / CNX (bottom)

Band from left	1	2	3	4	5	6	7	8	9	10	11	12
Group	wt_sal_female	wt_DMI_female	wt_MIL_female	KO_a1D_sal_female	KO_a1D_DMI_female	KO_a1D_MIL_female	wt_sal_female	wt_DMI_female	wt_MIL_female	KO_a1D_sal_female	KO_a1D_DMI_female	KO_a1D_MIL_female
ID	7052	7080	7775	7072	7097	7751	7053	7081	7777	7073	7098	7758

Band from left	13	14	15	16	17	18	19	20	21	22	23	24
Group	wt_sal_female	wt_DMI_female	wt_MIL_female	KO_a1D_sal_female	KO_a1D_DMI_female	KO_a1D_MIL_female	wt_sal_female	wt_DMI_female	wt_MIL_female	KO_a1D_sal_female	KO_a1D_DMI_female	KO_a1D_MIL_female
ID	7054	7082	7800	7074	7099	7792	7055	7083	7801	7075	7116	7797

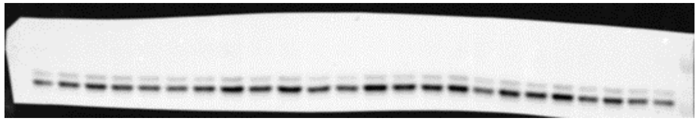

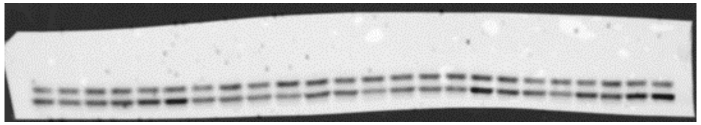

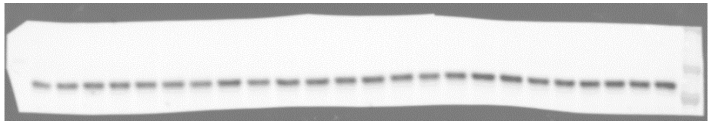

GEL#7

p ERK1/2 (upper blot) / t ERK1/2 (middle) / CNX (bottom)

Band from left	1	2	3	4	5	6	7	8	9	10	11	12
Group	wt_sal_female	wt_DMI_female	wt_MIL_female	KO_a1D_sal_female	KO_a1D_DMI_female	KO_a1D_MIL_female	wt_sal_female	wt_DMI_female	wt_MIL_female	KO_a1D_sal_female	KO_a1D_DMI_female	KO_a1D_MIL_female
ID	7056	7104	7809	7076	7138	7804	7745	7105	7835	7094	7143	7814

Band from left	13	14	15	16	17	18	19	20	21	22	23	24
Group	wt_sal_female	wt_DMI_female	wt_MIL_female	KO_a1D_sal_female	KO_a1D_DMI_female	KO_a1D_MIL_female	wt_sal_female	wt_DMI_female	wt_MIL_female	KO_a1D_sal_female	KO_a1D_DMI_female	KO_a1D_MIL_female
ID	7091	7106	7848	7764	7743	7820	7092	7107	7859	7833	7744	7840

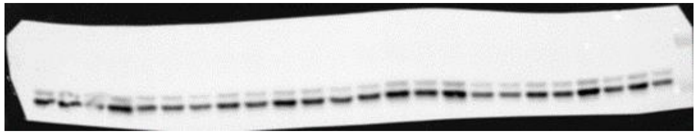

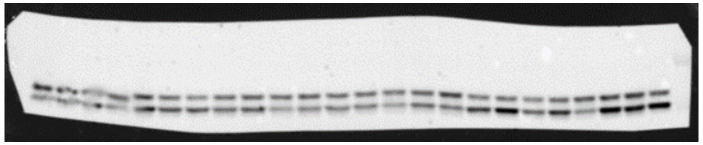

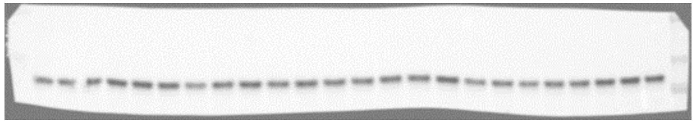

GEL#8

p ERK1/2 (upper blot) / t ERK1/2 (middle) / CNX (bottom)

Band from left	1	2	3	4	5	6	7	8	9	10	11	12	13	14	15	16	17	18	19	20	21	22	23	24
Group	wt_sal_female	wt_DMI_female	wt_MIL_female	KO_a1D_sal_female	KO_a1D_DMI_female	KO_a1D_MIL_female	wt_sal_female	wt_DMI_female	wt_MIL_female	KO_a1D_sal_female	KO_a1D_DMI_female	KO_a1D_MIL_female	wt_sal_female	wt_DMI_female	wt_MIL_female	KO_a1D_sal_female	KO_a1D_DMI_female	KO_a1D_MIL_female	wt_sal_female	wt_DMI_female	wt_MIL_female	KO_a1D_sal_female	KO_a1D_DMI_female	KO_a1D_MIL_female
ID	7056	7104	7809	7076	7138	7804	7745	7105	7835	7094	7143	7814	7091	7106	7848	7764	7743	7820	7092	7107	7859	7833	7744	7840

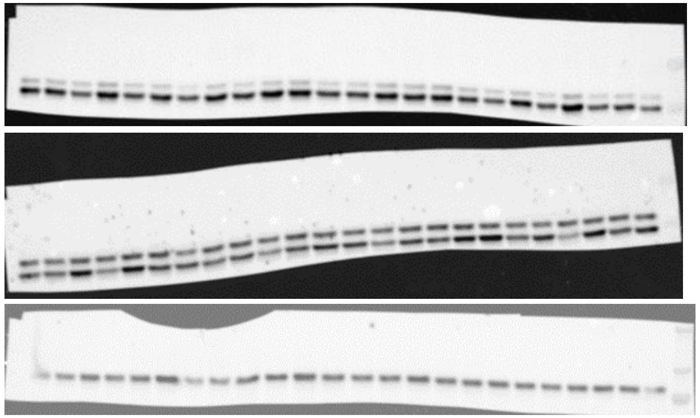

GEL#29

p ERK1/2 (upper blot) / t ERK1/2 (middle) / CNX (bottom)

Band from left	1	2	3	4	5	6	7	8	9	10	11	12	13	14	15	16	17	18	19	20	21	22	23	24
Group	wt_sal_female	wt_DMI_female	wt_MIL_female	KO_a1D_sal_female	KO_a1D_DMI_female	KO_a1D_MIL_female	wt_sal_female	wt_DMI_female	wt_MIL_female	KO_a1D_sal_female	KO_a1D_DMI_female	KO_a1D_MIL_female	wt_sal_female	wt_DMI_female	wt_MIL_female	KO_a1D_sal_female	KO_a1D_DMI_female	KO_a1D_MIL_female	wt_sal_female	wt_DMI_female	wt_MIL_female	KO_a1D_sal_female	KO_a1D_DMI_female	KO_a1D_MIL_female
ID	7052	7080	7775	7072	7097	7751	7053	7081	7777	7073	7098	7758	7054	7082	7800	7074	7099	7792	7055	7083	7801	7075	7116	7797

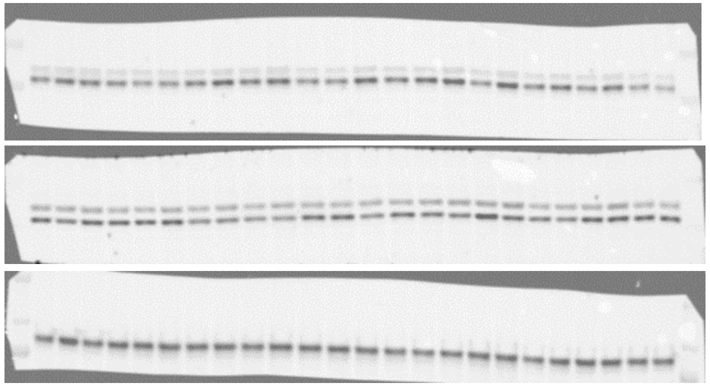

p/t AKT PROTEINS in HP, A-KO, male and female samples

GEL#7

pAkt (upper blot) / tAkt (middle) / CNX (bottom)

GEL# 7																								
Band	1	2	3	4	5	6	7	8	9	10	11	12	13	14	15	16	17	18	19	20	21	22	23	24
GROUP	wt_sal_male	wt_DMI_male	wt_MIL_male	KO_a1A_sal_male	KO_a1A_DMI_male	KO_a1A_MIL_male	wt_sal_female	wt_DMI_female	wt_MIL_female	KO_a1A_sal_female	KO_a1A_DMI_female	KO_a1A_MIL_female	wt_sal_male	wt_DMI_male	wt_MIL_male	KO_a1A_sal_male	KO_a1A_DMI_male	KO_a1A_MIL_male	wt_sal_female	wt_DMI_female	wt_MIL_female	KO_a1A_sal_female	KO_a1A_DMI_female	KO_a1A_MIL_female
ID	6515	6571	7899	6513	6572	7870	6523	6590	7908	6543	6511	7863	6527	6574	7900	6531	6573	7871	6524	6591	7909	6544	6576	7891

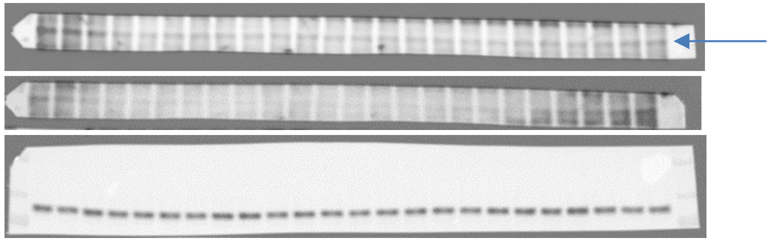

GEL#9

pAkt (upper blot) / tAkt (middle) / CNX (bottom)

GEL# 9																								
Band	1	2	3	4	5	6	7	8	9	10	11	12	13	14	15	16	17	18	19	20	21	22	23	24
GROUP	wt_sal_male	wt_DMI_male	wt_MIL_male	KO_a1A_sal_male	KO_a1A_DMI_male	KO_a1A_MIL_male	wt_sal_female	wt_DMI_female	wt_MIL_female	KO_a1A_sal_female	KO_a1A_DMI_female	KO_a1A_MIL_female	wt_sal_male	wt_DMI_male	wt_MIL_male	KO_a1A_sal_male	KO_a1A_DMI_male	KO_a1A_MIL_male	wt_sal_female	wt_DMI_female	wt_MIL_female	KO_a1A_sal_female	KO_a1A_DMI_female	KO_a1A_MIL_female
ID	failed	failed	failed	6537	6579	7886	6532	7003	7910	6558	6584	7895	6539	7028	7902	6553	6580	7888	6533	7004	7928	6559	6594	7897

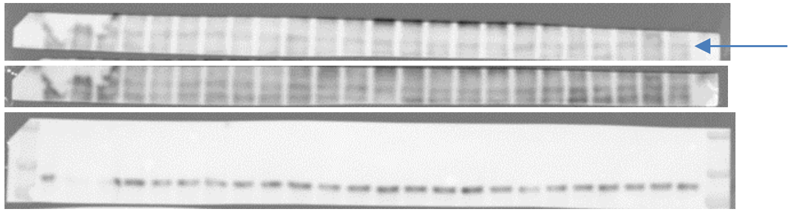

GEL#10

pAkt (upper blot) / tAkt (middle) / CNX (bottom)

GEL# 10																								
Band	1	2	3	4	5	6	7	8	9	10	11	12	13	14	15	16	17	18	19	20	21	22	23	24
GROUP	wt_sal_male	wt_DMI_male	wt_MIL_male	KO_a1A_sal_male	KO_a1A_DMI_male	KO_a1A_MIL_male	wt_sal_female	wt_DMI_female	wt_MIL_female	KO_a1A_sal_female	KO_a1A_DMI_female	KO_a1A_MIL_female	wt_sal_male	wt_DMI_male	wt_MIL_male	KO_a1A_sal_male	KO_a1A_DMI_male	KO_a1A_MIL_male	wt_sal_female	wt_DMI_female	wt_MIL_female	KO_a1A_sal_female	KO_a1A_DMI_female	KO_a1A_MIL_female
ID	6546	7036	7906	6554	6586	7905	6551	7005	7983	6565	6596	7927	6547	7043	7935	6555	7024	7950	6552	7006	7984	6566	6598	7955

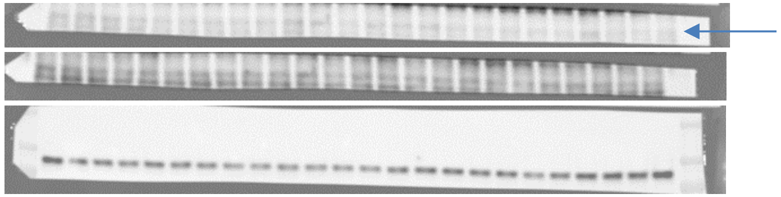

GEL#16

pAkt (upper blot) / tAkt (middle) / CNX (bottom)

GEL#16																								
Band	1	2	3	4	5	6	7	8	9	10	11	12	13	14	15	16	17	18	19	20	21	22	23	24
GROUP	wt_sal_male	wt_DMI_male	wt_MIL_male	KO_a1A_sal_male	KO_a1A_DMI_male	KO_a1A_MIL_male	wt_sal_female	wt_DMI_female	wt_MIL_female	KO_a1A_sal_female	KO_a1A_DMI_female	KO_a1A_MIL_female	wt_sal_male	wt_DMI_male	wt_MIL_male	KO_a1A_sal_male	KO_a1A_DMI_male	KO_a1A_MIL_male	wt_sal_female	wt_DMI_female	wt_MIL_female	KO_a1A_sal_female	KO_a1A_DMI_female	KO_a1A_MIL_female
ID	6515	6571	7899	6513	6572	7870	6523	6590	7908	6543	6511	7863	6527	6574	7900	6531	6573	7871	6524	6591	7909	6544	6576	7891

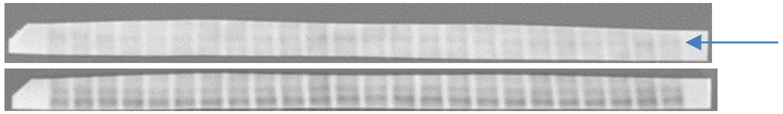

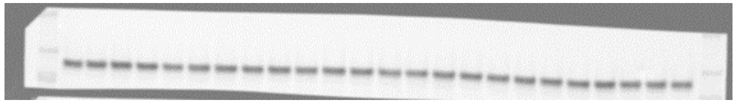

GEL#17

pAkt (upper blot) / tAkt (middle) / CNX (bottom)

GEL#17																								
Band	1	2	3	4	5	6	7	8	9	10	11	12	13	14	15	16	17	18	19	20	21	22	23	24
GROUP	wt_sal_male	wt_DMI_male	wt_MIL_male	KO_a1A_sal_male	KO_a1A_DMI_male	KO_a1A_MIL_male	wt_sal_female	wt_DMI_female	wt_MIL_female	KO_a1A_sal_female	KO_a1A_DMI_female	KO_a1A_MIL_female	wt_sal_male	wt_DMI_male	wt_MIL_male	KO_a1A_sal_male	KO_a1A_DMI_male	KO_a1A_MIL_male	wt_sal_female	wt_DMI_female	wt_MIL_female	KO_a1A_sal_female	KO_a1A_DMI_female	KO_a1A_MIL_female
ID	6530	7026	7901	6537	6579	failed	6532	7003	7910	6558	6584	7895	6539	7028	7902	6553	6580	7888	6533	7004	7928	6559	6594	7897

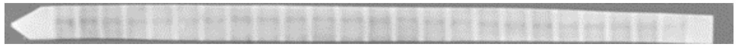

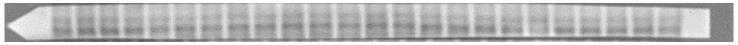

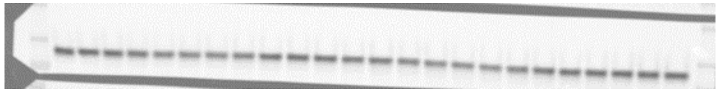

p/t AKT PROTEINS in B-KO

B-KO -MALE mice:

GEL#13																								
BAND from left	1	2	3	4	5	6	7	8	9	10	11	12	13	14	15	16	17	18	19	20	21	22	23	24
GROUP	wt_sal_male	wt_DMI_male	wt_MIL_male	KO_a1B_sal_male	KO_a1B_DMI_male	KO_a1B_MIL_male	wt_sal_male	wt_DMI_male	wt_MIL_male	KO_a1B_sal_male	KO_a1B_DMI_male	KO_a1B_MIL_male	wt_sal_male	wt_DMI_male	wt_MIL_male	KO_a1B_sal_male	KO_a1B_DMI_male	KO_a1B_MIL_male	wt_sal_male	wt_DMI_male	wt_MIL_male	KO_a1B_sal_male	KO_a1B_DMI_male	KO_a1B_MIL_male
ID	5114	5113	6431	5122	5105	6437	5115	5151	6432	5123	5107	6438	5141	5152	6433	5126	5108	6439	5142	5153	6444	5134	5119	6440

GEL#14																								
BAND from left	1	2	3	4	5	6	7	8	9	10	11	12	13	14	15	16	17	18	19	20	21	22	23	24
GROUP	wt_sal_male	wt_DMI_male	wt_MIL_male	KO_a1B_sal_male	KO_a1B_DMI_male	KO_a1B_MIL_male	wt_sal_male	wt_DMI_male	wt_MIL_male	KO_a1B_sal_male	KO_a1B_DMI_male	KO_a1B_MIL_male	wt_sal_male	wt_DMI_male	wt_MIL_male	KO_a1B_sal_male	KO_a1B_DMI_male	KO_a1B_MIL_male	wt_sal_male	wt_DMI_male	wt_MIL_male	KO_a1B_sal_male	KO_a1B_DMI_male	KO_a1B_MIL_male
ID	5143	5154	6445	5135	5120	6468	5144	5155	6446	5136	5121	6469	6418	6491	6480	*failed*	6451	6470	6419	6492	*failed*	6416	6453	6471

GEL#15																								
BAND from left	1	2	3	4	5	6	7	8	9	10	11	12	13	14	15	16	17	18	19	20	21	22	23	24
GROUP	wt_sal_male	wt_DMI_male	wt_MIL_male	KO_a1B_sal_male	KO_a1B_DMI_male	KO_a1B_MIL_male	wt_sal_male	wt_DMI_male	wt_MIL_male	KO_a1B_sal_male	KO_a1B_DMI_male	KO_a1B_MIL_male	wt_sal_male	wt_DMI_male	wt_MIL_male	KO_a1B_sal_male	KO_a1B_DMI_male	KO_a1B_MIL_male	wt_sal_male	wt_DMI_male	wt_MIL_male	KO_a1B_sal_male	KO_a1B_DMI_male	KO_a1B_MIL_male
ID	5114	5113	6431	5122	5105	6437	5115	5151	6432	5123	5107	6438	5141	5152	6433	5126	5108	6439	5142	5153	6444	5134	5119	6440

GEL#16																								
BAND from left	1	2	3	4	5	6	7	8	9	10	11	12	13	14	15	16	17	18	19	20	21	22	23	24
GROUP	wt_sal_male	wt_DMI_male	wt_MIL_male	KO_a1B_sal_male	KO_a1B_DMI_male	KO_a1B_MIL_male	wt_sal_male	wt_DMI_male	wt_MIL_male	KO_a1B_sal_male	KO_a1B_DMI_male	KO_a1B_MIL_male	wt_sal_male	wt_DMI_male	wt_MIL_male	KO_a1B_sal_male	KO_a1B_DMI_male	KO_a1B_MIL_male	wt_sal_male	wt_DMI_male	wt_MIL_male	KO_a1B_sal_male	KO_a1B_DMI_male	KO_a1B_MIL_male
ID	5143	5154	6445	5135	5120	6468	5144	5155	6446	5136	5121	6469	6418	6491	6480	*failed*	6451	6470	6419	6492	brak	6416	6453	6471

p AKT from up to bottom: GEL#13, GEL#14; GEL#15; GEL#16

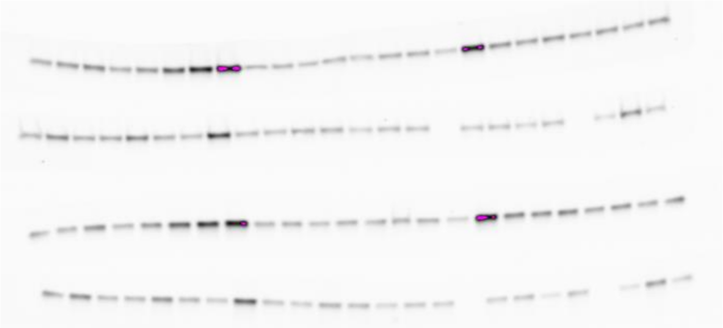

t AKT from up to bottom: GEL#13, GEL#14; GEL#15; GEL#16

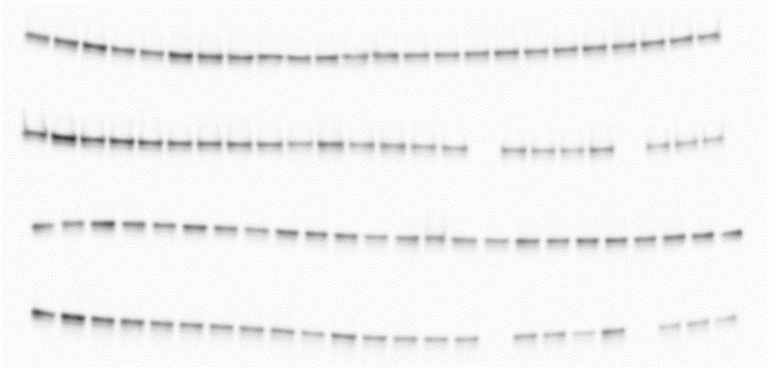

βACTIN from up to bottom: GEL#13, GEL#14; GEL#15; GEL#16

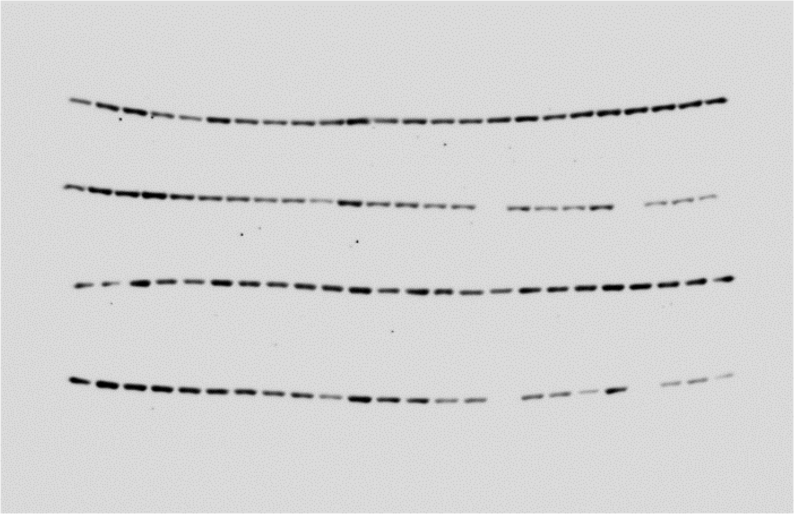

B-KO -FEMALE mice:

GEL#9																								
BAND from left	1	2	3	4	5	6	7	8	9	10	11	12	13	14	15	16	17	18	19	20	21	22	23	24
GROUP	wt_sal_female	wt_DMI_female	wt_MIL_female	KO_a1B_sal_female	KO_a1B_DMI_female	KO_a1B_MIL_female	wt_sal_female	wt_DMI_female	wt_MIL_female	KO_a1B_sal_female	KO_a1B_DMI_female	KO_a1B_MIL_female	wt_sal_female	wt_DMI_female	wt_MIL_female	KO_a1B_sal_female	KO_a1B_DMI_female	KO_a1B_MIL_female	wt_sal_female	wt_MIL_female	KO_a1B_sal_female	KO_a1B_DMI_female	KO_a1B_MIL_female	wt_DMI_female
ID	5101	5116	6447	5109	5110	6484	5102	5117	6448	5112	5111	6485	5104	5118	6449	5127	5124	6486	5163	6450	5128	5125	6487	5145
GEL#10																								
BAND from left	1	2	3	4	5	6	7	8	9	10	11	12	13	14	15	16	17	18	19	20	21	22	23	24
GROUP	wt_sal_female	wt_DMI_female	wt_MIL_female	KO_a1B_sal_female	KO_a1B_DMI_female	KO_a1B_MIL_female	wt_sal_female	wt_DMI_female	wt_MIL_female	KO_a1B_sal_female	KO_a1B_DMI_female	KO_a1B_MIL_female	wt_sal_female	wt_DMI_female	wt_MIL_female	KO_a1B_sal_female	KO_a1B_DMI_female	KO_a1B_MIL_female	wt_sal_female	wt_DMI_female	wt_MIL_female	KO_a1B_sal_female	KO_a1B_DMI_female	KO_a1B_MIL_female
ID	5164	5158	6493	5129	5133	6488	6434	5159	6494	5130	5137	6489	6435	6462	6495	6417	6473	6502	6436	6497	6496	6454	6475	6503
GEL#11																								
BAND from left	1	2	3	4	5	6	7	8	9	10	11	12	13	14	15	16	17	18	19	20	21	22	23	24
GROUP	wt_sal_female	wt_DMI_female	wt_MIL_female	KO_a1B_sal_female	KO_a1B_DMI_female	KO_a1B_MIL_female	wt_sal_female	wt_DMI_female	wt_MIL_female	KO_a1B_sal_female	KO_a1B_DMI_female	KO_a1B_MIL_female	wt_sal_female	wt_DMI_female	wt_MIL_female	KO_a1B_sal_female	KO_a1B_DMI_female	KO_a1B_MIL_female	wt_sal_female	wt_DMI_female	wt_MIL_female	KO_a1B_sal_female	KO_a1B_DMI_female	KO_a1B_MIL_female
ID	5101	5116	6447	5109	5110	6484	5102	5117	6448	5112	5111	6485	5104	5118	6449	5127	5124	6486	5163	5145	6450	5128	5125	6487

p AKT from up to bottom: GEL#9, GEL#10; GEL#11

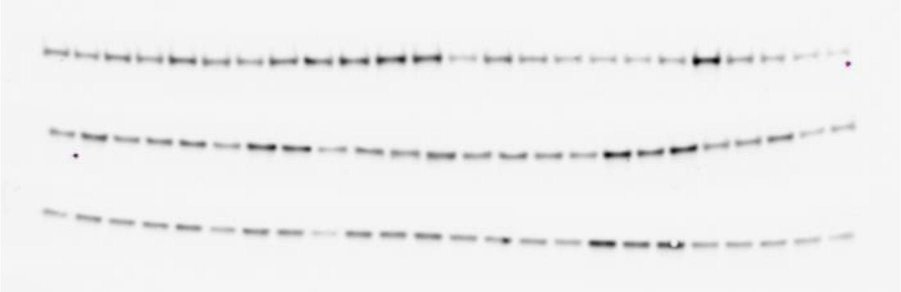

AKT from up to bottom: GEL#9, GEL#10; GEL#11

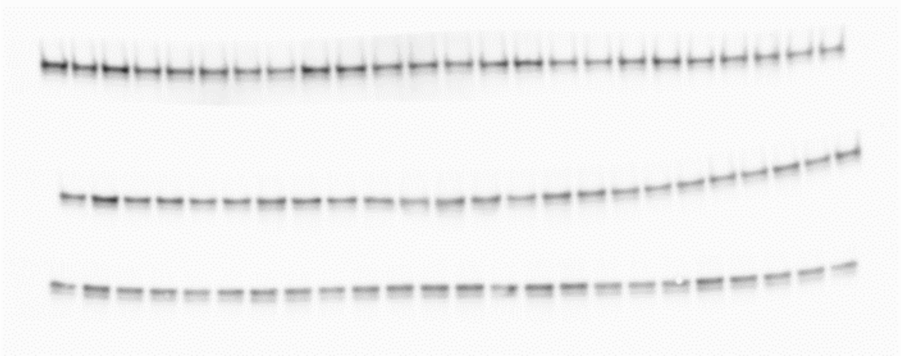

βACTIN from up to bottom: GEL#9, GEL#10; GEL#11

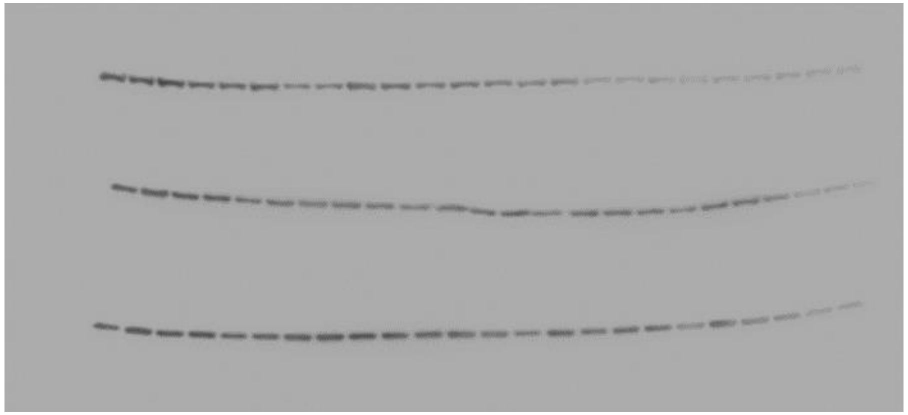

p/t AKT PROTEINS in D-KO MALE mice:

GEL#30

pAkt (upper blot) / tAkt (middle) / CNX (bottom)

GEL#30																								
Band	1	2	3	4	5	6	7	8	9	10	11	12	13	14	15	16	17	18	19	20	21	22	23	24
GROUP	wt_sal_male	wt_DMI_male	wt_MIL_male	KO_a1D_sal_male	KO_a1D_DMI_male	KO_a1D_MIL_male	wt_sal_male	wt_DMI_male	wt_MIL_male	KO_a1D_sal_male	KO_a1D_DMI_male	KO_a1D_MIL_male	wt_sal_male	wt_DMI_male	wt_MIL_male	KO_a1D_sal_male	KO_a1D_DMI_male	KO_a1D_MIL_male	wt_sal_male	wt_DMI_male	wt_MIL_male	KO_a1D_sal_male	KO_a1D_DMI_male	KO_a1D_MIL_male
ID	7050	7078	7741	7060	7095	7749	7051	7086	7742	7062	7096	7754	7066	7123	7753	7067	7121	7756	7068	7126	7771	7070	7132	7757

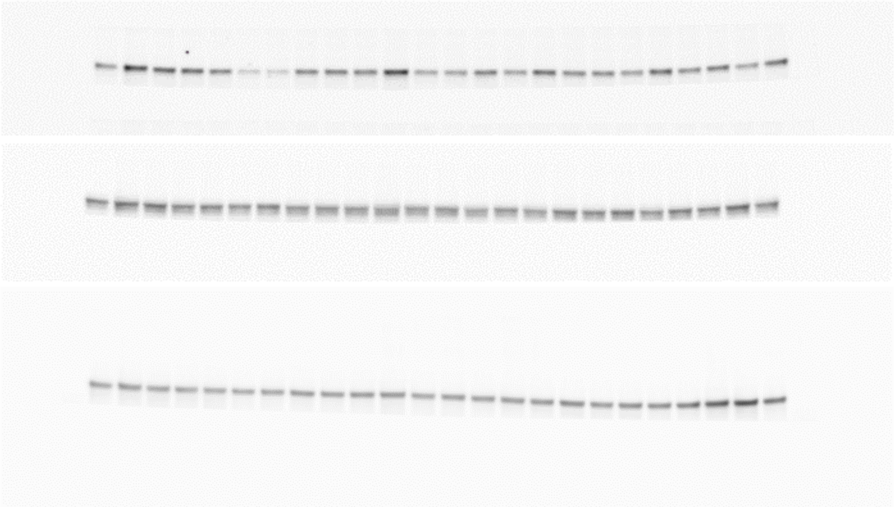

GEL#31

pAkt (upper blot) / tAkt (middle) / CNX (bottom)

GEL#31																								
Band	1	2	3	4	5	6	7	8	9	10	11	12	13	14	15	16	17	18	19	20	21	22	23	24
Grupa	wt_sal_male	wt_DMI_male	wt_MIL_male	KO_a1D_sal_male	KO_a1D_DMI_male	KO_a1D_MIL_male	wt_sal_male	wt_DMI_male	wt_MIL_male	KO_a1D_sal_male	KO_a1D_DMI_male	KO_a1D_MIL_male	wt_sal_male	wt_DMI_male	wt_MIL_male	KO_a1D_sal_male	KO_a1D_DMI_male	KO_a1D_MIL_male	wt_sal_male	wt_DMI_male	wt_MIL_male	KO_a1D_sal_male	KO_a1D_DMI_male	KO_a1D_MIL_male
ID	7050	7078	7741	7060	7095	7749	7051	7086	7742	7062	7096	7754	7066	7123	7753	7067	7121	7756	7068	7126	7771	7070	7132	7757

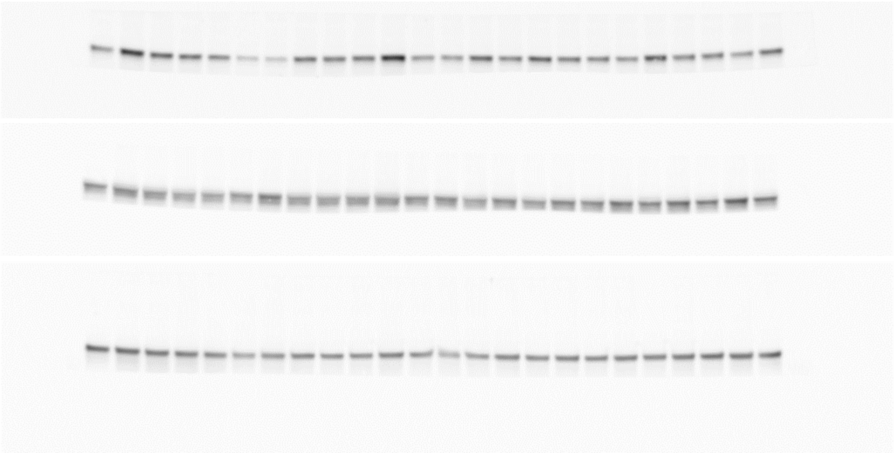

GEL#32

pAkt (upper blot) / tAkt (middle) / CNX (bottom)

GEL#32																								
Band	1	2	3	4	5	6	7	8	9	10	11	12	13	14	15	16	17	18	19	20	21	22	23	24
GROUP	wt_sal_male	wt_DMI_male	wt_MIL_male	KO_a1D_sal_male	KO_a1D_DMI_male	KO_a1D_MIL_male	wt_sal_male	wt_DMI_male	wt_MIL_male	KO_a1D_sal_male	KO_a1D_DMI_male	KO_a1D_MIL_male	wt_sal_male	wt_DMI_male	wt_MIL_male	KO_a1D_sal_male	KO_a1D_DMI_male	KO_a1D_MIL_male	wt_sal_male	wt_DMI_male	wt_MIL_male	KO_a1D_sal_male	KO_a1D_DMI_male	KO_a1D_MIL_male
ID	7100	7148	7774	7071	7133	7749	7101	7158	7818	7109	7142	7754	7102	7162	7823	7110	7149	7756	7103	7162	7826	7787	7794	7757

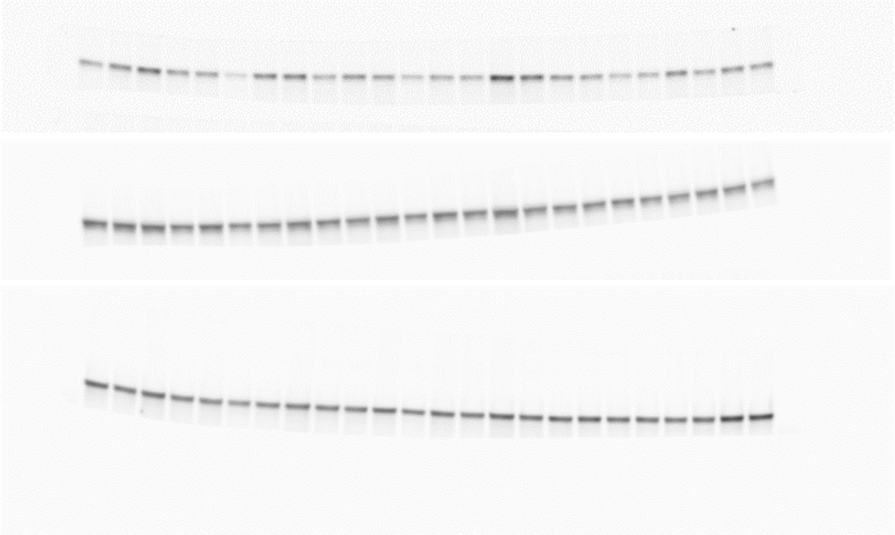

GEL#33

pAkt (upper blot) / tAkt (middle) / CNX (bottom)

GEL#33																								
BAND	1	2	3	4	5	6	7	8	9	10	11	12	13	14	15	16	17	18	19	20	21	22	23	24
Grupa	wt_sal_male	wt_DMI_male	wt_MIL_male	KO_a1D_sal_male	KO_a1D_DMI_male	KO_a1D_MIL_male	wt_sal_male	wt_DMI_male	wt_MIL_male	KO_a1D_sal_male	KO_a1D_DMI_male	KO_a1D_MIL_male	wt_sal_male	wt_DMI_male	wt_MIL_male	KO_a1D_sal_male	KO_a1D_DMI_male	KO_a1D_MIL_male	wt_sal_male	wt_DMI_male	wt_MIL_male	KO_a1D_sal_male	KO_a1D_DMI_male	KO_a1D_MIL_male
ID	7100	7148	7774	7071	7133	7749	7101	7158	7818	7109	7142	7754	7102	7162	7823	7110	7149	7756	7103	7162	7826	7787	7794	7757

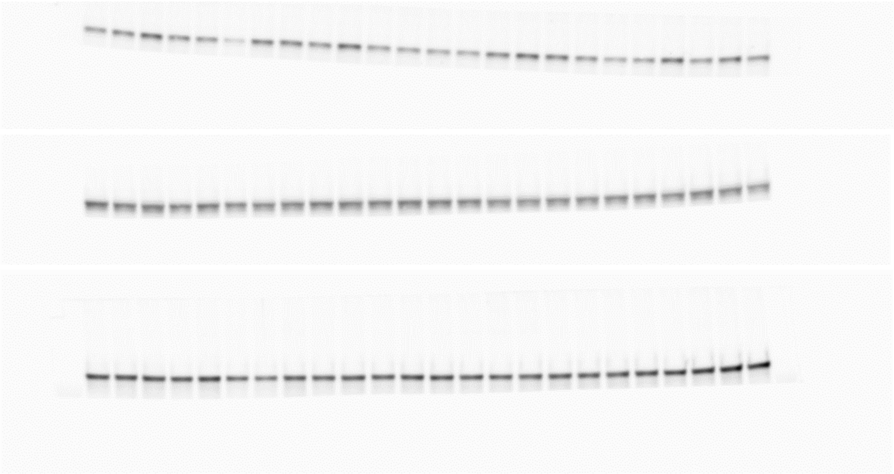

p/t AKT PROTEINS in D-KO FEMALE mice:

GEL#34

pAkt (upper blot) / tAkt (middle) / CNX (bottom)

GEL#34																								
Band	1	2	3	4	5	6	7	8	9	10	11	12	13	14	15	16	17	18	19	20	21	22	23	24
GROUP	wt_sal_female	wt_DMI_female	wt_MIL_female	KO_a1D_sal_female	KO_a1D_DMI_female	KO_a1D_MIL_female	wt_sal_female	wt_DMI_female	wt_MIL_female	KO_a1D_sal_female	KO_a1D_DMI_female	KO_a1D_MIL_female	wt_sal_female	wt_DMI_female	wt_MIL_female	KO_a1D_sal_female	KO_a1D_DMI_female	KO_a1D_MIL_female	wt_sal_female	wt_DMI_female	wt_MIL_female	KO_a1D_sal_female	KO_a1D_DMI_female	KO_a1D_MIL_female
ID	7052	7080	7775	7072	7097	7751	7053	7081	7777	7073	7098	7758	7054	7082	7800	7074	7099	7792	7055	7083	7801	7075	7116	7797

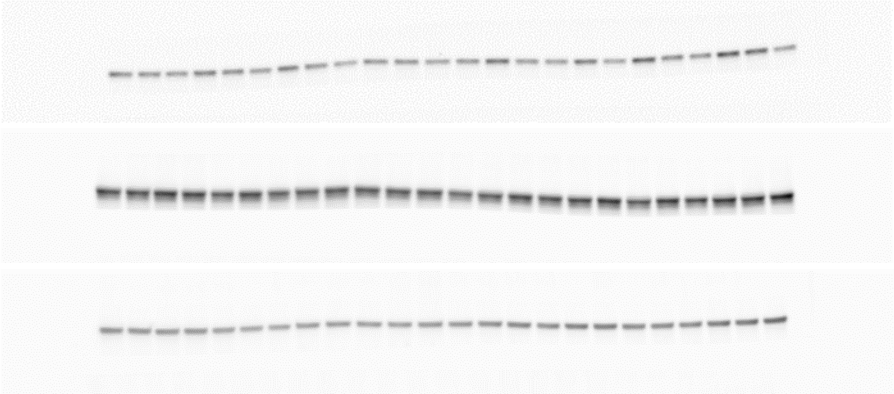

GEL#35

pAkt (upper blot) / tAkt (middle) / CNX (bottom)

GEL#35																								
Band	1	2	3	4	5	6	7	8	9	10	11	12	13	14	15	16	17	18	19	20	21	22	23	24
Grupa	wt_sal_female	wt_DMI_female	wt_MIL_female	KO_a1D_sal_female	KO_a1D_DMI_female	KO_a1D_MIL_female	wt_sal_female	wt_DMI_female	wt_MIL_female	KO_a1D_sal_female	KO_a1D_DMI_female	KO_a1D_MIL_female	wt_sal_female	wt_DMI_female	wt_MIL_female	KO_a1D_sal_female	KO_a1D_DMI_female	KO_a1D_MIL_female	wt_sal_female	wt_DMI_female	wt_MIL_female	KO_a1D_sal_female	KO_a1D_DMI_female	KO_a1D_MIL_female
ID	7052	7080	7775	7072	7097	7751	7053	7081	7777	7073	7098	7758	7054	7082	7800	7074	7099	7792	7055	7083	7801	7075	7116	7797

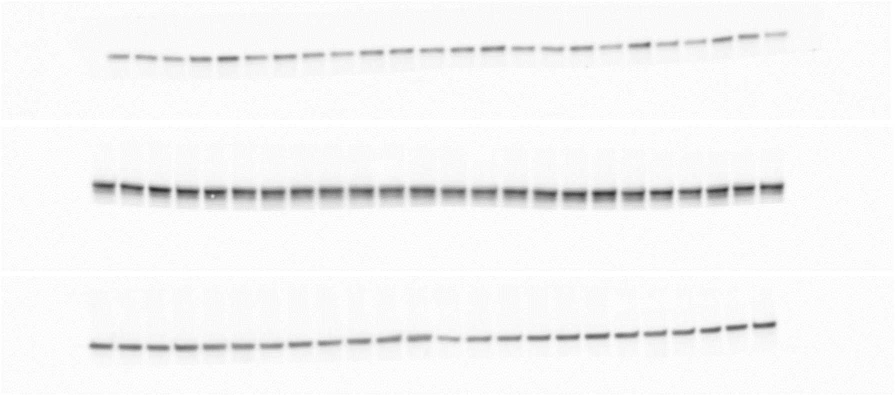

GEL#36

pAkt (upper blot) / tAkt (middle) / CNX (bottom)

GEL#36																								
Band	1	2	3	4	5	6	7	8	9	10	11	12	13	14	15	16	17	18	19	20	21	22	23	24
Grupa	wt_sal_female	wt_DMI_female	wt_MIL_female	KO_a1D_sal_female	KO_a1D_DMI_female	KO_a1D_MIL_female	wt_sal_female	wt_DMI_female	wt_MIL_female	KO_a1D_sal_female	KO_a1D_DMI_female	KO_a1D_MIL_female	wt_sal_female	wt_DMI_female	wt_MIL_female	KO_a1D_sal_female	KO_a1D_DMI_female	KO_a1D_MIL_female	wt_sal_female	wt_DMI_female	wt_MIL_female	KO_a1D_sal_female	KO_a1D_DMI_female	KO_a1D_MIL_female
ID	7056	7104	7809	7076	7138	7804	7745	7105	7835	7094	7143	7814	7091	7106	7848	7764	7743	7820	7092	7107	7859	7833	7744	7840

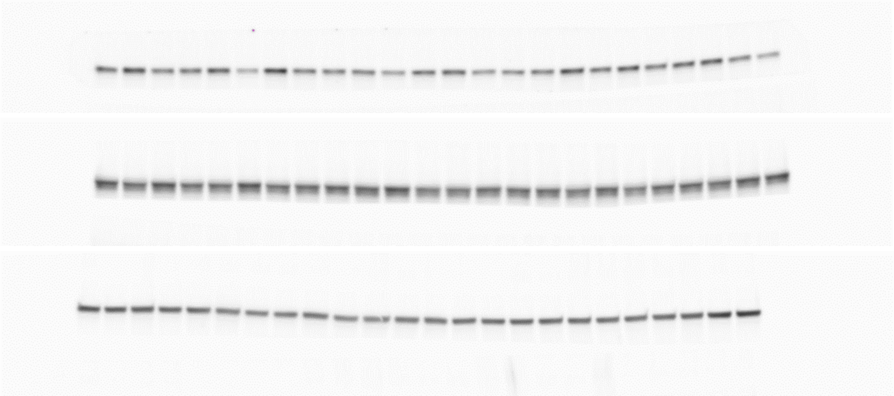

GEL#37

pAkt (upper blot) / tAkt (middle) / CNX (bottom)

GEL#37																								
Band	1	2	3	4	5	6	7	8	9	10	11	12	13	14	15	16	17	18	19	20	21	22	23	24
Grupa	wt_sal_female	wt_DMI_female	wt_MIL_female	KO_a1D_sal_female	KO_a1D_DMI_female	KO_a1D_MIL_female	wt_sal_female	wt_DMI_female	wt_MIL_female	KO_a1D_sal_female	KO_a1D_DMI_female	KO_a1D_MIL_female	wt_sal_female	wt_DMI_female	wt_MIL_female	KO_a1D_sal_female	KO_a1D_DMI_female	KO_a1D_MIL_female	wt_sal_female	wt_DMI_female	wt_MIL_female	KO_a1D_sal_female	KO_a1D_DMI_female	KO_a1D_MIL_female
ID	7056	7104	7809	7076	7138	7804	7745	7105	7835	7094	7143	7814	7091	7106	7848	7764	7743	7820	7092	7107	7859	7833	7744	7840

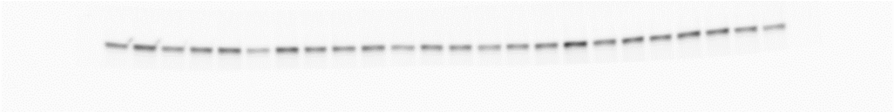

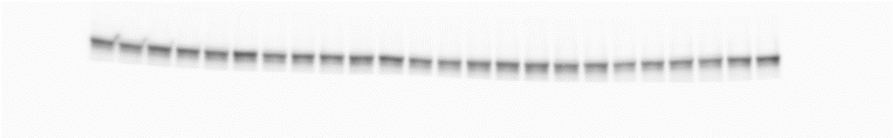

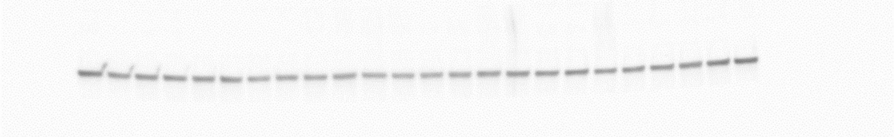

p/t GSK3β PROTEINS in HP, A-KO, male and female samples

GEL#5

pGSK3β (upper blot) / tGSK3β (middle) / CNX (bottom)

GEL#5																								
Band	1	2	3	4	5	6	7	8	9	10	11	12	13	14	15	16	17	18	19	20	21	22	23	24
GROUP	wt_sal_male	wt_DMI_male	wt_MIL_male	KO_a1A_sal_male	KO_a1A_DMI_male	KO_a1A_MIL_male	wt_sal_female	wt_DMI_female	wt_MIL_female	KO_a1A_sal_female	KO_a1A_DMI_female	KO_a1A_MIL_female	wt_sal_male	wt_DMI_male	wt_MIL_male	KO_a1A_sal_male	KO_a1A_DMI_male	KO_a1A_MIL_male	wt_sal_female	wt_DMI_female	wt_MIL_female	KO_a1A_sal_female	KO_a1A_DMI_female	KO_a1A_MIL_female
ID	6530	7026	7901	6537	6579	7886	6532	7003	7910	6558	6584	7895	6539	7028	7902	6553	6580	7888	6533	7004	7928	6559	6594	7897

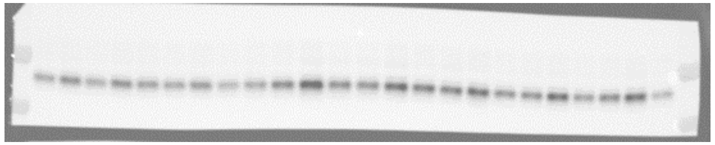

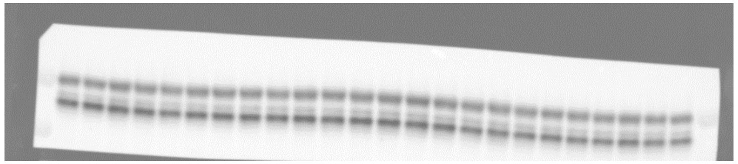

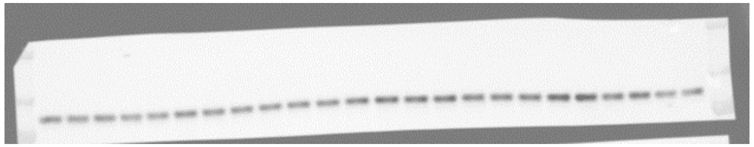

GEL#6

pGSK3β (upper blot) / tGSK3β (middle) / CNX (bottom)

GEL#6																								
Band	1	2	3	4	5	6	7	8	9	10	11	12	13	14	15	16	17	18	19	20	21	22	23	24
Grupa	wt_sal_male	wt_DMI_male	wt_MIL_male	KO_a1A_sal_male	KO_a1A_DMI_male	KO_a1A_MIL_male	wt_sal_female	wt_DMI_female	wt_MIL_female	KO_a1A_sal_female	KO_a1A_DMI_female	KO_a1A_MIL_female	wt_sal_male	wt_DMI_male	wt_MIL_male	KO_a1A_sal_male	KO_a1A_DMI_male	KO_a1A_MIL_male	wt_sal_female	wt_DMI_female	wt_MIL_female	KO_a1A_sal_female	KO_a1A_DMI_female	KO_a1A_MIL_female
ID	6546	7036	7906	6554	6586	7905	6551	7005	7983	6565	6596	7927	6547	7043	7935	6555	7024	7950	6552	7006	7984	6566	6598	7955

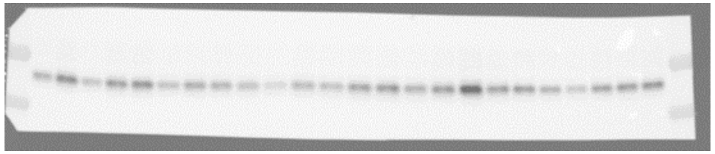

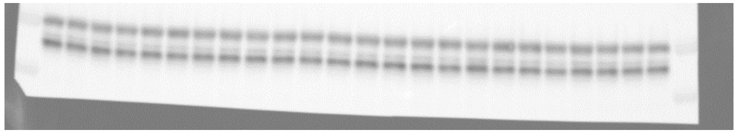

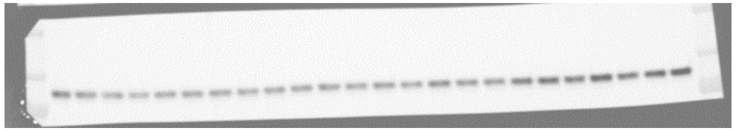

GEL#19

pGSK3β (upper blot) / tGSK3β (middle) / CNX (bottom)

GEL#19												
Band	1	2	3	4	5	6	7	8	9	10	11	12
Grupa	wt_sal_male	wt_DMI_male	wt_MIL_male	KO_a1A_sal_male	KO_a1A_DMI_male	KO_a1A_MIL_male	wt_sal_female	wt_DMI_female	wt_MIL_female	KO_a1A_sal_female	KO_a1A_DMI_female	KO_a1A_MIL_female
ID	failed	failed	7899	6513	6572	7870	6523	6590	7908	6543	6511	7863

GEL#19												
Band	13	14	15	16	17	18	19	20	21	22	23	24
Grupa	wt_sal_male	wt_DMI_male	wt_MIL_male	KO_a1A_sal_male	KO_a1A_DMI_male	KO_a1A_MIL_male	wt_sal_female	wt_DMI_female	wt_MIL_female	KO_a1A_sal_female	KO_a1A_DMI_female	KO_a1A_MIL_female
ID	6527	6574	7900	6531	6573	7871	6524	6591	7909	6544	6576	7891

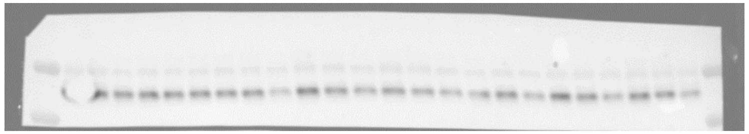

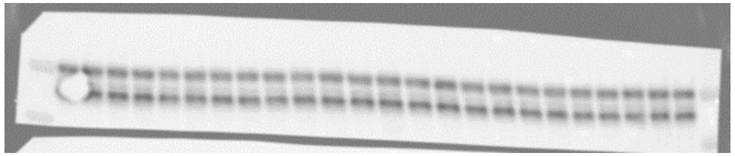

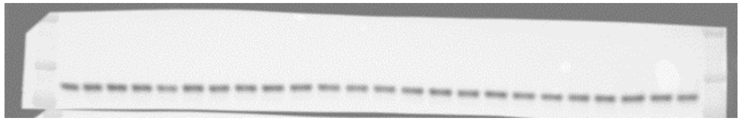

GEL#20

pGSK3β (upper blot) / tGSK3β (middle) / CNX (bottom)

GEL#20												
BAND	1	2	3	4	5	6	7	8	9	10	11	12
GROUP	wt_sal_male	wt_DMI_male	wt_MIL_male	KO_a1A_sal_male	KO_a1A_DMI_male	KO_a1A_MIL_male	wt_sal_female	wt_DMI_female	wt_MIL_female	KO_a1A_sal_female	KO_a1A_DMI_female	KO_a1A_MIL_female
ID	6530	7026	7901	6537	6579	7886	6532	7003	7910	6558	6584	7895

GEL#20												
BAND	13	14	15	16	17	18	19	20	21	22	23	24
GROUP	wt_sal_male	wt_DMI_male	wt_MIL_male	KO_a1A_sal_male	KO_a1A_DMI_male	KO_a1A_MIL_male	wt_sal_female	wt_DMI_female	wt_MIL_female	KO_a1A_sal_female	KO_a1A_DMI_female	KO_a1A_MIL_female
ID	6539	7028	7902	6553	6580	7888	6533	7004	7928	6559	6594	7897

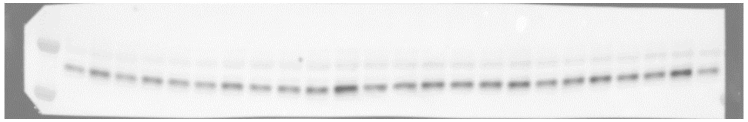

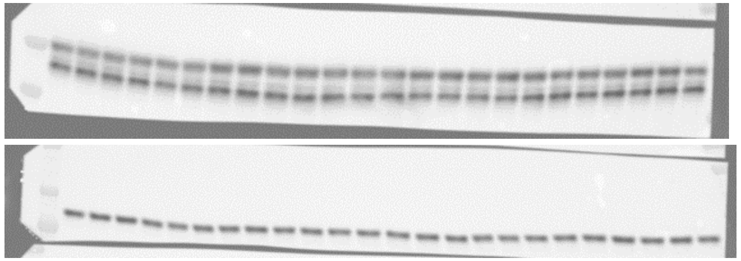

GEL#21

pGSK3β (upper blot) / tGSK3β (middle) / CNX (bottom)

GEL#21																								
Band	1	2	3	4	5	6	7	8	9	10	11	12	13	14	15	16	17	18	19	20	21	22	23	24
GROUP	wt_sal_male	wt_DMI_male	wt_MIL_male	KO_a1A_sal_male	KO_a1A_DMI_male	KO_a1A_MIL_male	wt_sal_female	wt_DMI_female	wt_MIL_female	KO_a1A_sal_female	KO_a1A_DMI_female	KO_a1A_MIL_female	wt_sal_male	wt_DMI_male	wt_MIL_male	KO_a1A_sal_male	KO_a1A_DMI_male	KO_a1A_MIL_male	wt_sal_female	wt_DMI_female	wt_MIL_female	KO_a1A_sal_female	KO_a1A_DMI_female	KO_a1A_MIL_female
ID	6546	7036	7906	6554	6586	7905	6551	7005	7983	6565	6596	7927	6547	7043	7935	6555	7024	7950	6552	7006	7984	6566	6598	7955

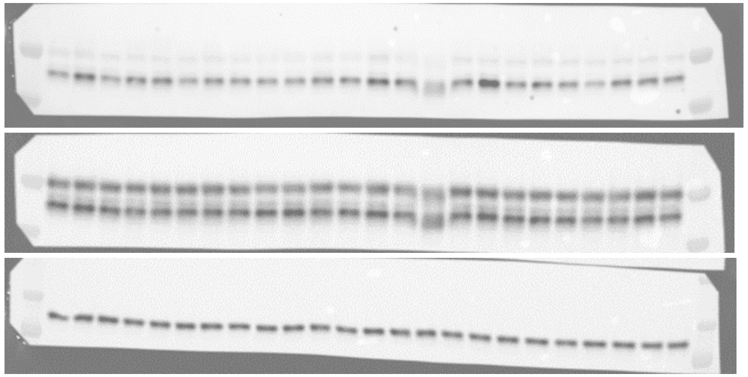

p/t GSK3β PROTEINS in D-KO

GEL#30

pGSK3β (upper blot) / tGSK3β (middle) / CNX (bottom)

GEL#30																								
Band	1	2	3	4	5	6	7	8	9	10	11	12	13	14	15	16	17	18	19	20	21	22	23	24
GROUP	wt_sal_male	wt_DMI_male	wt_MIL_male	KO_a1D_sal_male	KO_a1D_DMI_male	KO_a1D_MIL_male	wt_sal_male	wt_DMI_male	wt_MIL_male	KO_a1D_sal_male	KO_a1D_DMI_male	KO_a1D_MIL_male	wt_sal_male	wt_DMI_male	wt_MIL_male	KO_a1D_sal_male	KO_a1D_DMI_male	KO_a1D_MIL_male	wt_sal_male	wt_DMI_male	wt_MIL_male	KO_a1D_sal_male	KO_a1D_DMI_male	KO_a1D_MIL_male
ID	7050	7078	7741	7060	7095	7749	7051	7086	7742	7062	7096	7754	7066	7123	7753	7067	7121	7756	7068	7126	7771	7070	7132	7757

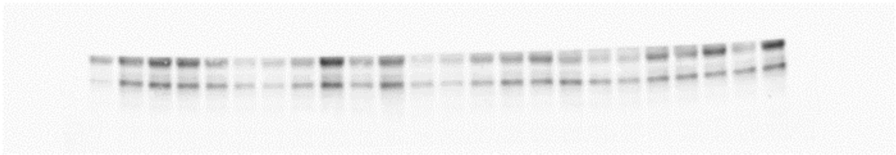

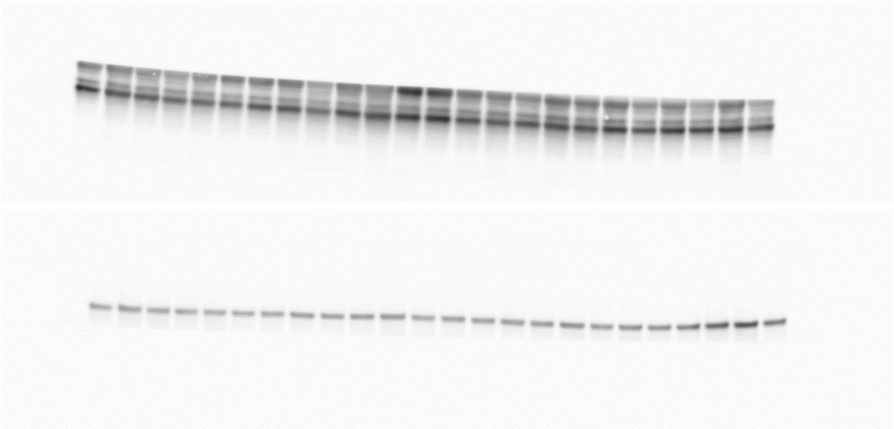

GEL#31

pGSK3β (upper blot) / tGSK3β (middle) / CNX (bottom)

GEL#31																								
Band	1	2	3	4	5	6	7	8	9	10	11	12	13	14	15	16	17	18	19	20	21	22	23	24
Grupa	wt_sal_male	wt_DMI_male	wt_MIL_male	KO_a1D_sal_male	KO_a1D_DMI_male	KO_a1D_MIL_male	wt_sal_male	wt_DMI_male	wt_MIL_male	KO_a1D_sal_male	KO_a1D_DMI_male	KO_a1D_MIL_male	wt_sal_male	wt_DMI_male	wt_MIL_male	KO_a1D_sal_male	KO_a1D_DMI_male	KO_a1D_MIL_male	wt_sal_male	wt_DMI_male	wt_MIL_male	KO_a1D_sal_male	KO_a1D_DMI_male	KO_a1D_MIL_male
ID	7050	7078	failed	7060	7095	7749	7051	7086	7742	7062	7096	7754	7066	7123	7753	7067	7121	7756	7068	7126	7771	7070	7132	7757

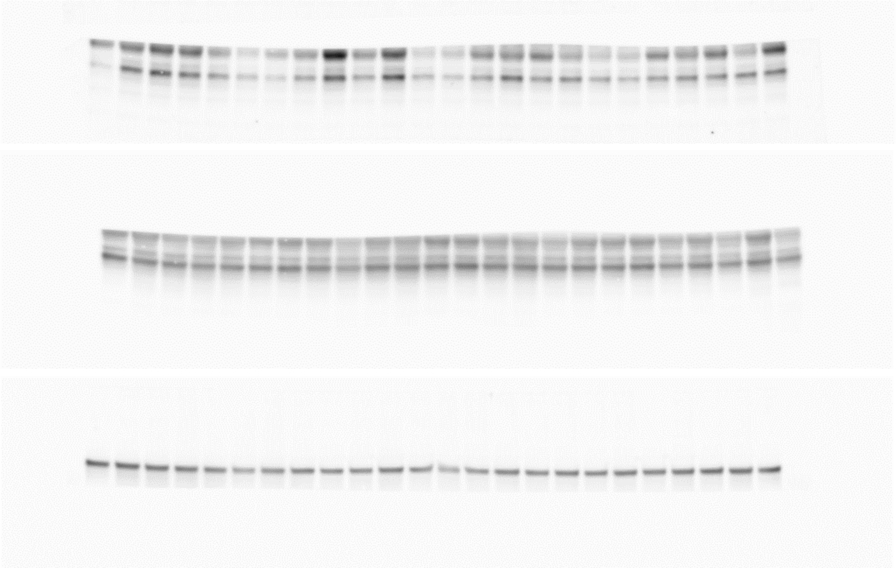

GEL#32

pGSK3β (upper blot) / tGSK3β (middle) / CNX (bottom)

GEL#32																								
Band	1	2	3	4	5	6	7	8	9	10	11	12	13	14	15	16	17	18	19	20	21	22	23	24
GROUP	wt_sal_male	wt_DMI_male	wt_MIL_male	KO_a1D_sal_male	KO_a1D_DMI_male	KO_a1D_MIL_male	wt_sal_male	wt_DMI_male	wt_MIL_male	KO_a1D_sal_male	KO_a1D_DMI_male	KO_a1D_MIL_male	wt_sal_male	wt_DMI_male	wt_MIL_male	KO_a1D_sal_male	KO_a1D_DMI_male	KO_a1D_MIL_male	wt_sal_male	wt_DMI_male	wt_MIL_male	KO_a1D_sal_male	KO_a1D_DMI_male	KO_a1D_MIL_male
ID	7100	7148	7774	7071	7133	7749	7101	7158	7818	7109	7142	7754	7102	7162	7823	7110	7149	7756	7103	7162	7826	7787	7794	7757

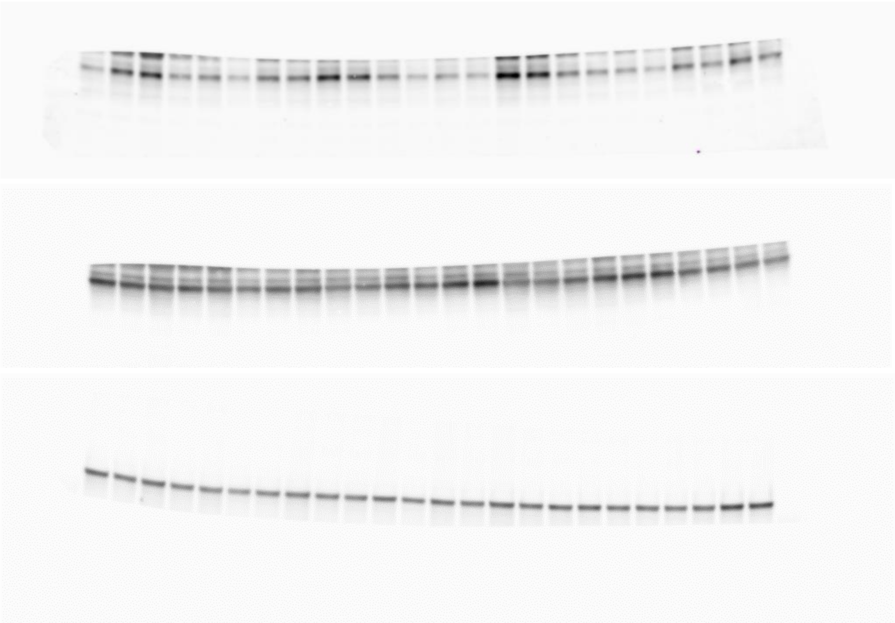

GEL#33

pGSK3β (upper blot) / tGSK3β (middle) / CNX (bottom)

GEL#33																								
Band	1	2	3	4	5	6	7	8	9	10	11	12	13	14	15	16	17	18	19	20	21	22	23	24
GROUP	wt_sal_ male	wt_DMI_ male	wt_MIL_ male	KO_a1D_sal_ male	KO_a1D_DMI _male	KO_a1D_MIL _male	wt_sal_ male	wt_DMI_ male	wt_MIL_ male	KO_a1D_sal _male	KO_a1D_DMI _male	KO_a1D_MIL _male	wt_sal_ male	wt_DMI_ male	wt_MIL_ male	KO_a1D_sal _male	KO_a1D_DMI _male	KO_a1D_MIL _male	wt_sal_ male	wt_DMI_ male	wt_MIL_ male	KO_a1D_sal _male	KO_a1D_DMI _male	KO_a1D_MIL _male
ID	7100	7148	7774	7071	7133	7749	7101	7158	7818	7109	7142	7754	7102	7162	7823	7110	7149	7756	7103	7162	7826	7787	7794	7757

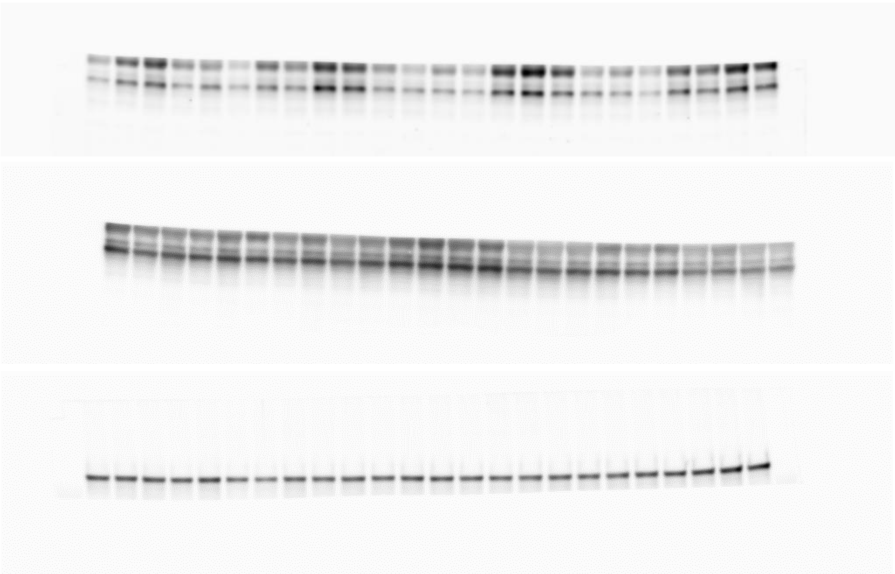

GEL#34

pGSK3β (upper blot) / tGSK3β (middle) / CNX (bottom)

GEL#34																								
Band	1	2	3	4	5	6	7	8	9	10	11	12	13	14	15	16	17	18	19	20	21	22	23	24
GROUP	wt_sal_female	wt_DMI_female	wt_MIL_female	KO_a1D_sal_female	KO_a1D_DMI_female	KO_a1D_MIL_female	wt_sal_female	wt_DMI_female	wt_MIL_female	KO_a1D_sal_female	KO_a1D_DMI_female	KO_a1D_MIL_female	wt_sal_female	wt_DMI_female	wt_MIL_female	KO_a1D_sal_female	KO_a1D_DMI_female	KO_a1D_MIL_female	wt_sal_female	wt_DMI_female	wt_MIL_female	KO_a1D_sal_female	KO_a1D_DMI_female	KO_a1D_MIL_female
ID	7052	7080	7775	7072	7097	7751	7053	7081	7777	7073	7098	7758	7054	7082	7800	7074	7099	7792	7055	7083	7801	7075	7116	7797

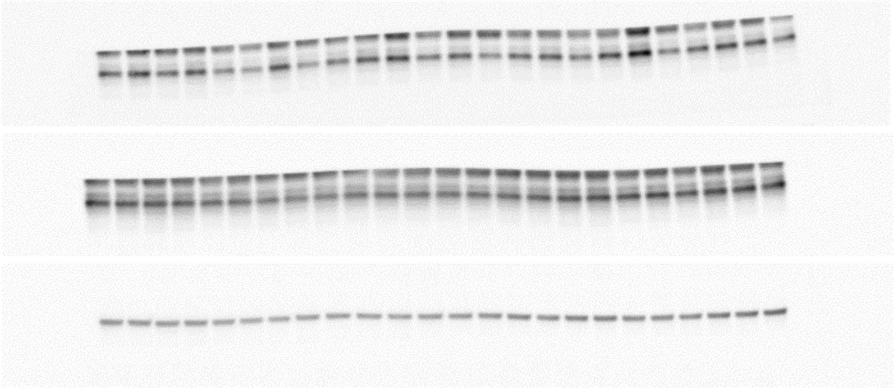

GEL#35

pGSK3β (upper blot) / tGSK3β (middle) / CNX (bottom)

GEL#35																								
Band	1	2	3	4	5	6	7	8	9	10	11	12	13	14	15	16	17	18	19	20	21	22	23	24
Grupa	wt_sal_female	wt_DMI_female	wt_MIL_female	KO_a1D_sal_female	KO_a1D_DMI_female	KO_a1D_MIL_female	wt_sal_female	wt_DMI_female	wt_MIL_female	KO_a1D_sal_female	KO_a1D_DMI_female	KO_a1D_MIL_female	wt_sal_female	wt_DMI_female	wt_MIL_female	KO_a1D_sal_female	KO_a1D_DMI_female	KO_a1D_MIL_female	wt_sal_female	wt_DMI_female	wt_MIL_female	KO_a1D_sal_female	KO_a1D_DMI_female	KO_a1D_MIL_female
ID	7052	7080	7775	7072	7097	7751	7053	7081	7777	7073	7098	7758	7054	7082	7800	7074	7099	7792	7055	7083	7801	7075	7116	7797

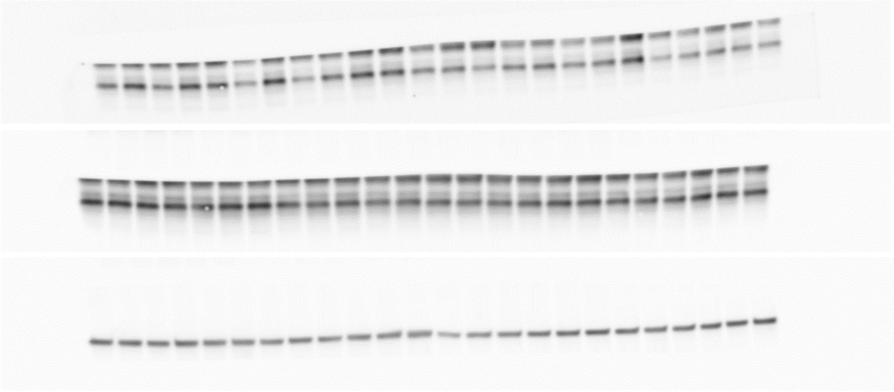

GEL#36

pGSK3β (upper blot) / tGSK3β (middle) / CNX (bottom)

GEL#36																								
Band	1	2	3	4	5	6	7	8	9	10	11	12	13	14	15	16	17	18	19	20	21	22	23	24
Grupa	wt_sal_female	wt_DMI_female	wt_MIL_female	KO_a1D_sal_female	KO_a1D_DMI_female	KO_a1D_MIL_female	wt_sal_female	wt_DMI_female	wt_MIL_female	KO_a1D_sal_female	KO_a1D_DMI_female	KO_a1D_MIL_female	wt_sal_female	wt_DMI_female	wt_MIL_female	KO_a1D_sal_female	KO_a1D_DMI_female	KO_a1D_MIL_female	wt_sal_female	wt_DMI_female	wt_MIL_female	KO_a1D_sal_female	KO_a1D_DMI_female	KO_a1D_MIL_female
ID	7056	7104	7809	7076	7138	7804	7745	7105	7835	7094	7143	7814	7091	7106	7848	7764	7743	7820	7092	7107	7859	7833	7744	7840

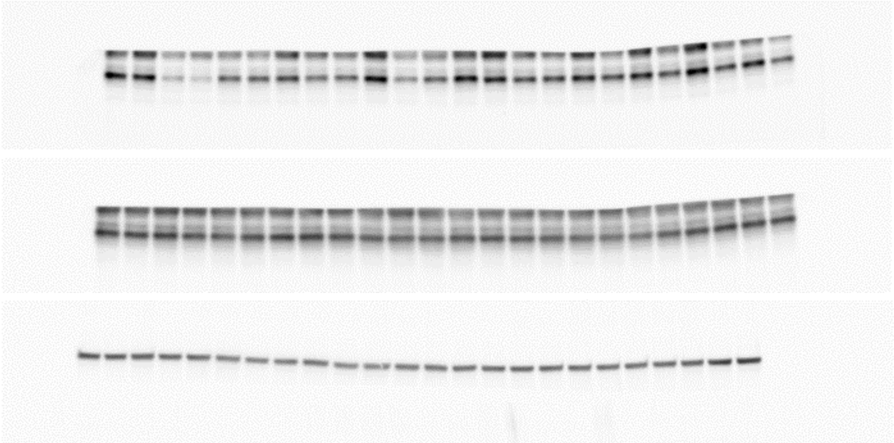

GEL#37

pGSK3β (upper blot) / tGSK3β (middle) / CNX (bottom)

GEL#37																								
Band	1	2	3	4	5	6	7	8	9	10	11	12	13	14	15	16	17	18	19	20	21	22	23	24
Grupa	wt_sal_ female	wt_DMI_ female	wt_MIL_ female	KO_a1D_sal_ female	KO_a1D_DMI _female	KO_a1D_MIL _female	wt_sal_ female	wt_DMI_ female	wt_MIL_ female	KO_a1D_sal_ female	KO_a1D_DMI _female	KO_a1D_MIL _female	wt_sal_ female	wt_DMI_ female	wt_MIL_ female	KO_a1D_sal _female	KO_a1D_D MI_female	KO_a1D_MIL_ female	wt_sal_ female	wt_DMI_ female	wt_MIL_ female	KO_a1D_sal _female	KO_a1D_D MI_female	KO_a1D_MIL _female
ID	7056	7104	7809	7076	7138	7804	7745	7105	7835	7094	7143	7814	7091	7106	7848	7764	7743	7820	7092	7107	7859	7833	7744	7840

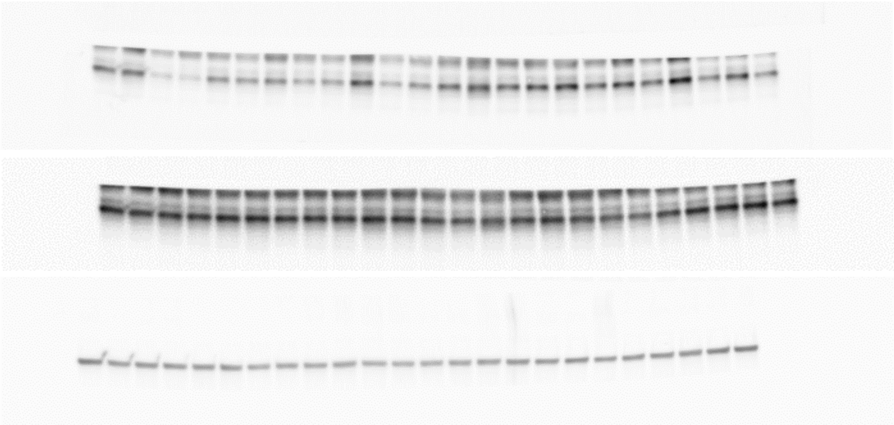

Supplement: Supplementary file 1 [file ijms-26-10488-s001.zip › Supplementary Figure S4_1027_Nalepa et al.pdf]
