# Supplementary material for: The Differential Involvement of α1-Adrenoceptor Subtypes in the Molecular Effects of Antidepressant Drugs
Source: Int J Mol Sci. 2025 Oct 28;26(21):10488. doi: 10.3390/ijms262110488 (PMC12610789; doi:10.3390/ijms262110488)
Supplement: Supplementary file 1 [file ijms-26-10488-s001.zip › Supplementary Table S1_1027_Nalepa et al.pdf]

The differential involvement of α1-adrenoceptor subtypes in the molecular effects of antidepressant drugs

Irena Nalepa [1*], Katarzyna Chorązka [1], Grzegorz Kreiner [1], Agnieszka Zelek-Molik [1], Anna Haduch [2], Władysława Anna Daniel [2], Piotr Chmielarz [1], Katarzyna Maziarz [1], Justyna Kuśmierczyk [1], Michał Wilczkowski [1], Adam Bielawski [1], Marta Kowalska [1]

[1]Department of Brain Biochemistry, Maj Institute of Pharmacology, Polish Academy of Sciences, Smętna 12, 31-343 Kraków, Poland; kreiner@if-pan.krakow.pl (G.K.); zelek@if-pan.krakow.pl (A.Z-M.); chmiel@if-pan.krakow.pl (P.C.); maziarz@if-pan.krakow.pl (K.M.); justyna.kusmierczyk@awf.krakow.pl (J.K.); wilczkow@if-pan.krakow.pl (M.W.); bielaw@if-pan.krakow.pl (A.B.); marcik48@op.pl (M.K.)

[2]Department of Pharmacokinetics and Drug Metabolism, Maj Institute of Pharmacology, Polish Academy of Sciences, Smętna 12, 31-343 Kraków, Poland; haduch@if-pan.krakow.pl (A.H.); nfdaniel@cyf-kr.edu.pl (W.A.D.);

*Correspondence: nfnalepa@cyf-kr.edu.pl

Supplementary Table S1. Some of the hippocampal transcripts that were significantly altered due to the deletion of one of the α1-AR subtypes—α1A (A-KO), α1B (B-KO), or α1D (D-KO)—compared to wild-type mice, and shared between two of the three transgenic lines: A-KO and B-KO (I), A-KO and D-KO (II), or B-KO and D-KO (III).

GenBank accession number	Gene symbol	Gene title	Fold change	P-value	Type of KO
I. (A-KO and B-KO)					
NM_206870	Ifna15	interferon alpha 15	0.236	<0.04	A-KO
			-0.753	<0.03	B-KO
NR_030645	Mir467e	microRNA 467e	-0.328	<0.05	A-KO
			-0.642	<0.03	B-KO
BC066147	2310067E19Rik	RIKEN cDNA 2310067E19 gene	-0.417	<0.05	A-KO
			-0.619	<0.01	B-KO
AK040372	Gm10002	predicted gene 10002[#]	-0.746	<0.03	A-KO
			-0.318	<0.008	B-KO
AK040372	Gm10002	predicted gene 10002[#]	-0.746	<0.03	A-KO
			-0.318	<0.008	B-KO
AK037773	Gm10325	predicted gene 10325	-0.670	<0.0005	A-KO
			-0.427	<0.04	B-KO
XR_168529	Gm20236	predicted gene[#]	-0.509	<0.02	A-KO
			-0.463	<0.002	B-KO

XR_168529	Gm20236	predicted gene#	-0.509	<0.02	A-KO
			-0.463	<0.002	B-KO
NM_016677	Hpcal1	hippocalcin-like 1	0.508	<0.05	A-KO
			0.452	<0.02	B-KO
II. (A-KO and D-KO)					
NR_035497	Mir1970	microRNA 1970	-0.679	<0.002	A-KO
			-0.475	<0.04	D-KO
AB010343	Gm10139	predicted gene 10139	-0.588	<0.0001	A-KO
			-0.486	<0.02	D-KO
NM_008143	Gnb2l1	guanine nucleotide binding protein (G protein)	-0.508	<0.03	A-KO
			-0.261	<0.05	D-KO
N/A	1110012L19Rik	RIKEN cDNA 1110012L19 gene	-0.504	<0.04	A-KO
			-0.573	<0.02	D-KO
AK136930	Gm4583	predicted gene 4583	1.182	<0.001	A-KO
			0.302	<0.05	D-KO
NM_013632	Pnp	purine-nucleoside phosphorylase	0.254	<0.03	A-KO
			-0.513	<0.03	D-KO
III. (B-KO and D-KO)					
NM_001033765	1700071K01Rik	RIKEN cDNA 1700071K01 gene	-1.016	<0.02	B-KO
			0.330	<0.05	D-KO
NR_035468	Mir1946a	microRNA 1946a	-0.731	<0.02	B-KO
			0.829	<0.002	D-KO
NM_001101647	Prlh	Prolactin-releasing hormone	-0.720	<0.03	B-KO
			0.246	<0.04	D-KO
NR_033299	9530091C08Rik	RIKEN cDNA 9530091C08 gene	-0.663	<0.001	B-KO
			0.687	<0.05	D-KO
NR_035468	Mir1946a	microRNA 1946a	-0.655	<0.008	B-KO
			0.875	<0.0005	D-KO
NM_010501	Ifit3	interferon-induced protein with tetratricopeptide repeats 3	-0.651	<0.002	B-KO
			0.473	<0.03	D-KO
NR_029823	Mir128-2	microRNA 128-2	-0.606	<0.03	B-KO
			0.510	<0.05	D-KO
NM_025273	Pcbd1	pterin 4 alpha carbinolamine dehydratase/dimerization cofactor of hepatocyte nuclear factor 1 alpha (TCF1) 1	0.509	<0.008	B-KO
			-0.529	<0.0003	D-KO
NM_016710	Hmgn5	high-mobility group nucleosome binding domain 5	0.640	<0.05	B-KO
			-0.210	<0.02	D-KO
NM_013725	Rps11	ribosomal protein S11	0.983	<0.04	B-KO
			-0.315	<0.006	D-KO

AK142452	Gm19304	predicted gene#	-0.344	<0.02	B-KO
			0.545	<0.03	D-KO
AK142452	Gm19304	predicted gene#	-0.344	<0.02	B-KO
			0.545	<0.03	D-KO
AK036170	5330427O13Rik	RIKEN cDNA 5330427O13 gene	-0.351	<0.009	B-KO
			0.604	<0.04	D-KO
NM_177845	Pla2g4e	phospholipase A2	-0.491	<0.05	B-KO
			0.877	<0.0005	D-KO

The significance threshold was established at $p < 0.05$ (t-test) and fold change > 1.5 (log2). A-KO - α1A, B-KO - α1B, D-KO - α1D knockout mice; N/A – not applicable; #for selected predicted genes, various probe sets detected different transcripts belonging to the same gene.
