# Supplementary material for: The Differential Involvement of α1-Adrenoceptor Subtypes in the Molecular Effects of Antidepressant Drugs
Source: Int J Mol Sci. 2025 Oct 28;26(21):10488. doi: 10.3390/ijms262110488 (PMC12610789; doi:10.3390/ijms262110488)
Supplement: Supplementary file 1 [file ijms-26-10488-s001.zip › Supplementary Table S2_1027_Nalepa et al.pdf]

The differential involvement of α1-adrenoceptor subtypes in the molecular effects of antidepressant drugs

Irena Nalepa [1*], Katarzyna Chorązka [1], Grzegorz Kreiner [1], Agnieszka Zelek-Molik [1], Anna Haduch [2], Władysława Anna Daniel [2], Piotr Chmielarz [1], Katarzyna Maziarz [1], Justyna Kuśmierczyk [1], Michał Wilczkowski [1], Adam Bielawski [1], Marta Kowalska [1]

[1]Department of Brain Biochemistry, Maj Institute of Pharmacology, Polish Academy of Sciences, Smętna 12, 31-343 Kraków, Poland; kreiner@if-pan.krakow.pl (G.K.); zelek@if-pan.krakow.pl (A.Z-M.); chmiel@if-pan.krakow.pl (P.C.); maziarz@if-pan.krakow.pl (K.M.); justyna.kusmierczyk@awf.krakow.pl (J.K.); wilczkow@if-pan.krakow.pl (M.W.); bielaw@if-pan.krakow.pl (A.B.); marcik48@op.pl (M.K.)

[2]Department of Pharmacokinetics and Drug Metabolism, Maj Institute of Pharmacology, Polish Academy of Sciences, Smętna 12, 31-343 Kraków, Poland; haduch@if-pan.krakow.pl (A.H.); nfdaniel@cyf-kr.edu.pl (W.A.D.);

*Correspondence: nfnalepa@cyf-kr.edu.pl

Supplementary Table S2. Some transcripts differentially expressed in the hippocampal tissue of wild-type mice were co-regulated by both chronic desipramine and milnacipran, compared to the saline-injected control group.

GenBank accession number	Gene symbol	Gene title	Fold change	P-value	Drug
NR_035423	Mir1192	microRNA 1192	1.141	<0.006	DMI
			0.675	<0.05	MIL
NR_002901	Snora68	small nucleolar RNA	-0.573	<0.04	DMI
			-0.773	<0.005	MIL
XM_001002242	Gm8430	predicted pseudogene 8430	-0.584	<0.02	DMI
			-0.578	<0.05	MIL
NR_028552	Snord58b	small nucleolar RNA	-0.683	<0.03	DMI
			-1.375	<0.00003	MIL
NR_028548	Snord16a	small nucleolar RNA	-0.705	<0.05	DMI
			-1.399	<0.00002	MIL
NR_028519	Scarna6	small Cajal body-specific RNA 6	-0.744	<0.05	DMI
			-1.361	<0.00003	MIL
NR_029412	Snora16a	small nucleolar RNA	-0.781	<0.04	DMI
			-1.264	<0.00003	MIL
NR_015524	Cep83os	centrosomal protein 83	-0.968	<0.02	DMI
			-0.638	<0.1	MIL
NR_003681	Snora15	small nucleolar RNA	-1.161	<0.04	DMI

			-2.440	<0.0006	MIL
NR_034051	Snora34	small nucleolar RNA	-1.200	<0.03	DMI
			-2.027	<0.00005	MIL
NR_046144	n-R5s136	nuclear encoded rRNA 5S 136	-1.657	<0.04	DMI
			-1.716	<0.02	MIL

The significance threshold was established at $p < 0.05$ (t-test) and fold change > 1.5 (log2). Drug – type of antidepressant drug administered; DMI—desipramine; MIL—milnacipran
