# Supplementary material for: The Differential Involvement of α1-Adrenoceptor Subtypes in the Molecular Effects of Antidepressant Drugs
Source: Int J Mol Sci. 2025 Oct 28;26(21):10488. doi: 10.3390/ijms262110488 (PMC12610789; doi:10.3390/ijms262110488)
Supplement: Supplementary file 1 [file ijms-26-10488-s001.zip › Supplementary Table S3_1027_Nalepa I. et al.pdf]

The differential involvement of α1-adrenoceptor subtypes in the molecular effects of antidepressant drugs

Irena Nalepa [1*], Katarzyna Chorązka [1], Grzegorz Kreiner [1], Agnieszka Zelek-Molik [1], Anna Haduch [2], Władysława Anna Daniel [2], Piotr Chmielarz [1], Katarzyna Maziarz [1], Justyna Kuśmierczyk [1], Michał Wilczkowski [1], Adam Bielawski [1], Marta Kowalska [1]

[1]Department of Brain Biochemistry, Maj Institute of Pharmacology, Polish Academy of Sciences, Smętna 12, 31-343 Kraków, Poland; kreiner@if-pan.krakow.pl (G.K.); zelek@if-pan.krakow.pl (A.Z-M.); chmiel@if-pan.krakow.pl (P.C.); maziarz@if-pan.krakow.pl (K.M.); justyna.kusmierczyk@awf.krakow.pl (J.K.); wilczkow@if-pan.krakow.pl (M.W.); bielaw@if-pan.krakow.pl (A.B.); marcik48@op.pl (M.K.)

[2]Department of Pharmacokinetics and Drug Metabolism, Maj Institute of Pharmacology, Polish Academy of Sciences, Smętna 12, 31-343 Kraków, Poland; haduch@if-pan.krakow.pl (A.H.); nfdaniel@cyf-kr.edu.pl (W.A.D.);

*Correspondence: nfnalepa@cyf-kr.edu.pl

Supplementary Table S3. The sequences of primers used in the qRT-PCR procedure

Gene	*Forward primer*	*final concentration*	*Reverse primer*	*final concentration*
ADRA1A	CTAAGGCCATTCTACTTGGGGT	50nM	CGAGTGCAGATGCCGATGA	50nM
ADRA1B	ATTGTAGTCGGAATGTTCATCTTA	200nM	GAAGTAGCCCAGCCAGAA	200nM
ADRA1D	CCACTTGCTCGCCCTGTG	400nM	AAAGTGACGCTCAGGATGT	100nM
HPRT	TCAGTCAACGGGGGACATAAA	200nM	GGGGCTGTACTGCTTAACCAG	200nM
Pgk1	GTTTGGAATGGTCCTGTTGGG	200nM	AGTGCTCACATGGCTGACTTTA	200nM
